# The pharmacological effects and therapeutic potential of flavonoids in digestive diseases

**DOI:** 10.3389/fphar.2025.1684377

**Published:** 2026-03-26

**Authors:** Junkang Xu, Jin Zhang, Bo Yu, Cuizhe Liu, Lin Zhang

**Affiliations:** Hebei Province Key Laboratory of Research and Development for Chinese Medicine, Institute of Traditional Chinese Medicine, Chengde Medical University, Chengde, Hebei, China

**Keywords:** flavonoids, digestive diseases, pharmacological effects, anti-inflammation, antioxidation

## Abstract

**Background:**

Digestive system diseases remain highly prevalent globally and constitute a major cause of mortality and disability. They not only severely compromise individual health but also result in massive consumption of medical resources, exacerbating socioeconomic burdens, thus emerging as a critical global public health challenge requiring resolution. In the prevention and treatment of digestive system diseases, flavonoids exhibit therapeutic potential through multiple targets and pathways.

**Methods:**

This review retrieved and summarized relevant literature on flavonoids for the treatment of digestive diseases published between 2000 and 2025 in databases including PubMed, Web of Science, Excerpta Medica Database, Wiley Online Library, SpringerLink, Nature Publishing Group, American Chemical Society, Elsevier, and Royal Society of Chemistry. Keywords (flavonoids, digestive diseases, pharmacological effects, anti-inflammation, antioxidation) were used for searching. During screening, priority was given to flavonoids directly related to the pathogenesis or intervention effects of digestive diseases, with study types covering reviews, clinical trials, randomized controlled trials, *etc.* Exclusion criteria included literature without clear association with digestive diseases and flavonoids, duplicate components and mechanisms, and lack of timeliness. Finally, 211 literature were screened and included from 13,000, providing references for subsequent research.

**Results:**

Flavonoids can prevent and treat digestive system diseases *via* multiple biological activities including anti-inflammation, antioxidation, anti-virus, anti-bacteria, anti-cancer, metabolic regulation and immune regulation.

**Conclusion:**

Although flavonoids exhibit significant pharmacological effects and favorable safety profiles in the prevention and treatment of digestive diseases, laying a foundation for the development of new drugs, they still face numerous challenges in clinical application. In the future, it is necessary to further conduct high-quality clinical studies, deepen research on the molecular mechanisms of their actions, and promote their translation from basic research to clinical practice.

## 1 Introduction

The human body is functionally dependent on the digestive system. The digestive system, which is crucial for the human body, has multiple functions. It mediates food digestion to facilitate nutrient absorption for energy metabolism and maintains the homeostasis of blood glucose, water, and electrolytes. Through mucous membranes, gastric acid, and intestinal flora, it can also build an immune barrier against pathogens. It is crucial for growth, development, and tissue repair, and its physiological integrity requires maintenance through a balanced diet and regular lifestyle. A variety of disorders which are common and often-occurring can afflict the digestive system. These disorders mainly involve the esophagus, the stomach, the small bowel, the large bowel, the liver, the gallbladder and the pancreas. Millions of people worldwide succumb to digestive system diseases each year^1^ (Zhao et al., 2025). Around half of all malignancies are digestive system malignancies (Yuan Yuan et al., 2022), such as esophageal cancer, gastric cancer, colorectal cancer, pancreatic cancer, hepatocellular carcinoma (HCC), and biliary tract cancer, which are the leading causes of cancer-related deaths globally (Qu et al., 2024). These diseases not only severely impair patients’ quality of life but also impose a heavy economic burden. The main clinical interventions for digestive system diseases are pharmacotherapy, surgery, endoscopic therapy and dietary regulation. For digestive system diseases, there are several main clinical treatment methods. Pharmacotherapy utilizes proton pump inhibitors, antibiotics, and biological agents to control symptoms and inflammation, but it is associated with risks of drug resistance, adverse reactions, and the need for long-term medication (Lamb et al., 2019; Khadivi et al., 2025). In cases of severe digestive system problems like digestive tract tumors and perforations, surgery can be used. However, it has problems such as causing significant trauma, delayed recovery, and many complications. Endoscopic therapy has the advantage of being minimally invasive when dealing with various lesions, but it is technically difficult and has risks of bleeding and perforation (Cassese et al., 2025). Dietary management is a fundamental approach, yet it is difficult to cure diseases and there are large individual differences in compliance.

Against the backdrop of integrating traditional and modern medicine, traditional Chinese medicine (TCM), with its centuries of clinical experience, has consistently demonstrated reliable efficacy in addressing complex health issues. The multi-component and multi-target synergistic effects, which are its advantages, offer well-proven perspectives on preventing and treating digestive system diseases. Throughout history, from traditional prescriptions to modern scientific adaptations, it has been protecting health in line with the concepts of preventive treatment of disease and treatment based on syndrome differentiation, which can be an inspiration for modern medicine. Flavonoids, which are widely found as important active substances in traditional medicine, possess anti-inflammatory, antioxidant and immunomodulatory capabilities, and are highly in line with the traditional medical concept of “regulating qi and blood and relieving pain with natural drugs”. Flavonoids can regulate inflammatory factors through multiple pathways, which has great therapeutic potential in digestive system diseases and can be used as a supplement or alternative to modern chemical drugs (An et al., 2025). Flavonoids can protect the integrity of the mucous membranes in the esophagus, stomach and intestines through suppressing the release of inflammatory mediators, promote the repair of damaged mucous membranes, stimulate mucus secretion and improve mucosal blood flow, thus reducing erosion (Zhao et al., 2020; Liu G. et al., 2019). Moreover, flavonoids can suppress the growth of various cancer cells and trigger their programmed cell death, which contributes to cancer treatment (Lee et al., 2024; Raha et al., 2015). Additionally, they reduce the risk of gallstone formation, relieve gallbladder smooth muscle spasm to alleviate pain, and delay the progression of liver diseases (Bae et al., 2020; Aziz et al., 2025). In the face of current challenges in the treatment of digestive system diseases, flavonoids are expected to become a key breakthrough point to overcome existing therapeutic bottlenecks, achieve efficient and low toxicity treatment, and bring new hope to patients.

Research advances in flavonoids for the treatment of digestive system diseases are systematically reviewed in this article. We deeply explored their action mechanisms, consolidated preclinical and clinical research results, and further discussed the current hurdles and future research directions in this area. By comprehensively collating the application potential of flavonoids in addressing digestive system diseases, theoretical groundwork for the development of novel, efficient and low-toxic therapeutic drugs is provided by this article, which is helpful for improving the treatment situation of patients with digestive system diseases.

**FIGURE 1:**
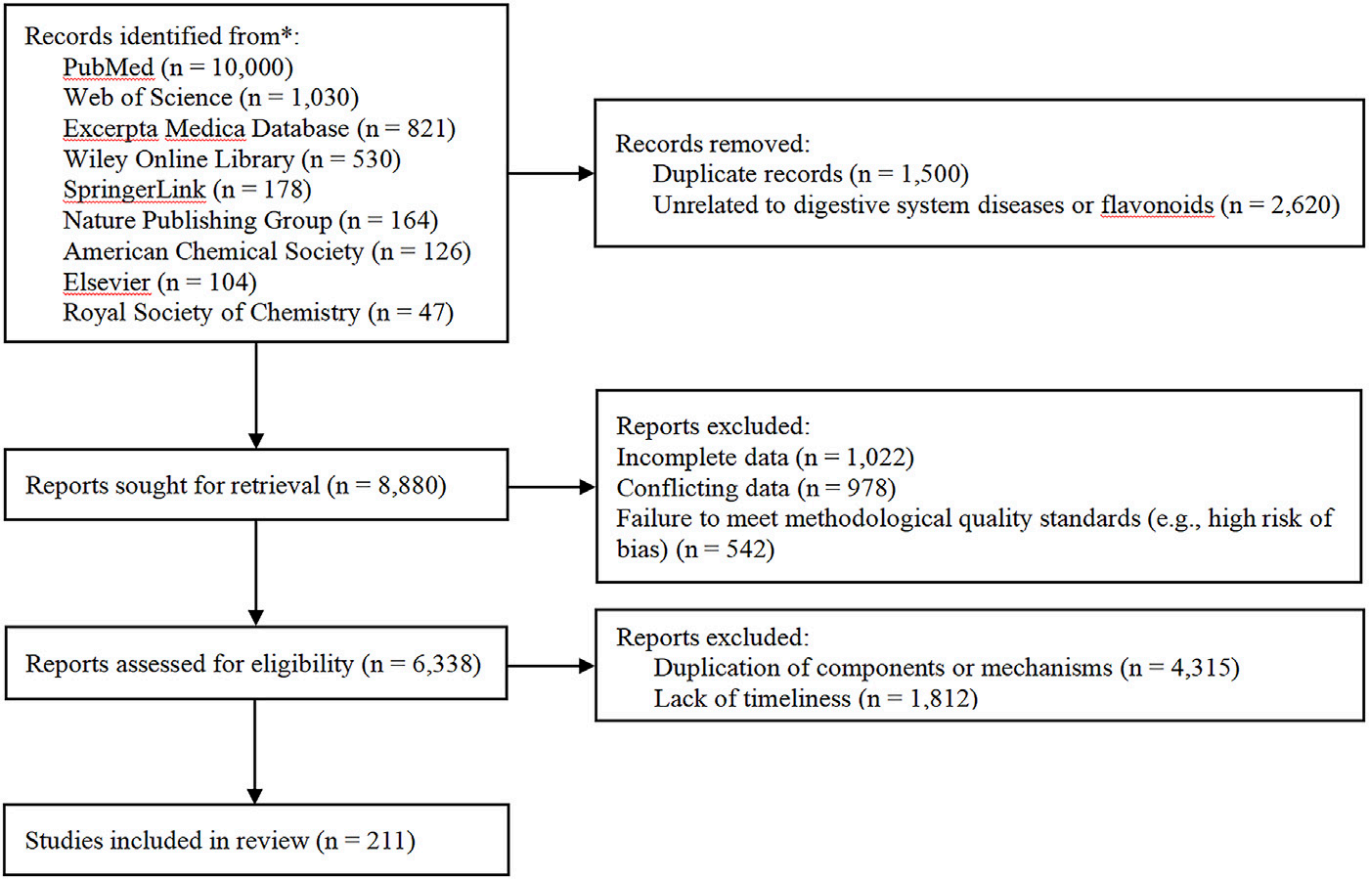
Flow diagram of the literature screening process.

We identified 13,000 studies through keyword search. We excluded duplicate articles (n = 1,500), studies unrelated to digestive system diseases and flavonoids (n = 2,620), studies with incomplete data (n = 1,022), conflicting data (n = 978), studies failing to meet methodological quality standards (e.g., high risk of bias) (n = 542), studies with duplicate components or mechanisms (n = 6,315), and studies lacking timeliness (n = 1,812). A total of 211 studies were finally included. A PRISMA flowchart illustrates the screening process (as shown in Figure 1). Subsequently, we conducted a quality assessment of the included studies. For *in vitro* cell experiments, the assessment focused on key points such as cell line traceability, randomization and balance of groups, and rationality of control setting. For animal model experiments, the analysis was performed around aspects including consistency between the model and the pathophysiology of clinical diseases, standardization of randomization and blinding, and scientificity of sample size estimation. For randomized controlled trials, the evaluation covered steps such as the scientificity of random sequence generation, completeness of allocation concealment, and rationality of blinding application. For systematic review studies, the systematic evaluation concentrated on key points including clarity of research questions and inclusion criteria, comprehensiveness and systematicness of literature search strategies, and scientificity of evidence synthesis methods.

## 2 The functions of flavonoids in preventing and managing digestive diseases

### 2.1 Esophageal diseases

The esophagus is a transport organ of the digestive system that moves food from the pharynx to the stomach *via* muscle peristalsis. The upper and lower esophageal sphincters have both anti-reflux and airway protection functions. As part of the cardia, the lower esophageal sphincter at the junction of the esophagus and stomach is a key barrier protecting the esophageal mucosa, and its dysfunction is prone to causing gastroesophageal reflux disease (Salvi et al., 2020). Dysbiosis of the microbial community in the esophagus and stomach may be associated with cancer. Although it has no direct connections with other organs, it interacts through the neuroendocrine system, the vagus nerve not only regulates its functions but also controls the secretion of gastric acid, pancreatic enzymes, and bile, as well as intestinal motility (Hasan et al., 2020; Du et al., 2025).

**FIGURE 2:**
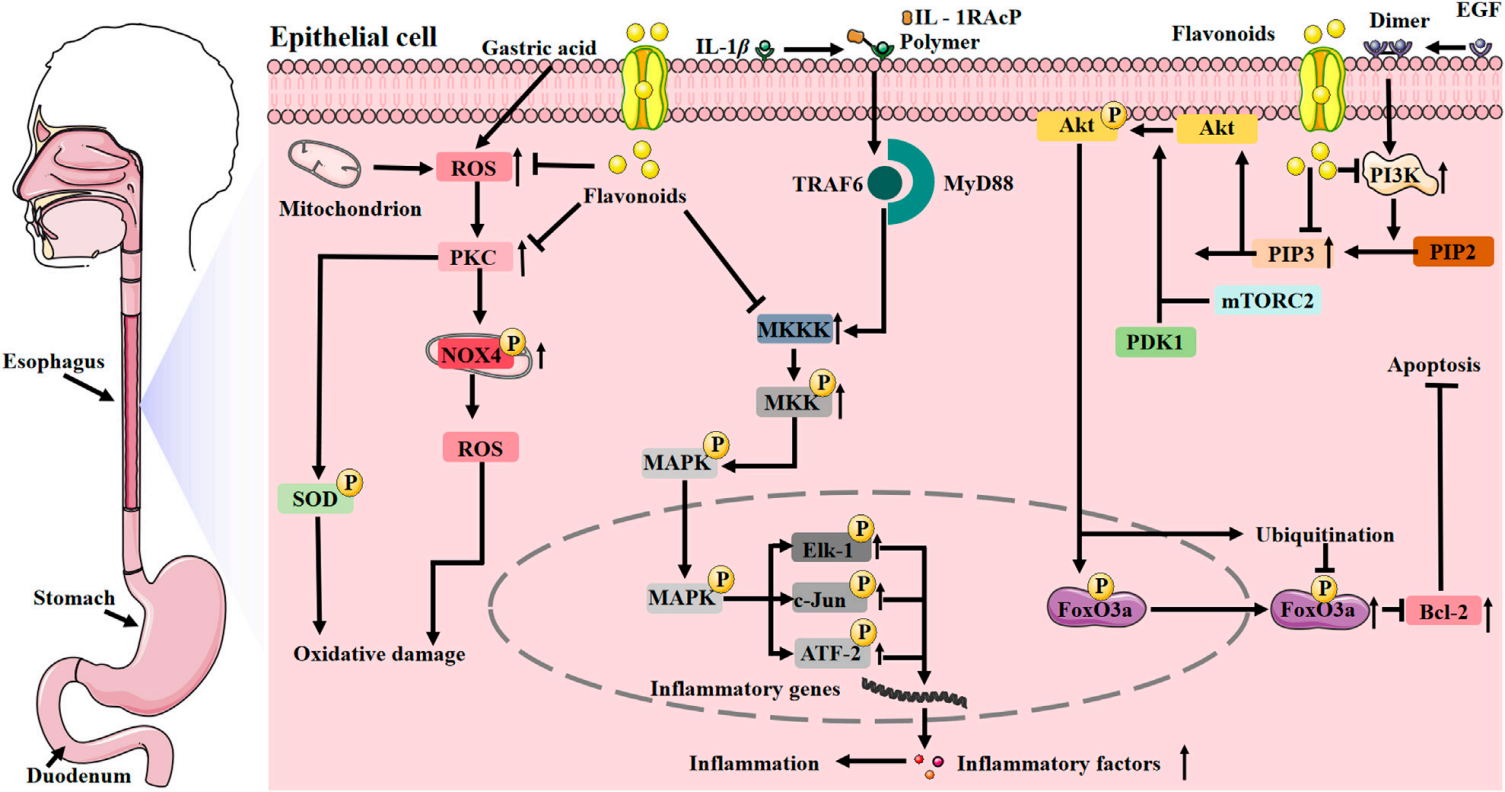
Mechanism of action of flavonoids in the treatment of esophageal diseases. Flavonoids can break the vicious cycle of “inflammation-oxidative stress” through dual mechanisms: on the one hand, they specifically inhibit MKKK activation to block the MAPK pathway, reducing the release of inflammatory factors; on the other hand, they suppress the activities of PKC and NOX1, restore SOD activity, and decrease ROS levels. Additionally, the “anti-inflammatory-antioxidant” synergistic effect of flavonoids can inhibit the PI3K/Akt pathway, thereby downregulating Bcl-2 expression to specifically induce tumor cell apoptosis. They can also protect normal cells from oxidative damage and inflammatory factor infiltration, thus preventing damaging apoptosis. Abbreviations: ATF-2, Activated Transcription Factor 2; ELK-1, Ets-like Protein 1; Foxo3a, Forkhead Box Protein O3a; IL-1RacP, Interleukin-1 Receptor Accessory Protein; PKC, protein kinase C; TRAF6, tumor necrosis factor receptor-associated factor 6; MyD88, myeloid differentiation factor 88; MKKK, mitogen-activated protein kinase kinase kinase; mTORC2, mammalian target of rapamycin complex 2; MKK, mitogen-activated protein kinase kinase; NOX4, NADPH oxidase 4; PIP3, phosphatidylinositol-3,4,5-trisphosphate; PIP2, phosphatidylinositol-4,5-bisphosphate; PDK1, 3-phosphatidylinositol-dependent protein kinase 1.

The esophagus, a food transport tract, can be affected by a collection of conditions referred to as esophageal diseases. These can lead to uncomfortable symptoms like difficulty in swallowing, heartburn, and pain. These symptoms have a severe influence on food consumption and nutrient assimilation, disturbing normal life and health (as shown in Figure 2).

Reflux esophagitis is characterized by esophageal inflammation and irritation resulting from the retrograde flow of gastric and duodenal contents into the esophagus, inducing inflammatory changes. There are symptoms such as a burning feeling and pain behind the breastbone, acid regurgitation, a burning sensation in the chest, belching, a feeling of blockage in the throat, and stomach discomfort. A flavonol compound known as quercetin exists. The viability of HET-1A cells which are stimulated by acid can be enhanced, and the secretion and mRNA production of cytokines like interleukin (IL)-6, IL-8, tumor necrosis factor-*α* (TNF-*α*), and IL-1*β* can be significantly inhibited. It can achieve this by suppressing certain proteins like c-jun N-terminal kinase, p38 mitogen-activated protein kinase (MAPK), and nuclear factor kappa-B (NF-*κ*B) proteins. By doing so, it relieves cellular inflammatory damage, which in turn has a therapeutic effect on reflux esophagitis (Zhao et al., 2022). Daidzein, an isoflavone compound, has a mechanism similar to quercetin in reducing inflammatory responses. Moreover, daidzein has the ability to boost the function of antioxidant enzymes like superoxide dismutase (SOD) and catalase. It can strengthen the body’s capacity to clear free radicals, decrease the build-up of substances such as hydrogen peroxide, calcium, and free iron in esophageal tissues. This can relieve oxidative damage, keep the physiological morphology and functionality of esophageal mucosal cells, and safeguard against esophageal damage caused by reflux esophagitis in rats (Den et al., 2021).

Eosinophilic esophagitis, a chronic inflammatory condition of the esophagus characterized by eosinophilic inflammation, leads to esophageal dysfunction. Vomiting, feeding difficulties, dysphagia, and abdominal pain are among the common symptoms. At present, dietary elimination and swallowed steroids are among the main treatment methods for eosinophilic esophagitis. A flavone compound known as 7,4′-dihydroxyflavone exists. It has the ability to restrain the production of eosinophil chemotactic factors, inflammation-promoting mediators, and immunoglobulin E, thus showing remarkable anti-inflammatory and immunomodulatory capabilities. Isoliquiritigenin, a chalcone compound, also relieves the pathological symptoms of eosinophilic esophagitis. It significantly inhibits upstream signaling pathways like NF-*κ*B, and decreases the levels of pro-inflammatory factors, cyclin D1 (CCND1), and MAPK1 (Maskey et al., 2022). 7,4′-dihydroxyflavone can do the same. Within a murine model of eosinophilic esophagitis resulting from dietary allergy, isoliquiritigenin has the ability to restrain the generation of T helper cell two inflammatory cytokines together with the expression of transforming growth factor-*β*1 (TGF-*β*1) in esophageal tissues. As a result, it can lessen the symptoms of eosinophilic esophagitis in model mice (Cao et al., 2022).

The principal type among esophageal cancer subtypes is esophageal squamous cell carcinoma (ESCC), and it generally has a not-so-good prognosis. It is a severe malignant tumor in the digestive system, which makes up 80% of esophageal cancer cases around the world (Zhang X. et al., 2025) and is a great threat to human health. All of luteolin, tangeretin, 5,7,4′-trimethoxyflavone, and cirsiliol belong to flavone compounds. Luteolin can halt cellular cycle and trigger programmed cell death *via* suppressing the multiplication, movement, intrusion, and formation of stem cell spheroids in paclitaxel-resistant ESCC cells. *In vitro*, when combined with paclitaxel, luteolin has the ability to attach to the catalytic sites of focal adhesion kinase (FAK), src family kinases, and protein kinase B (AKT), which then leads to the induction of cancer cell apoptosis. In addition, luteolin is able to diminish the tumor-forming ability of paclitaxel-resistant ESCC without causing notable toxicity, and intensify chemosensitivity *via* attenuating the FAK/phosphatidylinositol 3-kinase (PI3K)/AKT pathway, which is effective for ESCC treatment (Yang Z. et al., 2024). Tangeretin has the capacity to reduce tumor growth in xenografted mice. It reduces the expression and transcriptional activity of Glioma-Associated Oncogene (GLI) family zinc finger two in ESCC cells and downregulates the expression of glycoprotein non-metastatic melanoma protein B, which results in anti-migratory and anti-invasive effects (Yang D. et al., 2024). 5,7,4′-trimethoxyflavone is able to bind to leucine-rich PPR-motif containing protein (LRPPRC), signal transducer and activator of transcription (STAT) 3, and cyclin-dependent kinase (CDK) 1. By doing so, it can dissociate the interactions of LRPPRC-janus kinase (JAK)2-STAT3 and JAK2-STAT3-CDK1, disrupt protein complexes that promote ESCC progression, and then inhibit tumor growth. In addition, 5,7,4′-trimethoxyflavone is able to prevent tumorigenesis in an ESCC mouse model induced by 4-nitroquinoline N-oxide and restrain tumor growth in a patient-derived xenograft model of ESCC in mice (Liu H. et al., 2024). Cirsiliol can combine with tyrosine kinase 2 to suppress its activity, which in turn reduces signal transduction and the dimerization of STAT3, and eventually curbs the growth of ESCC in both *in vivo* environments and *in vitro* setting (Jia et al., 2021).

### 2.2 Gastric diseases

The stomach is an important organ of the digestive tract, with core functions including food storage, preliminary digestion, pathogen elimination, and regulation of chyme emptying (Du et al., 2025). The esophagus transports food to the stomach through peristalsis, while the lower esophageal sphincter is responsible for preventing the reflux of gastric contents, dysfunction of this part is prone to causing gastroesophageal reflux disease (Salvi et al., 2020). The stomach discharges the processed chyme into the duodenum, after which bile secreted by the liver and digestive enzymes secreted by the pancreas further break down nutrients. These nutrients are absorbed by the small intestine and eventually undergo metabolism and detoxification by the liver (Camilleri and Zheng, 2023).

**FIGURE 3:**
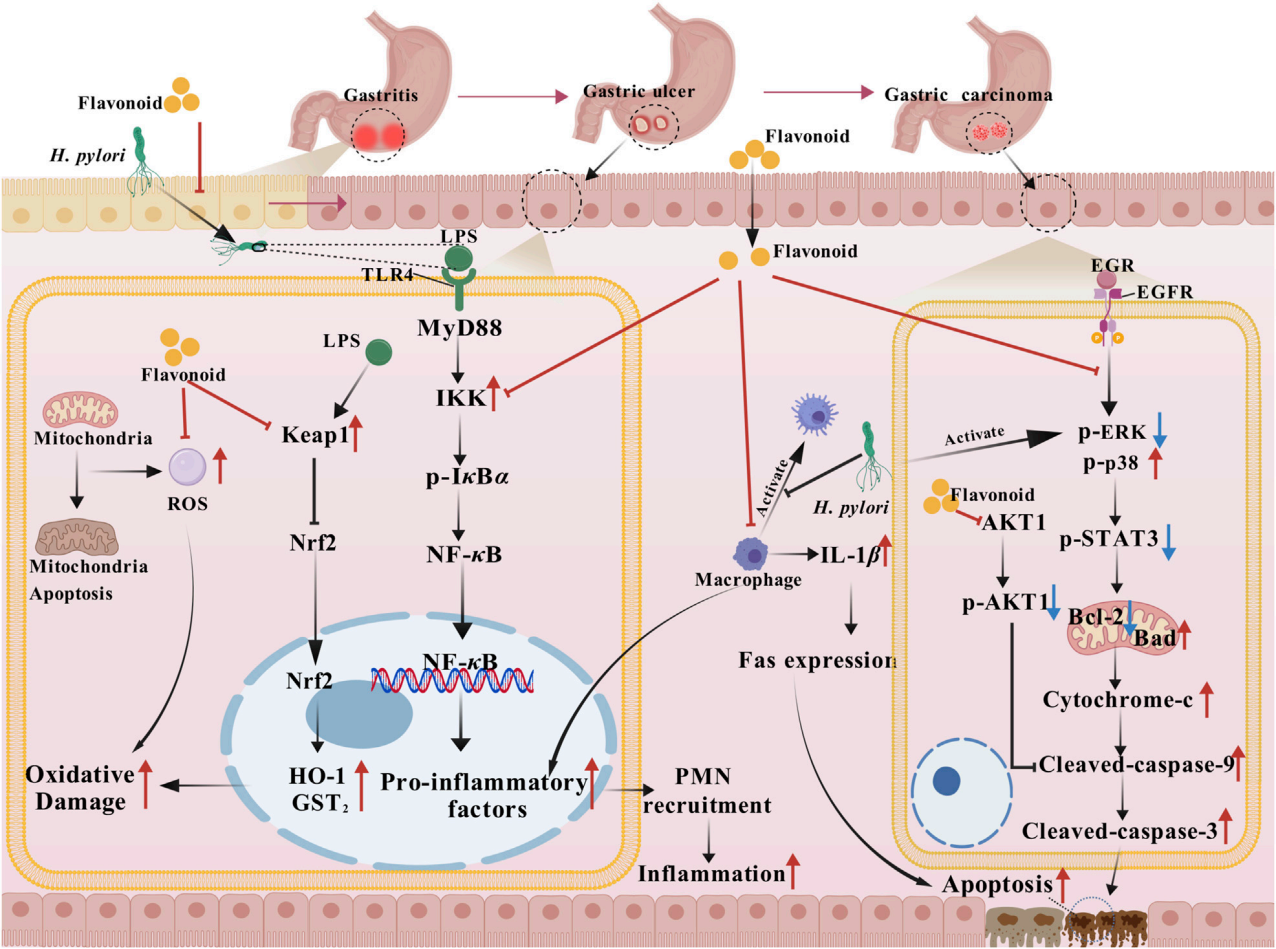
The main mechanism of action of flavonoids on gastric diseases. Flavonoids block the initiation of gastritis by inhibiting the binding of LPS derived from *Helicobacter pylori* to TLR4, thereby preventing MyD88 recruitment and IKK kinase activation. This reduces the phosphorylation and ubiquitin-mediated degradation of I*κ*B*α*, ultimately inhibiting NF-*κ*B nuclear translocation and the secretion of proinflammatory factors. Additionally, flavonoids suppress IL-1*β* release from macrophages and PMN recruitment. Second, flavonoids activate the Keap1/Nrf2 pathway, promoting Nrf2 binding to ARE to upregulate antioxidant enzymes like HO-1 and GST. Simultaneously, they maintain mitochondrial homeostasis to reduce ROS production, thereby scavenging excess ROS, mitigating oxidative damage, and feedback-inhibiting NF-*κ*B-driven inflammation, enhancing synergistic anti-inflammatory and antioxidant effects. Furthermore, flavonoids inhibit EGFR phosphorylation and downstream ERK/p38/STAT3/Akt1 pathway activation, restoring the Bcl-2/Bad balance. This suppresses mitochondrial cytochrome c release and caspase cascade activation, reducing excessive apoptosis in gastric epithelial cells and delaying the progression of gastritis to gastric ulceration. For lesions progressing toward gastric cancer, flavonoids continuously integrate the aforementioned anti-inflammatory, antioxidant, and anti-apoptotic mechanisms to block the vicious cycle mediated by *Helicobacter pylori-from* inflammation to oxidative stress to apoptosis-thereby inhibiting malignant progression of gastric mucosal lesions and exerting comprehensive protective effects throughout the disease course. Abbreviations. EGR, early growth response; GST2, glutathione S transferase theta 2; PNM, polymorphonuclear leukocytes.

The stomach, a key digestive organ, is susceptible to a spectrum of disorders termed gastric diseases. These disorders of the stomach, an essential digestive organ in humans, can interfere with normal digestion and hinder nutrient uptake, and might even endanger general health severely (as shown in Figure 3).

Gastritis is defined as gastric mucosal inflammation induced by multiple etiological factors. In terms of etiology, *Helicobacter pylori* colonization is a primary driver of gastric inflammation. Furthermore, factors like drug stimulation, alcohol and auto-immunity also have a part to play. Both eupatilin and vitexin belong to flavone compounds. Eupatilin can prevent the cytotoxin-associated gene A of *H. pylori* from moving to AGS human gastric cancer cells, and reverse the cell elongation (hummingbird cells) caused by cytotoxin-associated gene A, so as to decrease the infiltration of neutrophils and lymphocytes. Also, eupatilin can guard against gastritis. It does this by lessening the CagA/PI3K/NF-*κ*B-related inflammatory pathway and curbing the expression of pro-inflammatory mediators and monocyte chemotactic protein-1 (Lee et al., 2024). Additionally, treatment with vitexin is also able to cut down on pro-inflammatory cytokine generation and alleviate the triggering of NOD-like receptor thermal protein domain associated protein 3 (NLRP3) inflammasome. Furthermore, it can remarkably relieve the symptoms of rats with 1-methyl-3-nitro-1-nitrosoguanidine-triggered chronic atrophic gastritis by suppressing weight loss and decreasing the damage to gastric tissue (Liu J. et al., 2025).

Gastric ulcer is a chronic ulcerative lesion of the gastric mucosa caused by the digestive effects of gastric acid and pepsin. Globally, its incidence keeps on increasing, which makes it one of the typical digestive system diseases and a significant factor leading to serious outcomes like abdominal pain and gastric hemorrhage. For gastric ulcers, pharmacotherapy is the main clinical treatment. However, due to the increasing *H. pylori* resistance gradually and the individual variances in the regulatory mechanism of gastric acid secretion, the current treatments have certain limitations. Apigenin, which belongs to the flavone family of compounds, might offer an alternative approach in the context of gastric ulcers. In the rat model with indomethacin-induced gastric ulcer, treatment with apigenin safeguards the gastric mucosa through enhancing the activity of SOD and catalase, thus decreasing oxidative stress-mediated harm to gastric mucosal cells. The immunoreactivity of inflammatory markers like cyclooxygenase-2, TNF-*α*, and NF-*κ*B can be inhibited, which relieves the inflammatory harm to the gastric mucosa. Moreover, apigenin lessens cell apoptosis through reducing the transcription of Bcl-2-associated X (Bax) and raising that of B-cell lymphoma-2 (Bcl-2). It also encourages the expansion and differentiation of stomach mucosal cells by boosting TGF-*β* levels, which is conducive to the repair and regeneration of the gastric mucosa (Alamri, 2024). Genistein belongs to an isoflavone compound. Genistein can cause a significant decrease in the expression of the Wnt/*β*-catenin/TGF-*β*/Sma and Mad-related protein 4 signaling pathways. So it lessens the synthesis of extracellular matrix and relieves gastric tissue fibrosis. At the same time, genistein can reduce AKT expression and has an indirect influence on the repair of gastric tissue and inflammatory responses through the regulation of cellular physiological activities, so it has a positive effect on gastric ulcers (Hassan et al., 2023).

A malignant tumor that starts from the gastric mucosal epithelial cells, gastric carcinoma, has a seriously increasing incidence all over the world. Among the common malignant tumors in the digestive system, it can lead to serious outcomes like abdominal pain, weight loss, and gastrointestinal bleeding, and may even pose a threat to life. The main clinical treatments for cancer in the stomach are surgical operation, chemical therapy and radiation therapy. Due to tumor cell heterogeneity, the intricacy of the tumor microenvironment, and individual variations in patient treatment responses, existing therapeutic approaches have constraints. The flavonoid compounds include zapotin, pectolinarigenin and chrysin. The SNU-1 gastric cancer cell line can be made to undergo apoptosis by zapotin. This is achieved through the activation of intracellular mitochondrial apoptotic pathways or death receptor pathways, and also by elevating the expression and activity of pro-apoptotic proteins. Both zapotin and pectolinarigenin exert effects by hindering gastric cancer cell proliferation and inducing apoptotic cell death. This effect is achieved by downregulating the mechanistic target of rapamycin (m-TOR)/PI3K/AKT signaling pathway, suppressing the expression of its key proteins, and reducing the phosphorylation levels of downstream proteins such as phosphorylated eukaryotic translation initiation factor 4E-binding protein 1 (p-4EBP1), phosphorylated 70 kDa ribosomal protein S6 kinase (p-p70S6K), and phosphorylated eukaryotic translation initiation factor 4E. Moreover, pectolinarigenin suppresses the expression of X-linked inhibitor of apoptosis, an apoptosis inhibitor protein family member. This induces caspase-3 activation and further splits poly-ADP-ribose polymerase, thus starting an apoptotic cascade and causing cell apoptosis (Chen Y. et al., 2022; Lee et al., 2018). Chrysin can inhibit gastric cancer by reducing the expression of microRNAs (miR)-18, miR-21, and miR-221 and increasing the expression of let-7a, miR-9, miR-22, miR-34a, and miR-126 (Mohammadian et al., 2017). Naringin is a flavanone compound. In AGS cells, naringin treatment can lead to the generation of vacuoles in the cytoplasm and autophagosomes. Simultaneously, it activates autophagy-associated proteins including beclin 1 and microtubule-associated protein 1 light chain 3B, with this process further inducing cellular autophagy. In addition, naringin can enhance the expression of p21 cyclin-dependent kinase inhibitor 1/wild-type p53-activated fragment 1 protein, which is able to restrain CDK activity. As a result, this leads to cell cycle arrest and reduces the proliferative capacity of gastric cancer cells (Raha et al., 2015).

### 2.3 Liver diseases

The liver is the largest digestive gland in the human body, with core functions including the metabolism of the three major nutrients, detoxification, and bile secretion to assist in fat digestion (Alamri, 2018). The digested products of chyme enter the liver *via* the portal vein for metabolism, and abnormalities in esophageal or gastric function or imbalances in intestinal flora can indirectly affect the liver (Schneider and Trautwein, 2020). It functions integrally with the biliary tract system-biliary obstruction is prone to causing liver damage. It collaborates with the pancreas in digestion, and pancreatic abnormalities increase the liver’s metabolic load. Additionally, it is closely associated with the intestines through the “gut-liver axis” mutually influencing nutrient metabolism and the occurrence of diseases (He et al., 2024; Jain et al., 2024; Byndloss et al., 2024).

**FIGURE 4:**
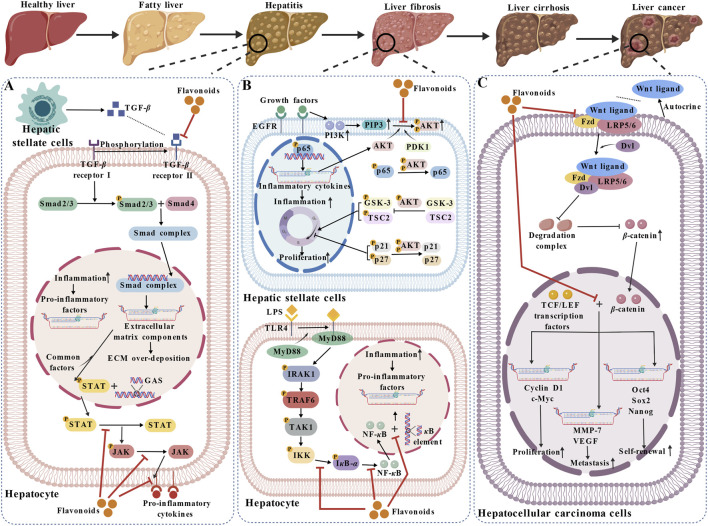
The role of flavonoids in the liver. **(A)** Flavonoids inhibit the binding of TGF-*β* produced by hepatic stellate cells to its receptors, block the phosphorylation of Smad2/3 and the formation of Smad complexes, and reduce the excessive synthesis and deposition of extracellular matrix (ECM) components. They also suppress the activation of the JAK/STAT pathway, thereby alleviating inflammation. Ultimately, this halts the progression of hepatic fibrosis. **(B)** In hepatic stellate cells, flavonoids inhibit the activation of the PI3K/AKT pathway, thereby reducing the proliferation and inflammatory response of these cells. In hepatocytes, they inhibit the NF-*κ*B pathway to decrease the production of pro-inflammatory cytokines, mitigate the inflammatory response, and delay the progression of liver cirrhosis. **(C)** In the treatment of liver cancer, flavonoids inhibit the binding of Wnt ligands to their receptors, block the Wnt/*β*-catenin signaling pathway, and thus suppress the proliferation, metastasis, and self-renewal of liver cancer cells, exerting an anti-cancer effect. Abbreviations: Dvl, dishevelled segment polarity protein; IRAK1, interleukin-1 receptor-associated kinase 1; LEF, lymphoid enhancer-binding factor; LRP5/6, low density lipoprotein receptor-related protein 5/6; Oct4, octamer-binding transcription factor 4; Sox2, SRY-related HMG-box 2; TAK1, transforming growth factor-*β*-activated kinase 1.

The liver is a vital metabolic organ responsible for detoxification, protein synthesis, and nutrient storage. Unhealthy diets, infections, and immune abnormalities are the primary etiological factors of liver diseases, which are typically manifested as fatigue, anorexia, jaundice, and right upper quadrant discomfort. Because of the liver’s great compensatory ability, initial symptoms are frequently faint and are readily overlooked; in the event that the disease advances, it can influence systemic metabolism and even pose a threat to health. It is essential to pay attention to the liver’s condition on a daily basis and keep its function in good condition by means of health management and timely intervention (as shown in Figure 4).

#### 2.3.1 Viral hepatitis

Viral hepatitis A, also known simply as hepatitis A, is an infectious disease brought about by Hepatitis A virus. It mainly features liver inflammation and is one of the leading causes of death globally (Usuda et al., 2024). Fatigue, loss of appetite, enlarged liver and abnormal liver function are among its clinical manifestations, and it is mainly transmitted *via* the fecal-oral route. Commonly, there are asymptomatic infections, and some cases show jaundice, mostly in the form of acute hepatitis. *Duck* hepatitis A virus serotype 1 (DHAV-1) has the ability to result in acute inflammatory injury in ducks, and its mortality rate is extremely high. This causes serious economic losses all over the world. Consequently, new ways of prevention and control for DHAV-1 infections need to be explored urgently. A flavonol compound named icariin, which is extracted from *Epimedium*, is able to decrease the mortality rate of ducklings that are infected with DHAV-1. After icariin is phosphorylated and modified, the phosphorylated derivative (pICA) exhibits a more significant inhibitory effect on DHAV-1-induced duckling mortality. Both icariin and pICA can stabilize mitochondrial function, and thus inhibit the harmful effects of DHAV-1 on apoptosis and cell cycle progression in cells. They can relieve inflammatory responses and protect duck embryo hepatocytes, with pICA having a stronger effect compared to icariin (Xiong et al., 2021). Furthermore, DHAV-1 infection induces mitochondrial membrane potential dissipation, lipid peroxidation, and reductions in antioxidant enzyme activity and mitochondrial respiratory chain complex activity. Baicalin, a flavone compound, can alleviate these changes and upregulate the gene and protein expression of nuclear factor-erythroid 2-related factor 2 (Nrf2). In turn, this leads to the activation of gene expression dependent on the antioxidant response element (ARE), such as heme oxygenase-1 (HO-1), nicotinamide adenine dinucleotide phosphate quinone oxidoreductase 1 (NQO1), SOD-1, and glutathione peroxidase (GPX)-1. It safeguards the model liver from the mitochondrial oxidative stress damage brought about by DHAV-1 (Su et al., 2021).

Hepatitis B virus (HBV) is a prevalent etiological factor for hepatitis globally. Clinical trials have been carried out on a variety of drugs against HBV, such as interferons and nucleotide analogs; yet, the effectiveness of these therapies is restricted. Therefore, it is of great importance to search for new anti-HBV drugs. A chalcone compound, 3,5,6,7,3′,4′-hexamethoxyflavone, has been under study because of its high inhibitory activity against hepatitis B surface antigen (HBsAg) and minimal cytotoxic effects. In both *in vitro* systems (HepG2.2.15 and HepG2-NTCP cells) and *in vivo* models (primary human hepatocytes and HBV transgenic mice), administration of 3,5,6,7,3′,4′-hexamethoxyflavone leads to a notable reduction in HBV RNAs, HBV DNA, and HBsAg. Furthermore, it attenuates the activity of four HBV promoters and lowers the ratios of total RNA to covalently closed circle DNA (cccDNA) and 3.5 kb RNA to cccDNA, thereby curbing HBV transcription (Tan et al., 2021). Amentoflavone, which is a bisflavonoid compound, decreases the RNA levels of all HBV biomarkers in HBV-infected cells, being a potential new anti-HBV drug (Aoki-Utsubo et al., 2023). As a flavone derivative, baicalin globally suppresses HBV replication and the secretion of viral antigens in HepG2.2.15 cells and HBV plasmid-transfected HepG2 cells. It is capable of efficiently reversing HBV resistance and achieving a synergistic curative effect *via* the downregulation of the hepatocyte nuclear factor (HNF) 4*α*-HNF 1*α* axis. In HepG2 cells, the heterodimerization of estrogen receptor *α*-HNF 4*α* can be promoted by baicalin, which inhibits cccDNA transcription in HBV (Niu et al., 2025). Dihydromyricetin belongs to the flavanonol compound. It can remarkably restrain both HBV replication and the secretion levels of HBsAg and HBeAg. Concurrently, dihydromyricetin reduces the mRNA levels of HBV RNAs in HepG2.2.15 cells. Furthermore, it can cut down the mRNA level of HNF 4*α* and also impede HBV replication through activating the NF-*κ*B pathway and MAPK signaling in addition to autophagy (Wang X. et al., 2023).

Liver disease around the world is often caused by *Hepatitis C virus* (HCV) infection. In various regions, different HCV genotypes have been found. Effective anti-HCV drugs are available, but they are prohibitively expensive for infected individuals in resource-constrained countries. It is necessary to develop drugs that target HCV with better cost-effectiveness. Silymarin, which is a type of flavanonol compound, can lead to a decrease in the levels of markers related to liver function, such as alanine aminotransferase, alkaline phosphatase, aspartate aminotransferase, and bilirubin. The levels of antioxidant markers (antioxidant and glutathione) are increased, those of oxidative stress markers (oxidized glutathione and malondialdehyde) are decreased, and the latent viral load is reduced by it, which helps in alleviating hepatitis C (Ahmed et al., 2022).

#### 2.3.2 Alcoholic liver disease

Chronic or excessive alcohol consumption induces a liver disorder termed alcohol-associated liver disease (ALD). Fatty liver is the main form that ALD initially takes, and it will develop into alcoholic hepatitis as the condition deteriorates. Flavonoids are more and more acknowledged for their powerful antioxidant capabilities and prospective curative function in handling ALD. Quercetin, kaempferol, and nicotiflorin belong to flavonol compounds. Excessive iron accumulation in the body is a hallmark feature of alcoholic liver disease. In the mouse model of ALD established with the Lieber-De Carli liquid diet, ethanol can lead to iron overload in hepatocyte lysosomes, which will damage the utilization of iron in cells and disrupt the normal iron metabolism process. Quercetin can upregulate the Rab7-V1G1 (V-ATPase subunit) axis to reverse iron overload, enhance lysosomal and mitochondrial function, thus decreasing ethanol-induced hepatocyte damage and preventing ALD (Lin et al., 2025). In mice with alcohol-related liver injury, kaempferol and nicotiflorin reduce hepatic oxidative stress markers and CYP2E1 levels, concurrently enhancing antioxidant enzyme activity. Kaempferol and nicotiflorin can boost the level of a certain enzyme related to silencing information regulator 2 by inhibiting the expression of miR-138-5p. This culminates in a reduction in farnesoid X receptor acetylation and then an induction of the Nrf2 and sterol regulatory element-binding protein-1c signaling pathways. Through this mechanism, they can modulate alcohol-induced oxidative stress and lipid metabolic processes, thus relieving liver injury (Ge et al., 2023). Nobiletin belongs to the flavone compound family. Li X. et al. created an ALD model in brain and muscle ARNT-like 1 (Bmal1) and Bmal1 liver-specific knockout mice *via* chronic ethanol feeding combined with binge drinking. Nobiletin can make AKT phosphorylation happen, raise the levels of carbohydrate response element binding protein, acetyl coenzyme A carboxylase 1, and fatty acid synthase, and decrease sterol-regulatory element binding protein 1 abundance and acetyl coenzyme A carboxylase 1 phosphorylation, thus relieving ALD (Li et al., 2024a). Hydroxysafflor yellow A is a chalcone compound. In ethanol-induced ALD mice, it lessens liver tissue damage by decreasing the levels of liver function markers as well as oxidative stress markers. In the alcohol-caused HepG2 cell injury model, hydroxysafflor yellow A can raise high-density lipoprotein (HDL)-C levels, boost the activity of antioxidant markers, cut down the accumulation of reactive oxygen species (ROS) within cells. By downregulating the PI3K/AKT and STAT3/NF-*κ*B pathways, it has effects that counteract oxidative stress, dampen inflammation, and forestall apoptosis, which effectively holds back the development of ALD (Wang W. et al., 2024). Puerarin is an isoflavone compound. In ALD mice, it can decrease iron overload. It achieves this by suppressing the activation of the MAPK/extracellular signal-regulated kinase (ERK) signaling pathway and raising hepcidin expression (Li et al., 2024b). Cyanidin-3-O-glucoside is an anthocyanidin compound. It has a positive effect on the pathological structure and function of the liver in mice and can suppress the accumulation of lipids in the liver. Narirutin, a flavanone compound, can help with alcohol-induced liver problems in mice. For example, Cyanidin-3-O-glucoside can relieve alcohol-caused intestinal barrier damage in mice. It does this by decreasing the amount of harmful intestinal flora and their metabolites, which in turn improves alcohol-induced ALD (Zhu L. et al., 2024). Narirutin is a flavanone compound. Park KH and others utilized zebrafish larvae to explore the curative effect of narirutin on acute liver injury induced by ethanol. Narirutin can adjust the changes in gene expression associated with oxidative stress, adipogenesis and the unfolded protein response pathway. It can target MAPK14 to inhibit the p38 MAPK signaling pathway, which can prevent lipid formation. Also, by down-regulating MAPK14, it can relieve oxidative stress and inhibit endoplasmic reticulum stress-induced apoptosis, thus reducing liver injury (Park et al., 2023). Oligomeric proanthocyanidins are flavanonol compounds. They can reduce acute alcoholic liver injury by inhibiting the levels of molecules entangled in inflammatory cascades and suppressing cell apoptosis *via* blocking the ROS-mixed lineage kinase domain-like protein-cathepsin B-NLRP3 pathway (Jin et al., 2025).

#### 2.3.3 Autoimmune liver disease

Autoimmune hepatitis is a chronic autoimmune disorder targeting the liver and has a genetic predisposition. There is a genetic factor in this disease. Its incidence grows as age increases, reaching a peak at over 70 years old, and its prevalence is also rising (Shiffman, 2024). Vitexin belongs to the flavone type. It notably lessens the accumulation of inflammatory cells and CD4^+^ T cells within liver tissues and relieves the liver damage brought about by oxidative stress. Vitexin treatment also alleviated the elevated levels of pro-apoptotic Bax and proteolytically activated caspase-3 as well as the downregulation of Bcl-2 in the liver of mice with autoimmune hepatitis, and enhanced the phosphorylation of AMP-activated protein kinase (AMPK), AKT, and glycogen synthase kinase 3*β*, thereby improving liver injury in mice with exercise-associated hyponatremia. In addition, vitexin can inhibit D-galactosamine/lipopolysaccharide (LPS)-induced apoptosis and overexpression of inflammatory cytokines in hepatocytes (AML12 cells) (Zhang L. et al., 2022).

#### 2.3.4 Metabolic dysfunction-associated fatty liver disease

Metabolic dysfunction-associated fatty liver disease (MAFLD) has swiftly become the most prevalent chronic liver disease globally, with current estimations suggesting it affects as much as 38% of the world’s adult population (Targher et al., 2024). A series of liver diseases are included in MAFLD, commencing with simple steatosis, and in later stages, it can develop into metabolic dysfunction-associated steatohepatitis (MASH), fibrosis, cirrhosis, and HCC. The pathogenesis of MAFLD stems from abnormal hepatic lipid metabolism, characterized by increased lipogenesis and elevated uptake of free fatty acids by hepatocytes due to insulin resistance. Both rutin and isoquercitrin belong to flavonols. Rutin can improve lipid accumulation in HeLa cells treated with oleic acid. It alleviates lipid accumulation, inflammation, and oxidative stress in mice with diabetic MAFLD. Rutin has the ability to relieve intestinal dysbiosis and improve MAFLD through activating the AMPK/sterol regulatory element-binding protein-1 pathway (Liu Y. et al., 2024). Ma J. and colleagues created a MAFLD mouse model with a methionine-choline-deficient diet. It was discovered that isoquercitrin treatment mitigated MAFLD through lowering the level of heat shock protein 90 and S-phase kinase-associated protein 1 G-2 allele, and thus suppressing the activation of the NLRP3 inflammasome (Ma et al., 2023). 7-Hydroxyflavone alleviates fat accumulation, hepatic steatosis and oxidative stress in MAFLD mice induced by a high-fat diet, and improves abnormal glucose metabolism. It can prevent triglyceride from depositing in HepG2 cells by interacting with serine/threonine kinase 24, which offers a novel approach to prevent and treat MAFLD (Qi et al., 2024). Baicalein is able to relieve lipid droplet buildup and hepatic steatosis, and reduce the levels of proinflammatory cytokines and intestinal permeability. Furthermore, baicalein is able to raise the protein expression level of phosphorylated NF-*κ*B p65 and the ratio of phosphorylated NF-*κ*B p65 to total NF-*κ*B p65, while reducing inhibitor kappa B alpha (I*κ*B*α*) and peroxisome proliferator-activated receptor alpha, thus relieving MAFLD (Guo et al., 2023). Dihydromyricetin is a kind of flavanonol. Dihydromyricetin, which is a flavanonol, has the function of reducing the amounts of pro-inflammatory factors in serum and increasing the level of beneficial intestinal bacteria when used for treatment, so it can effectively stop the development of MAFLD (Kang et al., 2024). Hesperetin is a kind of flavanone. Treatment with hesperetin is able to markedly decrease the body weight and liver index in rats with MAFLD. It can also ameliorate hepatic steatosis, lipid metabolism problems, and mitochondrial malfunction. Additionally, a certain substance can enhance the expression of mitofusin-2 and optic atrophy 1, while restraining the expression of fission protein 1. Through this approach, it can stop the progression of MAFLD (Chen S. et al., 2025). Soy isoflavone is one of the isoflavones. The levels of serum indicators related to lipid metabolism and hip circumference can be significantly decreased by it, and the metabolic status of MAFLD patients can be improved (Neshatbini Tehrani et al., 2024).

MAFLD has an inflammatory subtype known as MASH. There is hepatic steatosis in this subtype, and there is also evidence of hepatocellular injury (ballooning) and inflammation. Liver fibrosis may or may not be present. As time passes, MASH might develop into cirrhosis, terminal-stage liver disease, or need a liver transplant. Flavonoids can also have a curative impact on MASH. Flavonol kaempferol holds promise as a therapeutic agent for MASH. In the high-fat diet-induced mouse model of MASH and the palmitic acid/oleic acid-induced HepG2 cell model that simulates MASH conditions, kaempferol treatment can significantly reduce the expression of NLRP3-ASC/TMS1-caspase-3, thereby treating MASH (Yang H. et al., 2024). Breviscapine is a flavone. Lan T. and others respectively created MASH mouse models by administering a high-fat diet, a high-fat/high-cholesterol diet, or a methionine and choline-deficient diet, after which breviscapine treatment was carried out. In mice, breviscapine is able to markedly decrease lipid build-up in hepatocytes, infiltration of inflammatory cells, liver damage, and liver fibrosis. It can directly stop TGF-*β*-activated kinase 1 from being phosphorylated and halt the MAPK signaling cascade, thus preventing the development of metabolic stress-induced MASH (Lan et al., 2022).

#### 2.3.5 Chronic progressive liver disease

Liver fibrosis represents a prevalent public health concern. Liver fibrosis patients are more prone to have cirrhosis or HCC as more serious outcomes. Procyanidin B2 belongs to flavan-3-ols. In the CCl_4_-induced mouse liver fibrosis model and hepatic stellate cells, procyanidin B2 may result in the reduction of vascular endothelial growth factor A expression, HIF-1*α*, alpha-smooth muscle actin, collagen I and TGF-*β* in hepatic stellate cells, thereby inhibiting cell proliferation and triggering apoptosis. Furthermore, procyanidin B2 is able to impede the advancement of liver fibrosis by restraining the activation of hepatic stellate cells, the generation of extracellular matrix, and angiogenesis *via* the suppression of the Hedgehog pathway (Feng et al., 2019). Flavokawain A is a chalcone. In TGF-*β*1-stimulated vascular smooth muscle cells, flavokawain A can reduce the expression levels of matrix metalloproteinase (MMP)-9 and MMP-2, while enhancing the expression of tissue inhibitor of metalloproteinase 1. The activation of p-sma and mad-related protein 3 as well as wound healing migration are significantly decreased. Flavokawain is able to suppress fibrosis through the activation of the Nrf2/ARE signaling transduction for the restraint of ROS production (Hseu et al., 2019). Hesperidin, which is a flavanone, has the ability to counteract thioacetamide-induced liver fibrosis through downregulating the TGF-*β*/*α*-smooth muscle actin pathway, and it can also reduce the gene expression of cluster of differentiation 34 and fibroblast growth factor 23 (Megahed et al., 2024). Eupatilinis a flavone. The multiplication of LX-2 cells can be repressed through the downregulation of the activity and expression of cellular myelocytomatosis oncogene (c-Myc), cyclin B1, cyclin D1 and CDK6. Moreover, eupatilin can decrease the amounts of fibrosis markers like collagen type I alpha 1 chain and *α*-smooth muscle actin, along with epithelial-mesenchymal transition marker N-cadherin *via* suppressing the *β*-catenin/plasminogen activator inhibitor-1 signaling pathway. This action can prevent hepatic stellate cells from being activated and thus improve liver fibrosis effectively (Hu et al., 2023).

There are multiple factors around the world that can lead to cirrhosis, including obesity, MAFLD, excessive alcohol intake, hepatitis B or C infection, autoimmune disorders, cholestatic diseases, as well as iron or copper overloading. Following prolonged inflammation, cirrhotic transformations take place: healthy liver tissue gets substituted with fibrotic tissue and regenerative nodules, a process that in turn results in portal hypertension. A kind of flavanonol is silymarin. It can protect experimental animals from various hepatotoxic substances. A double-blind, prospective, randomized study involving 170 cirrhotic patients was carried out by Ferenci P. et al. to investigate the influence of silymarin on their prognosis. Ferenci P. et al. carried out a double-blind, prospective, randomized study on 170 cirrhotic patients to find out the effect of silymarin on the prognosis of these patients. The results demonstrated that silymarin treatment could reduce the mortality rate of cirrhotic patients, especially those with alcoholic cirrhosis, without side effects (Ferenci et al., 1989).

HCC ranks among the world’s most lethal cancers and also counts as one of the most prevalent malignant tumors. The incidence of HCC is on a continuous increase, and it constitutes a significant danger to human health (Wang H. et al., 2024). In the present HCC clinical treatment, most drugs are chemical substances. It has notable side effects and is liable to develop chemoresistance. As a result, developing natural compounds for HCC treatment has emerged as a new therapeutic approach. Many research findings have indicated that flavonoids have remarkable effectiveness in *in vivo* experimental research and show a powerful ability to inhibit tumor growth.

A malignant tumor that originates from liver epithelium is primary hepatic carcinoma, which accounts for around 50% of liver cancer patients globally. The incidence of primary hepatic carcinoma can be increased by hepatitis B, hepatitis C, dietary aflatoxin intake, and the influence of alcohol and other chemical substances (Han et al., 2023). Flavones include both morusin and oroxylin A. In the Hep3B mouse xenograft model, morusin has the ability to remarkably restrain the expression and activity of adenosine triphosphate (ATP) citrate lyase within HCC cells, resulting in the accumulation of ROS. Subsequently, it can trigger Phosphatase and tensin homolog (PTEN)-induced kinase 1/Parkin-mediated mitophagy and finally result in mitochondrial apoptosis. Morusin is also able to remarkably restrain the growth of tumor xenografts *in vivo*, which makes it a potential agent for HCC treatment (Li D. et al., 2024). Oroxylin A can activate rho-associated coiled-coil containing protein kinase 1 in HCC cells *via* caspase-3 mediation, which then causes the transfer of glycolytic proteins in HCC cell apoptosis-related extracellular vesicles to macrophages. This activates the ROS-dependent NLRP3 inflammasome, encourages the M1-like polarization of macrophages, and decreases the quantity of M2-like macrophages. Oroxylin A enhances immune checkpoint inhibitor therapy in mice by promoting T cell infiltration in the tumor microenvironment, thereby inhibiting the development of HCC (Wang P. et al., 2023). Glabridin and calycosin-7-glucoside are both isoflavones. In the H22 cell-established tumor-bearing mouse model, glabridin treatment can restrain HCC by increasing the expression levels of dual specificity phosphatase 5 gene, ZFP36 ring finger protein, KLF transcription factor 10, and nuclear receptor subfamily 4 group A member 1, while decreasing the expression of RecQ mediated genome instability 2 (Wang F. et al., 2025). HCC cell lines Huh-7 and HepG2 cells can have their proliferative activity reduced and be induced to apoptosis by calycosin-7-glucoside. It is also able to restrain the development of HCC in HepG2 xenograft mice through suppressing the expression of thioredoxin-1 for the promotion of apoptotic protein expression. Furthermore, calycosin-7-glucoside is able to considerably boost ROS generation, lower mitochondrial membrane potential, enhance the expression of crucial cell-apoptosis-related molecules and trigger mitochondria-led cell apoptosis, thus showing anti-HCC activity (Wei et al., 2023). Quercetin belongs to the flavonol type. Quercetin has the effect of making the mitochondrial network in Huh7 and Hep3B cells more fused and elongated by significantly increasing the expression of genes related to mitochondrial fusion and decreasing the expression of genes related to fission. Quercetin has the ability to boost the expression of sirtuin (SIRT) 1, strengthen the expression of crucial mitophagy regulators such as PTEN-induced kinase 1 and Parkinson disease 2, and raise PTEN-induced kinase 1/parkin-dependent mitophagy, which shows its potential for HCC treatment (Chen, 2024). (R)-7,3′-dihydroxy-4′-methoxy-8-methylflavane (DHMMF) belongs to the flavanone type. DHMMF can lead to the upregulation of p21 expression by inducing DNA damage, and in this way, it restrains the multiplication of human HCC cells, like HepG2 and SK-HEP-1 cells, resulting in cell apoptosis and G2/M phase arrest. Moreover, DHMMF shows great anti-HCC effectiveness in both xenograft and orthotopic hepatic carcinoma mouse models (Tian et al., 2023).

Metastatic liver cancer, which is another name for secondary liver cancer, is the result of malignant tumors from other bodily sites metastasizing to the liver and forming tumors there. Frequently, the symptoms of secondary liver cancer are not severe, and the development of the disease is rather insidious. When the quantity of cancerous tumors is few and their size is not large, secondary liver cancer is typically mainly shown by symptoms induced by primary cancers in other organs. As the metastatic foci in the liver grow gradually, patients may also show symptoms like those of primary liver cancer, namely, weight loss, fatigue, pain in the liver area, masses in the liver area, and even ascites and jaundice. Santin belongs to the flavonol category, and cirsimaritin is a member of the flavone group. They can effectively decrease the viability, proliferative ability, and clonogenicity of HepG2 cells. Also, they can stimulate cell apoptosis through the activation of caspase-3, -7, -8, and -9, thus showing favorable anti-cancer activity (Szoka et al., 2021). Scutellarin is a flavone. Both baicalin and scutellarin can impede the epithelial-mesenchymal transition procedure through decreasing the JAK2/STAT3 signaling pathway, so they can lower the *in vitro* invasivity of HepG2 and MHCC97-H cells *via* reshaping the cytoskeleton (Liu K. et al., 2019). Icaritin belongs to flavonol. Icaritin can promote a certain signal-mediated mitophagy by upregulating a loop, which is helpful to restrain the growth, multiplication and movement of HCC cells (Luo et al., 2024). Flavokawain C is a kind of chalcone. It has a tendency to be more concentrated in the liver tissues of tumor-bearing mice with xenografts. Flavokawain C can bring about the inhibition of the growth and migration of HCC cells and induce their apoptosis through the triggering of a strong DNA damage response. Additionally, by binding to the ATP sites of FAK and PI3K, flavokawain C can prevent their phosphorylation. In this way, it can repress the FAK/PI3K/AKT signaling pathway in HCC cells and restrain tumor growth in mice (Wang R. et al., 2024).

#### 2.3.6 Other types of liver diseases

Acute liver failure is a distinct clinically significant syndrome which has a high fatality rate. It is marked by metabolic dysregulation, neurological problems and multiple-organ malfunction. Luteolin-7-O-rutinoside has the ability to raise the levels of antioxidant markers so as to cut down oxidative stress. Moreover, it can exert a significant therapeutic effect in LPS/D-galactosamine-triggered acute liver failure in mice through restraining the production of pro-inflammatory cytokines and holding back the PI3K/AKT/AMPK/NF-*κ*B pathway (Xiong et al., 2022). Naringin and hesperetin are flavanones. Administering naringin in advance can relieve acetaminophen-induced liver damage. This is achieved by improving the expression levels of antioxidant enzymes, inhibiting proinflammatory factor production, and activating the apoptotic pathway. It is worth noting that naringin might be an effective activator of cation transport regulator-like protein 2 (CHAC2), which is able to protect mice from N-acetyl-p-aminophenol-induced acute liver injury *via* CHAC2-mediated activation of the Nrf2 pathway (Zhai et al., 2022). Hesperetin can activate the AMPK/cyclic AMP response-element-binding protein pathway to increase the expression of silent mating type information regulation 2 homolog 1, and suppress the inflammatory factor expression, thus reducing liver injury and inflammatory cell infiltration, and further protecting mice with acute liver injury (Wang et al., 2020).

The deposition of stainable iron-containing hemosiderin in liver tissues is what hemosiderosis means. The massive destruction of red blood cells results in the decomposition of hemoglobin, which is the main cause. Hepatocytes are mainly the sites where hemosiderin gets deposited, and Kupffer cells also often have such pigment deposition, yet it is typically less serious in Kupffer cells compared to hepatocytes. When it comes to cases related to blood transfusion, the pigment deposition in Kupffer cells becomes more evident. A flavonol named quercetin exists. Mohammed G. M. et al. established a rat model of lead acetate-induced liver toxicity. The treatment with quercetin was able to enhance the pathological state of liver tissues and relieve hepatotoxicity through the inhibition of serum liver function markers and the reduction of the rank of total bilirubin, creatinine, uric acid, and urea (Mohammed et al., 2017).

Metronidazole is the main drug for treating *Entamoeba histolytica*, which can cause amoebic colitis and amoebic liver abscess, a common extraintestinal lesion, among the pathogenic protozoa that lead to diarrhea. Nevertheless, drugs of the nitroimidazole type may face potential problems of drug resistance. Also, some research has, in a controversial manner, proposed that these drugs might lead to mutagenesis and neurotoxicity. Consequently, there is a necessity for natural and secure substitute drugs, and diverse flavonoids have antioxidant and antibacterial qualities. Since the 90s of the 20th century, certain researchers have centered on the identification and purification of various flavonoid compounds which have amoebicidal impacts, for instance, (−)-epicatechin and kaempferol (Ma et al., 2018). The *E. histolytica*-induced hamster model of golden amoebic liver abscess could be protected by kaempferol, and compared with metronidazole, it had fewer side effects (Varela-Rodríguez et al., 2024). (−)-Epicatechin belongs to the flavan-3-ol type. In the hamster model of liver amoebiasis induced by *histolytica* trophozoites, (−)-epicatechin could impede the development of liver abscess by curbing the production of pro-inflammatory cytokines. This, in turn, lessened the number of amoebae and aided in liver repair (Velásquez-Torres et al., 2021).

### 2.4 Biliary tract diseases

The biliary tract system is a crucial part of the digestive system. Its core functions include collecting, storing, concentrating, and releasing bile produced by the liver to assist in fat digestion. Composed of intrahepatic bile ducts and other components, the gallbladder is responsible for storing and concentrating bile, which is then discharged into the duodenum after meals (Yin et al., 2024). It maintains the closest connection with the liver, and biliary obstruction can cause liver damage. It co-shares an opening into the duodenum with the pancreas, and obstruction of the common bile duct may trigger pancreatitis. It forms an enterohepatic circulation with the intestines, interacting mutually. It is indirectly associated with the stomach and has no direct connection with the esophagus, only biliary tract diseases may indirectly affect esophageal function (Yin et al., 2024; Morales-Galicia et al., 2025).

Biliary tract diseases encompass gallbladder disorders and bile duct diseases, with etiological factors including infection, cholelithiasis, neoplasms, congenital anomalies, and parasites. These diseases are digestive system ailments, and their common symptoms are right upper abdominal pain, jaundice and fever.

Primary biliary cirrhosis (PBC), which is a chronic autoimmune cholestatic disease, mainly impacts women in the 40–70 age range. According to recent epidemiological research, despite geographical differences and a change in the proportion of men and women affected by the disease, the global incidence is still on the rise (Tanaka et al., 2024). In research related to PBC, luteolin has demonstrated remarkable therapeutic efficacy in both the *γ*-interferon-based primary biliary cholangitis ARE-Del^−/−^mouse model and the high-fat diet-fed mouse model. In both types of small mouse models, luteolin can adjust immune responses through suppressing the highly activated major histocompatibility complex class I and class II antigen presentation pathways. In ARE-Del^−/−^mice, it can boost the metabolic processes of hepatic fatty acid oxidation and oxidative phosphorylation, thus enhancing their metabolic state. Moreover, in both models, luteolin considerably strengthens the signaling of peroxisome proliferator-activated receptors, thus having a curative effect on PBC (Bae et al., 2020).

Globally, a certain disease is a significant reason for illness. Around the world, almost 800 million individuals are at risk of this disease. It is the second most widespread infectious disease following malaria. Globally, it is thought that around 250 million individuals in 78 countries are affected by this illness, leading to roughly 280,000–500,000 deaths annually (LoVerde, 2024). Licochalcone B is part of chalcones. An extracellular enzyme of *Schistosoma mansoni*, namely ATP diphosphohydrolase, is located on the epidermis surface of *S. mansoni* and is regarded as a decisive target for novel drugs. Adult *S. mansoni* can be completely killed by Licochalcone B. Moreover, the tegument morphology of these worms will be changed, and the egg-laying and movement abilities of adult worms will be significantly decreased, while mammalian Vero cells will not be affected by it. The compound Licochalcone B is effective in the treatment of schistosomiasis as it can lower the amounts of *S. mansoni* ATP diphosphohydrolase and adenosine diphosphatase (Aleixo de Carvalho et al., 2015).

### 2.5 Pancreatic diseases

The pancreas is located behind the stomach and has two core functions: exocrine and endocrine. Its exocrine function involves secreting digestive enzymes that are transported to the duodenum *via* the pancreatic duct to aid in the digestion of nutrients. The endocrine function relies on islet cells to secrete hormones into the bloodstream, regulating blood glucose and metabolism (Jain et al., 2024). It maintains close connections with other digestive tract organs, it indirectly regulates secretion with the esophagus and stomach, cooperates with the liver in digestion, with diseases of the two organs interacting mutually; connects to the biliary tract, where biliary tract diseases may trigger pancreatitis; and interacts directly with the intestines, mutually regulating digestion and secretion (Jain et al., 2024; Crespo et al., 2025; Zhang T. et al., 2025).

**FIGURE 5:**
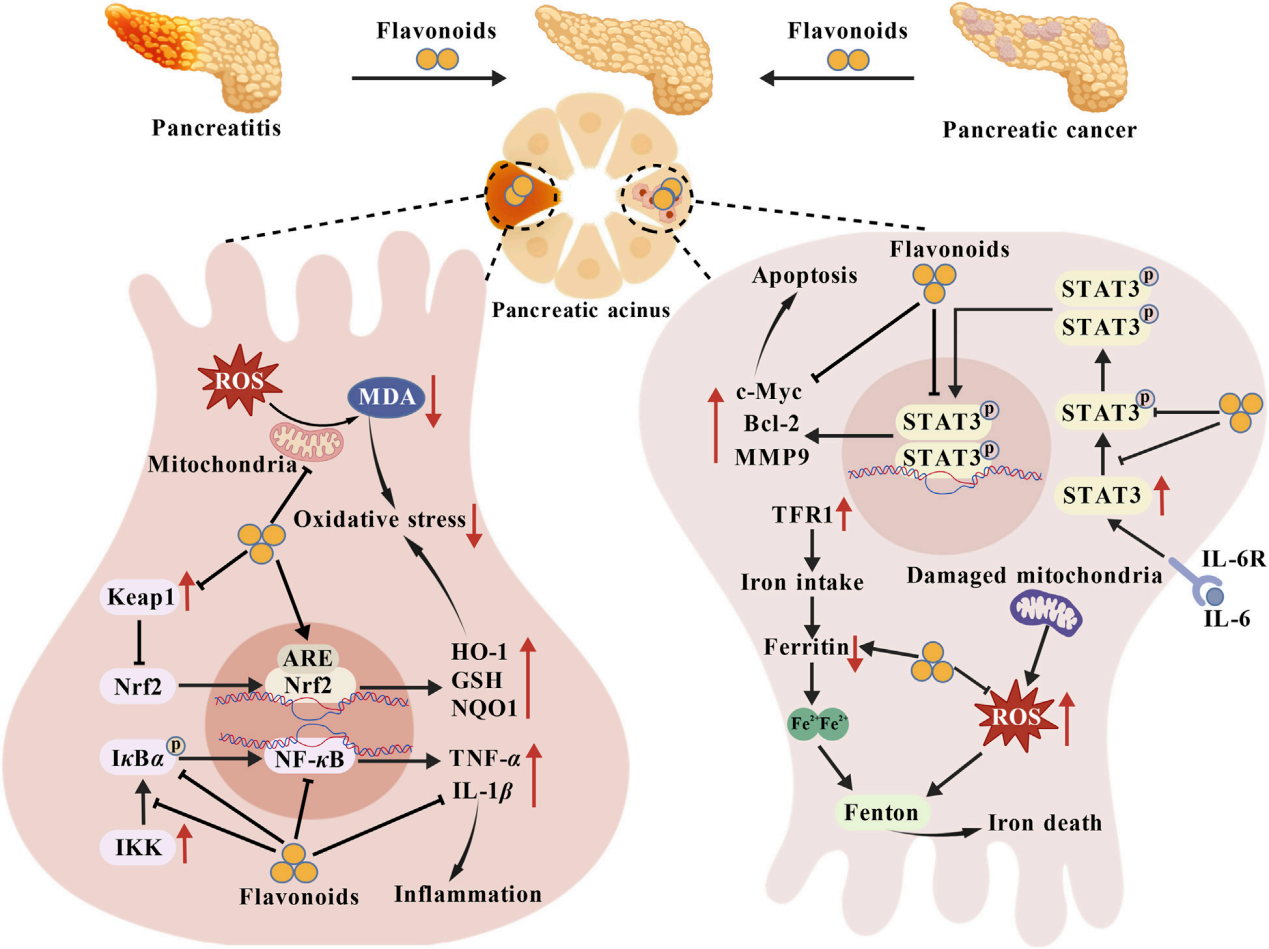
Therapeutic mechanism of flavonoids on pancreatitis and pancreatic cancer. The substantial ROS generated by oxidative stress not only directly causes cellular damage but also activates the IKK/NF-*κ*B inflammatory pathway, promoting the release of inflammatory mediators such as TNF-*α* and IL-1*β*. This inflammatory response further exacerbates oxidative stress, creating a vicious cycle. Flavonoids can reduce mitochondrial ROS production and lower MDA levels, directly interrupting the initiation phase of oxidative stress. Simultaneously, they activate the Nrf2 pathway, upregulating antioxidant enzymes such as HO-1, GSH, and NQO1, thereby enhancing cellular antioxidant capacity at its root. Concurrently, flavonoids inhibit IKK-mediated phosphorylation of I*κ*B*α*, thereby reducing NF-*κ*B activation and curbing inflammatory cytokine release. This reduction in cytokines subsequently diminishes their “amplifying effect” on oxidative stress. Flavonoids downregulate ferritin to lower intracellular Fe^2+^concentrations while simultaneously reducing ROS levels, thereby mitigating ferroptosis induced by the Fenton reaction between Fe^2+^ and ROS. As both a core mediator of oxidative stress and a key participant in ferroptosis, flavonoids establish a systematic, interconnected pharmacological network across diverse pancreatic pathologies by regulating ROS levels and multiple downstream pathways. Furthermore, flavonoids inhibit STAT3 phosphorylation and nuclear translocation, downregulate the expression of pro-cancer molecules such as c-Myc, Bcl-2, and MMP9, and induce tumour cell apoptosis. Abbreviations. ARE, Antioxidant Response Element; c-Myc, Myc Proto-Oncogene, BHLH Transcription Factor; Keap1, Kelch-like ECH Associated Protein 1; NQO1, NAD (P) H Quinone Dehydrogenase 1; STAT3, Signal Transducer and Activator of Transcription three.

The pancreas, a key digestive and endocrine organ, is susceptible to a spectrum of disorders termed pancreatic diseases. These disorders often cause symptoms such as abdominal pain, indigestion, and abnormal blood glucose levels. In serious situations, they are able to influence systemic metabolism and organ functions, which brings a great danger to patients’ wellbeing and lives (as shown in Figure 5).

Acute pancreatitis, a disease characterized by inflammation, mainly affects the pancreas. This disease is mainly caused by the activation of pancreatic enzymes in the pancreas. This activation can make the pancreatic tissue self-digest, and then it will cause a series of inflammatory responses like pancreatic congestion and edema. In severe cases, pancreatic necrosis can occur. Upper abdominal pain that is acute in onset, often intense and may spread to the lower back is among the clinical manifestations. Symptoms like nausea, vomiting and fever may occur as well. Flavones include tricetin and baicalein. Tricetin can lower expression of the pro-inflammatory cytokines, which helps to relieve the inflammatory response in caerulein-induced acute pancreatitis mice (Nagy-Pénzes et al., 2022). Baicalein is capable of reducing the expression levels of inflammation-promoting cytokines and boosting the expression level of miR-224-5p in AR42J cells. Baicalein can decrease the apoptosis of AR42J cells and relieve the damage to AR42J pancreatic acinar cells (PACs) caused by LPS through downregulating the Poly-ADP-ribose polymerase-1’s expression, NF-*κ*B65, p-I*κ*B*α*, IL-18R, gasdermin D, speck-like apoptosis-associated protein containing a CARD, NLRP3, and caspase-1 (Liu M. W. et al., 2024). Pinocembrin is a kind of flavanone compound. The pancreas can have its Nrf2 and HO-1 levels increased. And its antioxidant capacity can be enhanced by the triggering of the Nrf2/HO-1 pathway, which can relieve the oxidative stress damage in the pancreas. At the same time, pinocembrin is able to considerably decrease the expression of high-mobility group box 1 protein, toll-like receptor 4 (TLR4), NF-*κ*B, NLRP3 and pro-inflammatory cytokines in the pancreas so as to relieve inflammation. Moreover, pinocembrin can further restrain the NF-*κ*B pathway and boost anti-inflammatory impacts through decreasing the expression of miR-34a-5p and increasing the expression levels of peroxisome proliferator-activated receptor alpha, SIRT1 protein, and I*κ*B*α* gene. Pinocembrin is also able to modulate the ratio of Bax to Bcl-2 and prevent pancreatic cell apoptosis, which can greatly improve acute pancreatitis in rats (Ali et al., 2024). Isorhamnetin and galangin belong to flavonols. In the study on mice with severe acute pancreatitis triggered by sodium taurocholate, isorhamnetin is able to keep the mitochondrial membrane potential stable, guarantee the production of intracellular ATP, suppress the creation of mitochondrial ROS in pancreatic acinar cells, avoid oxidative damage and the release of mitochondrial DNA, and relieve oxidative damage and inflammatory reaction. At the same time, isorhamnetin inhibits the histone demethylase activity of lysine-specific demethylase 5B, which in turn increases the expression of pancreatic high-temperature requirement A2. This helps in maintaining normal mitochondrial function and reduces the harm of sodium taurocholate to PACs, thus relieving severe acute pancreatitis (Li et al., 2024d). Galangin can cut down the production of inflammation-promoting cytokines like monocyte chemotactic protein-1 and C-X-C motif chemokine ligand (CXCL10), along with ROS. It lessens the harm to tissue cells induced by inflammation and oxidative stress, and has a protective effect on severe acute pancreatitis in mice induced by L-arginine (Song et al., 2021). Tectoridin is an isoflavone. In mice with caerulein-induced severe acute pancreatitis, it is able to markedly decrease serum amylase and lipase levels, and relieve pancreatic injury. Tectoridin has the ability to make macrophages polarize towards the M2 type, lessen the inflammatory reaction, facilitate tissue repair and be used for the management of severe acute pancreatitis (Zhou et al., 2024). Dihydrokaempferol belongs to the flavanonol class. Oxidative stress marker levels in pancreatic tissue can be decreased by it, and the levels of antioxidant markers can be increased. Furthermore, it is able to lower the level of Kelch-like ECH-associated protein 1. At the same time, it enhances nuclear Nrf2 transcriptional activation. In this way, expression of antioxidant genes, the harm to pancreatic tissue brought by oxidative stress is alleviated, and the pancreatic damage caused by severe acute pancreatitis is improved (Liang et al., 2020).

A disease known as chronic pancreatitis may affect the pancreas, which is mainly marked by a chronic state of inflammation and the progressive development of fibrosis in the pancreas. The main cause of this condition is the long-term presence of various factors that keep harming pancreatic tissue, which in turn causes repeated or continuous inflammation of pancreatic parenchymal cells, and this results in unalterable changes in pancreatic structure and function, like atrophy of pancreatic acinar, stenosis or dilation of pancreatic ducts, and pancreatic calcification. A flavone named eruberin A exists. The triggering of pancreatic stellate cells serves a crucial early role in pancreatic fibrosis. In cells (LTC-14 cells with fundamental and morphological features of primary pancreatic stellate cells), eruberin A can effectively impede fibrotic mediator expression like *α*-smooth muscle actin, fibronectin, and type I-collagen when stimulated by TGF-*β*. This then suppresses component expression in the sonic hedgehog signaling pathway, which is beneficial for chronic pancreatitis treatment (Tsang et al., 2015). Quercetin is a type of flavonol. In mice with caerulein-induced chronic pancreatitis, the pancreas’ 5-hydroxytryptamine level can be significantly decreased by quercetin treatment. Also, pancreatic acinar-to-ductal metaplasia can be inhibited, and lesions like pancreatic intraepithelial neoplasia can be improved. Moreover, quercetin is able to notably reduce certain protein expression like ras homolog gene family, member A, rho-associated coiled-coil containing protein kinase 1, rho-associated coiled-coil containing protein kinase 2, *α*-smooth muscle actin, and NF-*κ*B, thus suppressing pancreatic tissue fibrosis in mice with chronic pancreatitis (Tao et al., 2020).

Pancreatic cancer is characterized by the uncontrolled proliferation of malignant cells in the pancreas. Pancreatic cells may develop genetic mutations due to prolonged exposure to various carcinogenic agents, which can lead to pancreatic cancer. These genetic alterations interfere with the regular control of cell growth, resulting in the unregulated division and multiplication of tumor cells. As a result of the abnormal cell growth regulation due to mutations, tumor cells divide and proliferate uncontrollably, and gradually, tumor tissues come into being, which are capable of infiltrating the surrounding tissues and metastasizing to remote areas. Fisetin and tiliroside belong to flavonol compounds. Fisetin is able to reduce the level of a certain transcription factor and boost the expression of a protein related to cell proliferation and growth. Fisetin lessens the nuclear build-up of *β*-catenin, thus restraining the Wnt/*β*-catenin signaling pathway and efficiently curbing the growth of pancreatic cancer (Fu et al., 2025). Tiliroside directly attaches to and restrains the activity of calcium-dependent cysteine protease-2/calpain-2. This activity deactivates AKT in pancreatic cancer cells, reduces the expression of glucose transporter (GLUT) 1 and GLUT3, undermines the production of ATP and nicotinamide adenine dinucleotide phosphate hydrogen, and gives rise to autophagy and ROS generation. The ROS that has been generated combines and accumulates with iron from the Labile Iron Pool, which then sets off the Fenton reaction and leads to lipid peroxidation. The decrease of free iron ions induced by tiliroside can promote the autophagic degradation of ferritin, which further intensifies ferroptosis in pancreatic cancer cells, restrains PANC-1 cell proliferation in xenograft mice, and is thus beneficial to the treatment of pancreatic cancer (Xu et al., 2024). Xanthohumol, a chalcone compound, in combination with plumbagin, can downregulate the gene level of Bcl-2 to trigger apoptosis in pancreatic cancer cells. This combination has significant synergistic anticancer effects on various pancreatic tumor cell lines *in vitro* when combined. This combination effectively restrains the proliferation of cancer cells and suppresses tumor development in KPC transgenic mice with pancreatic cancer (Palanisamy et al., 2024). Taxifolin is in the flavanonol category and also known as dihydroquercetin. By regulating the HIF signal pathway, it is able to promote the apoptosis in pancreatic cancer cells, and suppress their infiltration and metastatic spread (Chen et al., 2024).

### 2.6 Intestinal diseases

The intestine is the core organ of the digestive system, with core functions including food digestion and absorption, water-electrolyte balance, immune defense, and endocrine regulation. The small intestine increases its surface area through villi to absorb nutrients, while the large intestine focuses on water and electrolyte absorption, fecal storage, and maintenance of intestinal flora homeostasis (Yilmaz and Macpherson, 2024). It maintains close connections with various digestive organs, it regulates gastric emptying, forms an enterohepatic circulation with the liver, aids in digestion by utilizing bile from the biliary tract and digestive enzymes from the pancreas, and simultaneously regulates the secretion of related organs (Yilmaz and Macpherson, 2024; Uddin and Rumman, 2022; Sternini and Rozengurt, 2024).

**FIGURE 6:**
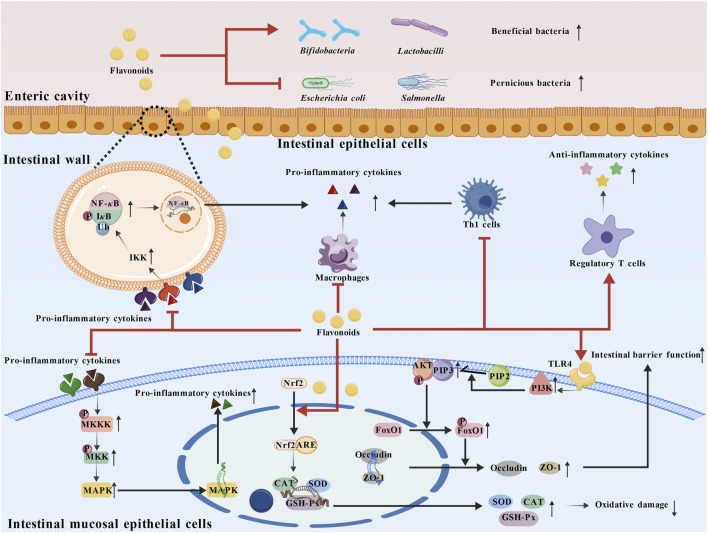
The mechanism of flavonoids in improving intestinal diseases. In the enteric cavity, flavonoids can promote the proliferation of beneficial bacteria while inhibiting the growth of harmful bacteria, thereby optimizing the intestinal flora structure. After the proliferation of beneficial bacteria, they can inhibit the activation of pro-inflammatory signaling pathways such as NF-*κ*B and MAPK through metabolites or direct interaction with intestinal epithelial cells, reducing the release of pro-inflammatory cytokines in the intestinal wall. Excessive proliferation of harmful bacteria, however, will exacerbate the activation of inflammatory signals and amplify the inflammatory response of the intestinal wall. Furthermore, in the intestinal wall, flavonoids can inhibit the activation of IKK kinase, reduce the phosphorylation and ubiquitination of I*κ*B, thereby preventing NF-*κ*B from entering the nucleus, ultimately inhibiting the release of pro-inflammatory cytokines and blocking the initiation of the inflammatory cascade. Meanwhile, they can inhibit the activation of the MAPK pathway mediated by pro-inflammatory cytokines, further reducing the release of pro-inflammatory cytokines and enhancing the anti-inflammatory effect. Flavonoids are capable of inhibiting the release of pro-inflammatory cytokines from macrophages and Th1 cells, and promoting the proliferation of regulatory T cells to upregulate the release of anti-inflammatory cytokines, maintaining immune balance. They can activate the Nrf2 pathway, enabling it to bind to ARE, promote the synthesis of antioxidant enzymes (SOD, CAT, GSH-Px), and effectively reduce intestinal oxidative damage. By regulating the PI3K/AKT pathway, flavonoids can promote the expression of tight junction proteins (e.g., Occludin, ZO-1) and enhance intestinal barrier function. Abbreviations: GSH-Px, glutathione peroxidase; FoxO1, fork head box protein O1; ZO-1, Zonula Occludens-1.

The intestines are vital for human digestion and absorption, and there are various disorders that can affect them, which are collectively known as intestinal diseases. There are various types among these diseases, like the inflammatory, neoplastic and functional ones, and they are likely to lead to manifestations including abdominal pain, diarrhea, constipation, and hematochezia. They can have an impact on the normal digestive, excretory and immune functions in the intestines, thus disturbing the overall nutritional metabolism and healthy balance in the body (as shown in Figure 6).

#### 2.6.1 Diseases of the small intestine

The initial part of the small intestine is the duodenum, which is also the shortest, widest, deepest and most fixed part of the small intestine. A chronic ulcerative condition known as duodenal ulcer affects the duodenal mucosa. The main factors contributing to this are *H. pylori* infection and excessive gastric acid production, which result in chronic ulcerative damage to the duodenal mucosa. Symptoms like dyspepsia may occur. According to the different degrees of manifestation, there could also be situations such as gastrointestinal bleeding, gastric outlet obstruction and perforation. Multiple factors are involved in its pathogenesis. These factors are a weakened gastric mucosal defense system, toxins from *H. pylori*, the mucosa’s self-digestion by gastric acid, and a great many inflammatory mediators and cytokines. An anthocyanidin is malvidin. It could increase the gene expression of EGF and COX-1, and downregulate MMP-9, thereby accelerating the healing of acetic acid-induced ulcers. Malvidin can relieve the influence of multi-drug treatment on intestinal inflammation and oxidative stress. Furthermore, malvidin could protect the mouse intestine by reducing TLR4 and increasing Heme oxygenase 1 and IL-10. Consequently, malvidin has remarkable anti-inflammatory and antioxidant functions in the gastrointestinal tract and is capable of preventing duodenal ulcers (Fagundes et al., 2021).

The absorptive function of the small intestine mainly takes place in its central part, the jejunum. The mucosal layer of it has villi, crypt structures and tight junctions, which form a barrier that is very important for nutrient absorption and protection. The most common lesion in the jejunum is the mucosal injury. Infection, stress, toxins or nutritional imbalance can cause it, and anti-inflammatory, antioxidant and barrier-repair measures are required for intervention. Dihydromyricetin is a type of flavonols. The antioxidant capacity of the jejunal mucosa was strengthened by its activation of Nrf2. Moreover, dihydromyricetin could cause the expression of TLR4, p-NF-*κ*B, and HIF-1*α* to be downregulated, and it can also prevent the NLRP3 inflammasome from being activated. As a result, it lessens jejunal mucosal inflammation and endoplasmic reticulum stress, makes jejunal mucosal permeability and morphological structure better, and further relieves LPS-induced jejunal mucosal injury in weaned piglets (Chen X. et al., 2025).

#### 2.6.2 Diseases of the large intestine

In the digestive system, the large intestine plays a crucial role. It mainly undertakes tasks like water absorption, feces storage and excretion. Symptoms like abdominal pain, bloating, bloody stools and changes in bowel habits are often shown in the diseases which affect the large intestine. Frequently, these diseases are related to mucosal injury, problems with function, infections, or structural irregularities, which could disrupt the digestive function. Based on symptoms, it is necessary to carry out timely examinations and interventions.

##### 2.6.2.1 Diseases of the colon

A chronic inflammatory bowel disease which is called Crohn’s disease is capable of influencing the whole digestive tract, with the terminal part of the small intestine being the most commonly involved area. Young people aged 15–35 have a high incidence of it. The incidence of Crohn’s disease varies by region and ethnicity, with the highest rates in North America and Northern Europe, while the incidence in Asia has been on the rise in recent years. The surgical treatment rate of Crohn’s disease is high, yet it is likely to recur. Although the inflammatory pathogenesis of Crohn’s disease has been extensively studied, research on the pathogenesis of intestinal fibrosis and strictures remains limited (Zhang et al., 2024). Prunetin is an isoflavone compound. In models of colonic inflammatory injury induced by LPS and the mouse colitis induced by 2,4,6-trinitrobenzene sulfonic acid, prunetin treatment inhibited the release of pro-inflammatory factors in colonic tissues, promoted the expression levels of zonula occludens-1 and claudin-1, and maintained the intestinal barrier structure. By counteracting TLR4/myeloid differentiation response gene 88 signaling, prunetin can restrain intestinal epithelial inflammation and keep the intestinal barrier from being damaged, which is beneficial for relieving Crohn’s disease-like colitis (Li J. et al., 2024).

The colonic mucosa may have protrusive lesions known as colonic polyps, which are very harmful. These protrusive lesions of the colonic mucosa may lead to intestinal manifestations like bloody stools, bellyache, looseness of the bowels, and blockage of the intestine, and there is also a potential for malignancy. Adenomatous polyps are a prime example; for instance, the malignancy rate of villous adenomas can be anywhere from 30% to 70%. When malignant transformation takes place, it is difficult to treat and can be life-threatening. Chafuroside is a flavone compound. Apc-deficient Min mice serve as an animal model of human familial adenomatous polyposis, developing numerous polyps in the intestine. Chafuroside can significantly inhibit the growth of colonic and small intestinal polyps in Min mice. Moreover, within a specific dose range, it is fairly safe, having little influence on the fundamental physiological functions, growth, and development of the mice (Niho et al., 2006).

Ulcerative colitis is a bowel disease with inflammation, showing relapsing and remitting characteristics. Mucosal inflammation that begins at the distal end can spread proximally and may even affect the whole colon, which is a characteristic of it. At present, the prevalence of this condition is increasing rapidly all over the world, mainly striking adults in the 30–40 age range. Ulcerative colitis can be severe and may develop into colitis-associated colorectal cancer, which endangers patients’ lives greatly. Morin is a type of flavonol compound. It could improve ulcerative colitis in mice which is induced by dextran sodium sulfate. It did this by suppressing inflammatory responses, strengthening intestinal barrier function, spurring the proliferation of beneficial bacteria in the microbiota including *Muribaculaceae* and *Erysipelotrichaceae*, and decreasing the quantity of harmful bacteria like *Erysipelotrichaceae* and *Firmicutes* (Qiu et al., 2024). Acacetin, genkwanin, licoflavone B, and tricin all fall into the flavone category. Take acacetin as an example. It can relieve certain clinical manifestations in mice with UC, for instance, weight loss, diarrhea, shortening of the colon, inflammatory cell infiltration, and histopathological damage. In addition, it can lower the quantity of *Proteobacteria* and *Deferribacteres*, raise the quantity of *Firmicutes*, and make the intestinal microbiota return to a state similar to that of normal mice, so as to regulate intestinal immune responses in an indirect way and ease intestinal inflammation. At the same time, acacetin can make LPS-induced bone marrow-derived macrophage cells secrete less NO and express less iNOS protein, which lessens macrophage infiltration and thus ameliorates ulcerative colitis (Ren et al., 2021). Genkwanin can relieve ulcerative colitis in mice through decreasing the levels of oxidative stress indicators, raising the levels of antioxidant indicators to relieve oxidative stress, and diminishing the synthesis of pro-inflammatory cytokines. *In vitro*, genkwanin can boost the expression of SIRT1, raise oxygen consumption rate and mtDNA content, and strengthen the activity of electron transfer chain complexes I, II, and IV, so as to relieve ulcerative colitis (Chen Z. et al., 2022). Licoflavone B could stop colonic cell apoptosis by blocking the MAPK signaling cascade, and increased the production of tight junction tight junction molecules to keep the integrity of the colonic barrier. Moreover, it modifies the equilibrium of the gut microbiota through suppressing the proliferation of pathogenic bacteria and stimulating the growth of beneficial bacteria, thereby ameliorating ulcerative colitis in mice (Zhang J. et al., 2022). Tricin alleviated colitis in mice through lowering the numbers of pathogenic bacteria like *Proteobacteria* and *Ruminococcaceae*. Moreover, in LPS-stimulated RAW264.7 cells, tricin could exert anti-inflammatory effect by suppressing the NF-*κ*B signaling pathway, thereby decreasing NO generation (Li et al., 2021).

The gastrointestinal tract often has a common malignant tumor known as colon cancer. Its incidence rate is rising, and it ranks as the third most prevalent cancer worldwide, as stated in (Abdelmaksoud et al., 2025). Unhealthy dietary habits, along with factors like smoking or decreased physical exercise, are mainly responsible for this disease. Particularly after metastasis, it results in a poor prognosis. Colorectal cancer often afflicts patients with long-term chronic inflammation in inflammatory bowel disease. As the present treatment choices are restricted, the mortality rate of colorectal cancer that cannot be surgically removed is extremely high. The flavone class includes cudraflavone C, apigenin and scutellarein. Cudraflavone C had anti-colorectal cancer impacts by suppressing GPX-2 expression, hindering the activation of GPX-2-dependent Wnt/*β*-catenin pathway, decreasing *β*-catenin’s stability and nuclear translocation and repressing downstream pro-cancer genes’ expression (Wu et al., 2024). In various colorectal cancer cell lines such as HCT-116, HT-29, SW-480, DLD-1, Caco2, SNU-C4, HCT-8, LS174T, COLO-205 and HCT-15, apigenin enhanced cleaved-caspase-3 expression, reduced mRNA expression of mitochondrial SIRT3 and SHMT2 triggered by SIRT3 and induced caspase-3-dependent apoptosis (Abdelmaksoud et al., 2024). Scutellarein is able to downregulate aquaporin 1, aquaporin 3 and aquaporin 5 expressions and upregulate the expression of phosphatase and tensin homolog deleted on chromosome 10, thus inhibiting the PI3K/AKT signaling pathway and achieving the suppression of colorectal cancer cell growth, migration and invasion (Tarawneh et al., 2024). Broussoflavonol F and isoorientin are both flavone compounds. Broussoflavonol F can lead to apoptosis in HCT-116 and LoVo cells, stop the cell cycle at G0/G1 phase and prevent cells from entering S phase for DNA synthesis, thereby inhibiting cell division and proliferation. It hinders the activation of the human epidermal growth factor receptor 2(HER2)-rat sarcoma viral oncogene homolog (RAS)-phosphorylated B-raf, p-mitogen-activated protein kinase kinase (MEK)-ERK signaling cascade through the downregulation of HER2, RAS, MEK and p-ERK. As a result, it can effectively hold back the proliferation of colon cancer cells. In the zebrafish embryo model, broussoflavonol F can play an anti-angiogenic role. It achieves this by decreasing the expression of angiogenesis-related genes and restraining the growth and development of subintestinal vessels. Furthermore, broussoflavonol F has the ability to impede the proliferation, movement, and tube-forming capacity of HMEC-1 cells. This directly prevents vascular endothelial cells from creating vessel-like structures, thereby suppressing tumor angiogenesis (Zhu Y. et al., 2024). Isoorientin serves as a new inhibitor for C-X-C chemokine receptor type 4 (CXCR4). The CXCR4 receptor’s expression is a key factor in the communication between malignant cells and their tumor microenvironment. In breast and colorectal cancer cells, isoorientin could impede NF-*κ*B activation, and this in turn can inhibit CXCR4 expression. This inhibition weakens the response of cancer cells to CXCL12 and undermines their invasive and metastatic capabilities (Kim and Park, 2024). Naringenin is a flavanone, while diosmin is a flavone compound. In colorectal cancer cell cultures HCT116 and SW480, the combination of naringenin and diosmin can effectively promote apoptosis by enhancing chromatin compaction, DNA cleavage, and inducing G0/G1 phase cell cycle arrest (Zeya et al., 2022).

A common gut-brain interaction disorder is irritable bowel syndrome (IBS), which is mainly due to visceral hypersensitivity and substantial intestinal microbiota dysbiosis, and it impacts 5%–10% of the global general population (Mayer et al., 2023). Cells rely on autophagy as a vital process to maintain internal balance and get rid of damaged organelles and proteins. In Caco-2 cells, exosomes derived from irritable bowel syndrome (IBS-exo) can aggravate IBS symptoms through suppressing autophagy. Apigenin has the ability to remove the autophagy-inhibiting effect of IBS-exo on Caco-2 cells, reinstate normal autophagy levels in the cells, and impede the development of IBS. This is accomplished through the upregulation of ATG14, which is a crucial protein for promoting autophagy, along with the reduction of the inhibitory impact of miR-148b-3p on autophagy-related genes (Fu et al., 2023). Both 5-demethylnobiletin and nobiletin belong to the flavone class of compounds. They can regulate biotransformation by inhibiting cytochrome P450 1B1 and upregulating detoxifying enzymes, thereby alleviating Benzo [a] pyrene-induced DNA damage and preventing the malignant transformation of IBS (Lin W. S. et al., 2023).

### 2.7 Pharmacological mechanisms of flavonoids in digestive diseases

Flavonoids can precisely block the pathological progression of digestive system diseases through multi-target synergistic effects. In the regulation of inflammation-oxidative stress, when neutrophils are activated by inflammatory stimuli such as LPS, they generate large amounts of superoxide (O_2_^−^) *via* NADPH oxidase. This O_2_^−^ further activates p38 MAPK through oxidative modification, accelerating I*κ*B-*α* degradation and enhancing NF-*κ*B p65 phosphorylation, ultimately inducing the production of pro-inflammatory factors such as TNF-*α* and IL-6. This establishes a vicious cycle of “inflammatory activation→O_2_^−^ release→p38/NF-*κ*B activation→inflammatory amplification” (Mitra and Abraham, 2006). Flavonoids exert anti-inflammatory and antioxidant effects through synergistic mechanisms by inhibiting NF-*κ*B phosphorylation and p38 MAPK activation. This dual action blocks the inflammatory cascade while reducing ROS production (Zhao et al., 2022; Hu et al., 2021). In the dysregulation of cell proliferation-apoptosis balance, digestive system tumours such as HCC and gastric carcinoma frequently exhibit dual abnormalities characterised by “activated pro-proliferative pathways coupled with inhibited apoptotic pathways”. Flavonoids can synergistically intervene in tumour proliferation through multi-target mechanisms. On the one hand, by downregulating p-PI3K and p-AKT expression to inhibit the PI3K/AKT/mTOR pathway, thereby directly arresting tumour cells in the G0/G1 phase and indirectly regulating ATP citrate lyase (ACLY) activity. Simultaneously, they upregulate the Bax/Bcl-2 ratio and activate cleaved-caspase-3/9, directly activating the mitochondrial apoptosis pathway. Concurrently, this flavonoid-mediated regulation of ACLY reduces acetyl-CoA production. This substance serves as both a core precursor for tumour stem cell lipids/cholesterol synthesis and an epigenetic modification substrate regulating cell proliferation. Ultimately, they exert antitumour effects through the synergistic formation of “cycle arrest+apoptosis activation+metabolic inhibition” (Lee et al., 2018; Li D. et al., 2024), demonstrating antiproliferative activity and proapoptotic effects in malignant diseases such as HCC and gastric cancer. Beyond these two pathological dysfunctions, multi-faceted imbalances mediated by the gut microbiota also constitute a significant pathogenesis mechanism for digestive disorders. Taking IBS as an example, the gut microbiome initially exhibits “structural imbalance,” progressively triggering multiple chain reactions including compromised intestinal barrier integrity, immune dysregulation, abnormal gut-brain signalling, and disrupted metabolic and neurotransmitter regulation. This ultimately leads to the onset and persistence of IBS symptoms. Quercetin mitigates intestinal inflammation at its source by reducing the abundance of *Bacteroides* species, thereby decreasing the number of Gram-negative bacteria in the gut and lowering LPS release. simultaneously activating the Nrf2/HO-1 pathway to enhance SOD and catalase activity. This removes accumulated ROS within the gut, thereby collectively interrupting the vicious cycle of “microbiome imbalance-inflammation-oxidative stress-barrier disruption”. This prevents ongoing damage to the intestinal mucosa and aids in improving IBS-related pathological states (Xia et al., 2025).

This synergistic effect does not occur in isolation but forms a causal chain through cross-mechanism regulation, wherein the activation of one mechanism creates favourable conditions for subsequent pathological interventions. Taking MAFLD as an example, its core triggers are gut microbiota dysbiosis and intestinal barrier disruption, allowing LPS to enter the liver *via* the portal vein and induce hepatic inflammation and lipid deposition. The flavonoid compound rutin can first modulate the gut microbiome by reducing the *Firmicutes*/*Bacteroidetes* ratio, thereby decreasing LPS production in the intestine and promoting the proliferation of beneficial bacteria such as *Muribaculaceae*. The short-chain fatty acids (SCFAs) produced by these beneficial bacteria can further upregulate the expression of intestinal tight junction proteins like occludin and ZO-1, thereby enhancing intestinal barrier integrity and blocking LPS entry into the liver at its source (Yan, 2022). SCFAs entering the liver can directly activate the AMPK signalling pathway, inhibiting the activity of sterol regulatory element-binding protein 1 (SREBP1), a key factor in lipid synthesis. This reduces triglyceride and cholesterol deposition within hepatocytes while concurrently lowering hepatic ROS levels. This ultimately establishes a chain-like regulatory mechanism: “modulation of gut microbiota→restoration of intestinal barrier function→improvement of hepatic metabolism and oxidative stress”, significantly alleviating lipid deposition and inflammation in diabetes-associated MAFLD (Li M. M. et al., 2022; Wu et al., 2020). In chronic inflammation-associated diseases such as oesophageal squamous cell carcinoma, where cellular homeostasis is disrupted, prolonged chronic inflammation readily induces sustained oxidative stress. This leads to DNA damage and mitochondrial dysfunction, ultimately disrupting the balance between cell proliferation and apoptosis, manifesting as uncontrolled proliferation and suppressed apoptosis. The flavonoid compound cirsiliol first exerts anti-inflammatory effects by targeting tyrosine kinase 2 (TYK2), thereby inhibiting the TYK2/STAT3 pathway. This reduces STAT3 nuclear translocation and downregulates the expression of pro-proliferative genes such as c-myc and pro-inflammatory genes like IL-6, simultaneously suppressing tumour cell proliferation and alleviating inflammatory responses. Concurrently, STAT3 pathway inhibition indirectly reduces ROS production, as STAT3 activation promotes NOX4-mediated ROS synthesis. Flavonoids directly scavenge mitochondrial ROS and stabilise mitochondrial membrane potential, mitigating oxidative stress-induced mitochondrial damage to preserve cellular structural integrity. Moreover, flavonoids restore cellular homeostasis by regulating proliferation and apoptosis. They arrest the cell cycle at the G2/M phase, inhibiting abnormal proliferation; while simultaneously downregulating the expression of the anti-apoptotic gene Mcl-1 to promote apoptosis in damaged cells. This establishes a cross-mechanism pathway: “anti-inflammation reduces oxidative sources→antioxidant protection safeguards cellular structure→regulation of proliferation and apoptosis restores homeostasis”. This pathway has demonstrated efficacy in both *in vitro* cellular models of oesophageal squamous cell carcinoma and *in vivo* patient-derived xenograft models (Jia et al., 2021).

Moreover, flavonoids exert cross-organ synergistic protective effects *via* the gut-brain axis, with the digestive system serving as the pivotal hub (Xiong et al., 2023). In gut-liver axis-mediated liver diseases, flavonoids exert effects through a synergistic “microbiota-anti-inflammatory-antioxidant” pathway. For instance, anthocyanin-3-O-glucoside reduces inflammation triggered by LPS entering the liver by decreasing the abundance of hydrogen sulphide-producing *Desulfovibrio* bacteria and increasing *Akkermansia* abundance in the gut. Concurrently, flavonoids exert antioxidant effects by activating the hepatic Nrf2 pathway, synergistically mitigating hepatic steatosis with microbiota modulation (Churm et al., 2023; Curtis et al., 2022). In pancreas disorders mediated by the gut-pancreatic axis, taking acute pancreatitis as an example, flavonoids can mitigate secondary pancreatic injury by modulating the gut microbiota. For instance, naringin increases the abundance of *Lactobacillus* species while reducing *Escherichia coli* in the gut, thereby reducing LPS translocation into the bloodstream and preventing inflammation triggered by pancreatic NF-*κ*B activation. Concurrently, they activate the pancreatic Nrf2/HO-1 pathway to exert antioxidant effects, mitigating ROS damage caused by pancreatic enzyme autodigestion. Furthermore, they stimulate intestinal L cells to secrete glucagon-like peptide-1, which inhibits excessive pancreatic enzyme activation by regulating the gut-pancreatic signalling pathway (Churm et al., 2023; Kawakami et al., 2021). This establishes a chain reaction of “microbiota regulation→anti-inflammation→antioxidant effects→pancreatic enzyme regulation”, thereby exerting therapeutic effects on acute pancreatitis. Moreover, flavonoids abundant in the green Mediterranean diet—such as those found in green tea and Mankai plants—can reduce intestinal inflammation and ROS levels by increasing *Prevotella* and decreasing *Bifidobacterium* abundance. Their metabolites exert effects through the gut-liver axis and the gut-pancreatic axis to exert effects on the liver and pancreas, synergistically improving hepatic lipid deposition and pancreatic inflammation, demonstrating a multi-mechanistic, cross-organ synergistic effect (Rinott et al., 2022). In gut-lung axis-mediated pulmonary diseases, baicalein can alleviate dysbiosis by targeting gut microbiota regulation. It significantly enriches beneficial bacteria such as Prevotellaceae and *Bacteroidetes*, while reducing pro-inflammatory bacteria like *Desulfovibrio*, thereby promoting short-chain fatty acid (SCFA) production. Concurrently, the generated SCFAs exert effects on the lungs *via* the gut-lung axis, upregulating FFAR2/FFAR3 receptor expression in pulmonary tissue. This restores the expression of tight junction proteins ZO-1 and claudin, and mucins MUC5b/MUC2 to enhance pulmonary barrier function. Concurrently, it inhibits the TLR4/MyD88/NF-*κ*B signalling pathway and suppresses the release of inflammatory mediators such as IFN-*γ* and TNF-*α*, thereby reducing pulmonary inflammation and oedema. Moreover, this protective effect is entirely lost following antibiotic-induced depletion of gut microbiota, while faecal microbiota transplantation reproduces its therapeutic efficacy. Ultimately, this mechanism improves Staphylococcal Enterotoxin B (SEB)-induced acute respiratory distress syndrome (ARDS) in a microbiota-dependent manner (Hu et al., 2024). In diseases such as age-related cognitive decline mediated by the gut-brain axis, dietary flavonoids exert neuroprotective effects directly by crossing the blood-brain barrier into brain tissue after intestinal absorption. Concurrently, they indirectly contribute to cognitive function improvement *via* the gut-brain axis pathway by regulating gut microbiota and their metabolites (Chu et al., 2024).

The pharmacological mechanisms of flavonoids in digestive system disorders exhibit a systemic characteristic of “multi-target synergism–cross-mechanism chain linkage–physiological axis cross-organ protection”. The multi-tiered synergistic network formed by flavonoids—encompassing anti-inflammatory, antioxidant, and gut microbiota regulation, not only demonstrates their inherent advantages as naturally sourced compounds with multi-target and multi-pathway effects but also provides a direction for drug development targeting digestive system disorders. Future research may focus on multi-target regulatory pathways such as Nrf2/ARE and NF-*κ*B, delving into the specific binding patterns, differential regulatory intensities, and upstream-downstream signal interactions of diverse flavonoids within these pathways. This will clarify key action sites to design more precise structural modification strategies. Formulation development may explore enteric-targeted preparations to enhance localised drug concentrations in the gut, enabling precise release at pathological sites such as the colon while reducing upper gastrointestinal depletion and systemic absorption-related toxicity. Furthermore, in the preventive domain, maintaining intestinal homeostasis could delay disease progression in conditions like MAFLD. This synergistic mode of action aligns with traditional Chinese medicine’s holistic philosophy while establishing a novel paradigm for comprehensive treatment of digestive disorders.

## 3 Conclusion and perspectives

Flavonoids are natural polyphenols widely distributed in fruits, vegetables, tea, and medicinal plants. They exist as glycosides and free forms, serving as key components of plant secondary metabolism and holding significant importance in the prevention and treatment of digestive system diseases (Rakha et al., 2022). Relying on diverse biological activities such as antioxidation, anti-inflammation, immunomodulation, antibacterial, and anticancer effects, they provide multiple protections for gastrointestinal health (Lan et al., 2024). Despite their extensive pharmacological activities, the practical application of flavonoids still faces multiple limitations. Firstly, the extraction of flavonoids from natural plants suffers from low yield (Dong et al., 2022), mainly due to their generally low content in natural plants (Miean and Mohamed, 2001) and significant fluctuations influenced by environmental factors (Wang Y. et al., 2025). To address this issue, stable quality can be achieved by establishing Good Agricultural Practice (GAP) planting bases, optimizing Standard Operating Procedures (SOPs), and regulating with special fertilizers (Yu et al., 2024). Meanwhile, chemical synthesis technologies (total synthesis/semisynthesis) can be used to construct flavonoid parent nuclei and derivatives from basic chemical raw materials, eliminating reliance on plant resources, simplifying processes, shortening cycles, and reducing costs (Bollikolla et al., 2022). In addition, efficient production of flavonoids can be realized by designing and optimizing biosynthetic pathways in microbial and plant chassis. Rational design and modification of microbial chassis can significantly improve product yield (Wang J. et al., 2024), and systematic metabolic engineering technologies represented by Yeast Cell Factories (YCFs) have achieved breakthroughs in this field (Wang J. et al., 2024). For example, Park, N. et al. designed and modified Yarrowia lipolytica to construct a microbial cell factory for the *de novo* biosynthesis of apigenin and acacetin from naringenin. Under fed-batch fermentation conditions with optimized carbon-nitrogen ratio, the final yield of acacetin reached 1.10 g/L, the highest titer reported in microbial synthesis systems to date. This study fully confirms that *Yarrowia lipolytica* is an excellent chassis strain for scalable biosynthesis of flavonoids, providing an efficient solution for the large-scale production of apigenin and acacetin (Park et al., 2025). When plant chassis are used for large-scale production of specific flavonoids, the process flow is more stable. Compared with microbial chassis, their comprehensive cost can be reduced by 5–10 times (Lin J. et al., 2023), and they do not require complex fermentation equipment, are suitable for large-scale planting, and can effectively solve the bottleneck of low flavonoid content in natural plants (Lin J. et al., 2023). For example, Liao, J. et al. developed In-Fusion and 2A peptide linkers, assembling SbCLL-7, SbCHI, SbCHS-2, SbFNSII-2, and SbCYP82D1.1 genes driven by AtPD7, CaMV 35S, and AtUBQ10 promoters with HSP, E9, and NOS terminators, which were used to modify tomato plants for baicalein biosynthesis. Transgenic tomato plants synthesized with this construct produced baicalein ranging from 150 ng/g to 558 ng/g fresh weight (FW). Baicalein-enriched tomatoes have the potential as health-promoting fresh vegetables, providing an alternative source for baicalein production with broad market application prospects (Liao et al., 2022). The extraction of flavonoids from natural plants also faces problems such as low extraction efficiency, high energy consumption, and destruction of thermosensitive components in traditional solvent extraction methods (Carbone and Macchioni, 2025). To solve this, deep eutectic solvents and supercritical fluids can replace traditional solvents (He et al., 2016), combined with ultrasound (Yusoff et al., 2022) and enzyme-assisted (Li S. et al., 2024) extraction of flavonoids. For example, Liu et al. developed ultrasound-assisted supercritical CO_2_ extraction technology to extract lutein from Tropaeolum majus flowers. Compared with traditional supercritical CO_2_ extraction, this technology increased lutein yield by 14.9% and shortened extraction time by 16.7% under mild conditions (L et al., 2025). In addition, Li Y et al. adopted ultrasound-assisted enzyme extraction to extract total flavonoids from Viticis Fructus (VF), and found that the yield was higher than that of traditional ultrasound extraction and single enzyme extraction (Li Y. et al., 2024).

Meanwhile, flavonoids are inherently unstable due to their chemical structural characteristics. Their basic skeleton is a C6-C3-C6 structure, and on the C-ring (pyran ring), the carbonyl group (C=O) and double bond form a conjugated system, which makes the C2-C3 double bond of the C-ring highly reactive (Ichimatsu et al., 2007). Under alkaline conditions, the pyran ring of the C-ring is prone to opening, generating a chalcone structure (Zhang et al., 2023). Chalcones have different chemical properties from flavonoids, and this reaction is reversible under certain conditions. This is the main reason for the darkening of flavonoids in alkaline solutions (Zhang et al., 2023). In addition to ring opening under alkaline conditions, strong acidic conditions may also cause polymerization or structural rearrangement of certain flavonoids (such as flavanols) (Braghiroli et al., 2019). Furthermore, the A, B, and C rings of flavonoid molecules form a large conjugated *π*-bond system. This system enables them to absorb light of specific wavelengths but also makes them susceptible to excitation by light energy, leading to photochemical reactions (such as photooxidative degradation) and structural damage (Glykofridi et al., 2023). Moreover, flavonoids usually contain multiple phenolic hydroxyl groups that are easily oxidized. Particularly, the presence of ortho-phenolic hydroxyl groups (known as catechol or pyrocatechol structure) at the 3′ and 4′ positions of the B-ring drastically increases their oxidative activity, rendering them extremely unstable (Glykofridi et al., 2023). Such flavonoids (such as rutin and quercetin) exhibit strong antioxidant capacity but are also the most vulnerable to degradation (Glykofridi et al., 2023). Therefore, strict control of reaction conditions is required during the extraction, concentration, and drying of flavonoids (Guo et al., 2011). For storage, measures such as brown glass bottles, vacuum packaging, and nitrogen-filled protection should be adopted to improve stability (Ragno et al., 2025). *In vivo*, the stability of flavonoids is affected by the acidic environment of the gastrointestinal tract, metabolic enzymes (such as CYP450 and UGT), and gut microbiota (Oteiza et al., 2018). For example, the stability of anthocyanins is influenced by multiple factors: glycosylation can improve their water solubility, but the hydroxylation pattern of the B-ring enhances antioxidant activity while exacerbating pH-dependent degradation (Kuntz et al., 2015). Additionally, drastic fluctuations in gastric pH (1.8–6.0) can cause irreversible conversion of anthocyanin structures between different forms, further affecting their stability. Furthermore, the enzymatic activity of gut microbiota can lead to nearly complete degradation of anthocyanins within 24 h (Zhang and Zhu, 2023). However, the stability of anthocyanins can be improved through several strategies. For instance, hyaluronic acid-BSA nanoparticles can reduce the coefficient of variation (CV) of anthocyanin release rate in the gastrointestinal tract from 45% (in free form) to 12%. Co-delivery systems with fructooligosaccharides can selectively enrich Bifidobacterium and redirect anthocyanins mainly towards the protocatechuic acid metabolic pathway (Jaouhari et al., 2025), thereby enhancing their stability. Myricetin also faces stability issues, such as gastrointestinal instability (rapid degradation at pH above 6.8) (Devi et al., 2025). Nevertheless, its stability can be optimized through structural modification and formulation innovation. For example, alkylation of the hydroxyl group at the C1 position can enhance gastrointestinal absorption; the design of enteric-coated or gastroretentive formulations can avoid degradation at neutral pH; metal-flavonoid coordination nanoparticles (such as myricetin-AgNPs and myricetin-AuNPs) possess both antibacterial and anticancer activities (Dang et al., 2014) and can also improve stability to a certain extent.

The clinical application of flavonoids is also limited by their low solubility, poor membrane permeability, and extensive metabolism. For example, studies indicate that the oral bioavailability of anthocyanins is extremely low, with less than 0.1%–1% entering systemic circulation in their original form. Furthermore, individual variations significantly influence the metabolic processes of anthocyanins. Specifically, differences in gut microbiota composition—such as variations in the Firmicutes/Bacteroidetes ratio spanning up to fourfold—can lead to markedly distinct metabolite profiles among different individuals (Gonçalves et al., 2021; Oancea, 2021). The oral bioavailability of myricetin is only 9.62%–9.74% (Dang et al., 2014), with limiting factors including extremely low solubility (16.60 μg/mL in water) according to solubility standards from the Chinese Pharmacopoeia (2025), United States Pharmacopoeia (USP35), and European Pharmacopoeia (EP7.8), poor membrane permeability (molecular weight 318.25 g/mol, TPSA as high as 151.59 A^2^), extensive metabolism (at least 38 phase II metabolites produced in rats), and limited distribution (low blood-brain barrier permeability) (Devi et al., 2025). These factors lead to significant fluctuations in blood drug concentrations and insufficient exposure at the target site. Addressing the aforementioned shortcomings, novel delivery systems demonstrate considerable potential for optimization. For anthocyanins, studies have demonstrated that strategies such as spatiotemporal release control (e.g., core-shell structures prepared *via* electrospray ionization achieving gastric release rates <10% and intestinal release rates >70%), optimized transmembrane transport (e.g., liposomal encapsulation increasing peonidin-3,5-diglucoside transport efficiency in Caco-2 cells from 11.7% to 17.3%), and metabolic intervention (e.g., cyclodextrin inclusion complexes reducing microbial degradation of anthocyanins by 40% through inhibiting *Clostridium* histolyticum proliferation in the intestine) have effectively enhanced their bioavailability (Shehata et al., 2023; Chan et al., 2023). For Myricetin, microemulsions increased oral bioavailability in rats by 16.05-fold, TPGS-modified pre-liposomes enhanced bioavailability by 7.2-fold (Thant et al., 2021), and bone-targeting micelles boosted oral exposure by 3.54-fold (Xi et al., 2022). Self-nanoemulsifying drug delivery systems increased drug solubility by 2.5–6.3-fold through 36–127 nm nanoemulsion droplets (Qian et al., 2017), enabling rapid absorption. Solid lipid nanoparticles co-encapsulate antioxidants to extend half-life by 4500-fold, enabling sustained release over 8 h. Nanostructured lipid carriers remain stable in acidic environments, protecting drugs from intestinal degradation (Dang et al., 2014). Mesoporous silica nanoparticles co-loaded with myricetin and siRNA or chemotherapeutic drugs enhance drug loading capacity through their high specific surface area (314 m^2^/g) and achieve synergistic effects by utilizing pH-responsive release in the tumor microenvironment. Additionally, microemulsion-hydrogel systems can improve solubility and tissue targeting while regulating enterohepatic circulation efficiency to enhance drug exposure in target organs. Mechanism-based precision delivery strategies further optimize their clinical application (Dang et al., 2014).

In terms of safety, some flavonoids exhibit toxicity due to their structural characteristics, dose-related toxic side effects, or sensitivity in specific populations. For instance, soy isoflavones and genistein, which possess phytoestrogenic activity, may interfere with fetal growth and development if pregnant women take more than 150 mg/d for a long time (Choi et al., 2008). Dose-related toxic side effects of certain flavonoid components are shown in Supplementary Table S2. Relevant issues can be addressed by exploring detoxification mechanisms, metabolic regulation, formulation approaches, and constructing PK/PD or PBPK models. On the basis of clarifying the specific toxicity mechanisms of flavonoids, active exploration of detoxification strategies is necessary. For example, studies have found that ginsenoside Rb1 can effectively antagonize psoralen dihydroflavone-induced renal fibrosis by scavenging reactive oxygen species and alleviating endoplasmic reticulum stress (Ni, 2022). In addition, metabolic regulation helps reduce toxicity risks. Inhibiting the SULT1A1 enzyme can improve the absorption rate and *in vivo* retention of parent drugs, enabling therapeutic effects in clinical practice without the need for high doses, thereby avoiding toxicity risks associated with high doses. For example, using para-aminobenzoic acid to inhibit the SULT1A1 enzyme (Rasool et al., 2019) can reduce the sulfation metabolism of flavonoids, thereby increasing the absorption rate of parent drugs. Furthermore, drug delivery systems can not only reduce the side effects of active components in traditional Chinese medicine but also achieve precise drug therapy. For instance, a study formulated genistein into colon-targeted nanoparticles to enable its precise release in the colon for regulating gut microbiota health, thus avoiding its metabolic inactivation in other organs or its role as an endocrine disruptor (Wang S. et al., 2025; Yu et al., 2021). To address the nephrotoxicity of anthraquinone components, our team previously prepared rhubarb total free anthraquinones oral colon-specific drug delivery granules, allowing direct drug release in the colon, avoiding absorption in the upper gastrointestinal tract, enhancing laxative effects while preventing nephrotoxicity (Zhang et al., 2015; Liu et al., 2017). Earlier, we jointly developed Sanhuang Dispersible Tablets with enterprises, reducing the single dose from 4 tablets to 1 tablet, and completing the new drug application. Finally, in the dose conversion from animals to humans, the traditional body surface area method ignores interspecies differences in the activity of drug transporters (such as P-gp and BCRP) and metabolic enzymes, which may lead to insufficient doses or toxicity caused by excessive doses in clinical applications (Löscher and Trenité, 2025). Moreover, there are deficiencies in the safety evaluation of high-dose long-term toxicity of some flavonoids (such as quercetin and isoquercetin) and in specific populations (elderly people and patients with hepatic or renal insufficiency) (Manjunath and Thimmulappa, 2022; Yao et al., 2021). To address dose-related limitations, pharmacokinetic-pharmacodynamic (PK/PD) models or physiologically based pharmacokinetic (PBPK) models can be constructed to achieve cross-species dose conversion from rats to humans, and extend adult models to special subgroups, providing a basis for drug doses in different populations and facilitating the development of precision medicine. For example, a study measured the concentration of related substances and prolactin levels in rats, constructed a PK-PD model using NONMEM software, and verified it through visual predictive checks. After correcting for interspecies differences with *in vitro* data and predicting human parameters, human data were used to simulate pharmacodynamic effects. Final clinical verification confirmed that the parameters were consistent with the predicted values, demonstrating the translational value of the PK-PD model (Chang et al., 2011) For example, Li X et al. constructed a physiologically based pharmacokinetic (PBPK) model of Schaftoside, validated the model using pharmacokinetic data of total styrene flavonoids after intravenous injection and oral administration in rats, and then extrapolated the PBPK model to humans using PK-Sim® software, verifying its consistency with clinical data from healthy volunteers. Finally, core parameters were identified through sensitivity analysis to support extrapolation to special populations. This indirectly verified the stability of the “preclinical→human” correlation and demonstrated the translational potential of the PBPK model (Li X. et al., 2022).

Currently, several flavonoid compounds or flavonoid-rich medicinal products approved for treating digestive system disorders include kaempferol (Liu Q. et al., 2024), citrus aurantium total flavonoid tablets (Deng et al., 2024), baicalin tablets (Xiong et al., 2018), and Xianglian Pills (Guan et al., 2025). Additionally, flavonoid compounds such as soy isoflavones [Identifier: NCT02026518 (Jalili et al., 2016)], silymarin [Identifier: NCT00680407 (Navarro et al., 2019)], and proanthocyanidins [Identifier: IRCT20190731044392N2 (Ghanbari et al., 2024)] are also in clinical research phases (Supplementary Table S3). However, flavonoids continue to face challenges in clinical translation. On the one hand, clinical trials suffer from issues such as limited sample sizes, non-standardised designs, inconsistent dosing and administration regimens, and a lack of studies in special populations. These shortcomings result in insufficient clinical evidence, thereby restricting the translational application of flavonoid compounds. Specifically, the limited sample size manifests as trial cohorts often comprising fewer than 100 subjects, rendering it difficult to account for individual variations and medication patterns across different populations (Savino et al., 2023). Substandard design is characterised by the absence of randomised controlled trials (RCTs) and placebo controls, with efficacy assessments often relying on subjective symptoms such as pain relief, resulting in low study credibility (Akhmetzianov and Bredikhin, 2021). Future efforts should focus on conducting multicentre, large-scale RCTs, standardising dosage and administration protocols, incorporating special populations such as the elderly and children, and employing objective biochemical indicators alongside symptom assessments to evaluate efficacy, thereby strengthening clinical translation (Liu Y. et al., 2025). On the other hand, flavonoid compounds face regulatory and industrialisation barriers including ambiguous drug registration classifications and approval standards, high industrialisation costs coupled with uncertain market returns, and significant challenges in intellectual property protection. These obstacles hinder their transition from research outcomes to market-ready pharmaceuticals. Regulatory confusion over the classification of natural extract flavonoids has previously complicated application pathways. For instance, the absence of uniform content standards for glycyrrhizin increased export compliance costs (Authord). Policies may be leveraged to establish clear standards. The National Medical Products Administration’s 2020 Classification and application document requirements for traditional Chinese medicine registration categorises TCM into innovative drugs, modified new drugs, and other types (National Pharmacopoeia Committee, 2020). HQY total flavonoids capsules, jointly developed by Nanjing University of Chinese Medicine and industry partners, secured clinical trial approval as a category 1.2 innovative TCM. The 2025 edition of the Pharmacopoeia of the People’s Republic of China proposes adding licorice flavonoid content standards. Enterprises may also align with USP/EP standards to optimise impurity control and testing, thereby mitigating export risks (National Pharmacopoeia Committee, 2025). Regarding industrialisation, traditional processes suffer from low yields, high energy consumption, and lengthy R&D cycles that make returns difficult to predict. These challenges can be addressed through technological upgrades and policy support. The 14th 5-year plan for the development of traditional Chinese medicine encourages integrated cultivation-extraction-formulation systems. The Nanjing University-hospital-enterprise collaborative CPU-216 project completed preclinical research within 2 years, accelerating translation to secure returns (Author). Regarding intellectual property protection, natural flavonoids face patent rejection or infringement risks due to structural commonalities. Early extraction techniques often prove difficult to protect, necessitating strategic patent positioning. China Pharmaceutical University’s CPU-216 project secured four patents centred on flavonoid active components and preparation processes, achieving value conversion through a ¥100 million transfer. The Hushenyuan team secured an invention patent for Huoshan Dendrobium methyl transferase and its application in flavonoid synthesis, establishing a patent barrier by pinpointing core innovations (Clinical Trials). In summary, the future of flavonoid-based treatments for digestive disorders will centre on AI-empowered mechanisms, formulations, and clinical applications. Artificial intelligence (AI) will facilitate target prediction, multi-omics analysis of interactions, optimisation of co-crystals and processes, development of personalised regimens, trial refinement, and regulatory alignment to drive full-chain transformation (Drug Discovery Faster, 2025).

## Footnotes

1
https://www.nanjing.gov.cn/zzb/ywdt/njxx/202404/t20240423_4214395.html
