# Supplementary material for: The pharmacological effects and therapeutic potential of flavonoids in digestive diseases
Source: Front Pharmacol. 2026 Mar 26;16:1684377. doi: 10.3389/fphar.2025.1684377 (PMC13063306; doi:10.3389/fphar.2025.1684377)
Supplement: Supplementary file 1 [file Supplementaryfile1.docx]

Supplementary Material

# Supplementary Table

**Table S1.** Interactions of flavonoid signaling pathways in digestive system diseases

| **Disease** | **Compound** | **Model/Species** | **Target molecule** | **Interaction mode/pattern** | **Reference** |
| --- | --- | --- | --- | --- | --- |
| Reflux esophagitis | Quercetin | HET-1A cells | c-jun N-terminal kinase, p38 MAPK, NF-*κ*B | Phosphorylation inhibition | [14] |
| Eosinophilic esophagitis | 7,4'-dihydroxyflavone | RAW 264.7 cell line | TNF-*α*, IL-6, IL-1*β*, MAPK1, AKT, CCND1 | Direct binding, phosphorylation inhibition | [16] |
|  | Isoliquiritigenin | Murine model | TNF-*α*, IL-4, IL-5, TGF-*β*1, PPE-s-IgE | Direct binding, inhibition of gene expression | [17] |
| Esophageal squamous cell carcinoma | Luteolin | Nude mice | FAK, Src, AKT, ErbB2 | Direct binding, inhibition of gene expression | [19] |
|  | Tangeretin | Nude mice | GLI2, GPNMB | Direct binding, expression inhibition | [20] |
|  | 5,7,4'-trimethoxyflavone | Patient-derived xenograft mouse models | LRPPRC, JAK2, STAT3, CDK1 | Direct binding, functional inhibition | [21] |
|  | Cirsiliol | ESCC cells | TYK2 | Direct binding | [22] |
| Gastritis | Eupatilin | AGS human gastric cancer cells | CagA, NF-*κ*B | Expression inhibition | [23] |
|  | Vitexin | Sprague-Dawley rats | NLRP3 | Direct binding | [24] |
| Gastric ulcer | Apigenin | Wistar rats | TGF-*β*1, COX-2, TNF-*α*, NF-*κ*B, Bax, Bcl-2, MDA, SOD, CAT | Phosphorylation inhibition, | [25] |
|  | Genistein | Sprague-Dawley rats | Wnt, *β*-catenin, TGF-*β*, Sma and Mad-related protein, 4 PKB | Phosphorylation inhibition | [26] |
| Gastric carcinoma | Zapotin, Pectolinarigenin | SNU-1 gastric cancer cell line | m-TOR, PI3K, AKT, BAX, BCL-2, Caspase-3, Caspase-8, Caspase-9 | Phosphorylation inhibition | [27] |
|  | Chrysin | Gastric cancer cells | miR-18, miR-21, miR-221, let-7a, miR-9, miR-22, miR-34a, miR-126 | Indirectly affect the expression of their target genes through the regulatory mechanisms of miRNAs | [29] |
|  | Naringin | AGS cells | PI3K, AKT, mTOR signaling pathway, Beclin 1, LC3B | Phosphorylation inhibition | [30] |
| Viral hepatitis A | Icariin(ICA), pICA | DHAV-1-induced model,  duck embryo hepatocytes | Nrf2, TLR, NF-*κ*B, MMP, MitoROS, ATP | Direct binding or indirect activation, phosphorylation inhibition | [32] |
|  | Baicalin | DHAV-1-induced model, duck | Nrf2/ARE signaling pathway | Direct binding | [33] |
| Viral hepatitis B | 3,5,6,7,3',4'-  hexamethoxyflavone | HepG2.2.15 and HepG2-NTCP cells, HBV transgenic C57BL/6 mice | HBsAg, HNF3*α*, HBV RNA, HBV DNA, cccDNA | Inhibition expression | [34] |
|  | Baicalin | BALB/c mice | ER*α*-AMPK*α*-HNF pathway | Cascade regulation | [36] |
|  | Dihydromyricetin | HepG2.2.15 cells | NF-*κ*B, MAPKs, HNF4*α*, TLR4, autophagy-related proteins | Multi-target regulation | [37] |
| Viral hepatitis C | Silymarin | HCV(Hepatitis C Virus)-infected model, HCV Patients | Oxidative stress, hepatic function, hematological, hormonal pathways | Synergistic enhancement | [38] |
| Alcoholic liver disease | Quercetin | Alcoholic liver disease(ALD) model, C57BL/6J mice | Rab7-V1G1 axis | Regulatory axis | [39] |
|  | Kaempferol, Nicotiflorin |  | Nrf2 and SIRT1 signaling pathways | Multi-target regulation | [40] |
|  | Nobiletin | Bmal1flox/flox mice | BMAL1-AKT axis | Phosphorylation activation | [41] |
|  | Hydroxysafflor yellow A | ALD model, C57BL/6 mice | PI3K/AKT and STAT3/NF-*κ*B pathways | Phosphorylation activation | [42] |
|  | Puerarin |  | EGFR, RAF1, ERK1/2 | Phosphorylation inhibition | [43] |
|  | Oligomeric proanthocyanidins |  | ROS, MLKL, CTSB, NLRP3 | Oxidative、phosphorylation、leakage、inflammasome activation | [46] |
| Autoimmune hepatitis | Vitexin | Autoimmune hepatitis model, C57BL/6 mice | AMPK, AKT, GSK-3*β*, Nrf2 | Phosphorylation activation | [48] |
| Metabolic dysfunction-associated fatty liver disease | Rutin | MAFLD model, db/db male mice | AMPK, Nrf2, ACC, | Phosphorylation activation, inhibition | [50] |
|  | Isoquercitrin | MAFLD model, C57BL/6 mice | NLRP3 | Inhibition | [51] |
|  | 7-Hydroxyflavone |  | STK24 | Direct binding | [52] |
|  | Kaempferol | NASH model, C57BL/6 mice | NLRP3, ASC, TMS1, caspase-3 | Direct binding | [57] |
| Liver fibrosis | Breviscapine |  | TAK1, JNK, p38, IKK*β*, p65 | Phosphorylation inhibition | [58] |
|  | Procyanidin B2 | CCl4-induced mouse liver fibrosis model | VEGFA, HIF-1*α*, collagen I, TGF-*β* | Inhibition | [59] |
|  | Flavokawain A | TGF-*β*1-stimulated vascular smooth muscle cells | Nrf2, ARE, MMP-9/2 | Activation, downregulation | [60] |
|  | Hesperidin | Thioacetamide-induced liver fibrosis model, male albino rat | TGF-*β*/*α*-smooth muscle actin | Inhibition | [61] |
|  | Eupatilin | CCl_4_-induced liver fibrosis model, C57BL/6J mice | *β*-catenin, plasminogen activator inhibitor-1, *α*-SMA | Protein expression inhibition | [62] |
| Hepatocellular carcinoma | Morusin | Hep3B mouse xenograft model | ATP-citrate lyase | Direct binding | [66] |
|  | Oroxylin A | HCC cells | ROCK1, NLRP3, ASC | Activation | [67] |
|  | Glabridin | H22 cell-established tumor-bearing mouse model | DUSP5, ZFP36, KLF10, NR4A1 | Upward adjustment | [68] |
|  | Calycosin-7-glucoside | HepG2 xenograft mice | Thioredoxin-1 | Targeted regulation | [69] |
|  | Quercetin | Huh7 and Hep3B cells | SIRT1, PTEN-induced kinase 1 | Upward adjustment | [70] |
|  | (R)-7,3'-dihydroxy-4'-methoxy-8-methylflavane (DHMMF) | Orthotopic hepatic carcinoma mouse models | p21, PLK1 | Upward adjustment | [71] |
|  | Baicalin, Scutellarin | HepG2 and MHCC97-H cells | JAK2, STAT3 | Phosphorylation inhibition | [73] |
|  | Icaritin | HCC cells | PINK1, Parkin | Upward adjustment | [74] |
|  | Flavokawain C | Tumor-bearing mice | FAK, PI3K | Phosphorylation inhibition | [75] |
| Acute liver failure | Luteolin-7-O-rutinoside | LPS/D-galactosamine-triggered acute liver failure in mice | PI3K, AKT, AMPK, NF-*κ*B | Phosphorylation inhibition | [76] |
|  | Naringin | Acetaminophen-induced liver damage model, C57BL/6 mice | CHAC2, Nrf2 | Upgrade activation | [77] |
|  | Hesperetin | Acute liver injury model, BALB/c mice | SIRT1, NF-*κ*B p65 | Upgrade activation, deacetylation inhibition | [78] |
| Schistosomiasis | Licochalcone B | Schistosoma mansoni | SmATPDase 1 | Inhibition | [85] |
| Acute pancreatitis | Tricetin | Cerulein-induced acute pancreatitis mice | PARP1, NF-*κ*B | Inhibition | [86] |
|  | Baicalein | LPS-induced AR42J PACs | miR-224-5p, PARP1, NF-*κ*B65, NLRP3, caspase-1 | Direct binding, expression suppression, indirect inhibition | [87] |
|  | Pinocembrin | Rat model | TLR4, NLRP3, NF-*κ*B, miR-34a-5p, HO-1 | Combination, inhibition, upregulation | [88] |
|  | Isorhamnetin | Sodium taurocholate-induced severe acute pancreatitis mice | KDM5B, HtrA2 | Inhibition, upregulation | [89] |
|  | Galangin | L-arginine-induced severe acute pancreatitis mice | Nrf2, HO-1 | Upregulation | [90] |
|  | Tectoridin | Caerulein-induced severe acute pancreatitis mice | ERK2 | Phosphorylation inhibition | [91] |
|  | Dihydrokaempferol | Severe acute pancreatitis mice | Keap1 | Inhibition | [92] |
| Chronic pancreatitis | Eruberin A | LTC-14 cells | PI3K, AKT, NF-*κ*B | Phosphorylation inhibition | [93] |
| Pancreatic cancer | Fisetin | Pancreatic cancer cells | MISP, *β*-catenin | Promote nuclear accumulation | [95] |
|  | Tiliroside | PANC-1 cell xenograft mice | Calpain-2 | Direct binding | [96] |
|  | Xanthohumol, Plumbagin | KPC transgenic mice | BCL2, pSTAT3 | Combination inhibition | [97] |
|  | Taxifolin | Pancreatic cancer cells | IGF1R, INS, INSR, VEGFA | Binding | [98] |
| Jejunal mucosal injury | Dihydromyricetin | Weaned piglets | Nrf2, TLR4, p-NF-*κ*B, HIF-1*α*, NLRP3 | Activation, downregulation, phosphorylation inhibition, inhibition, activation inhibition | [100] |

### Table S2. Experimental toxicity-adverse reaction dosage table of flavonoids

| Compound | Experimental subject | administration route | dose | toxic | Adverse reaction | species difference | Reference |
| --- | --- | --- | --- | --- | --- | --- | --- |
| β-naphthoflavone | Male rats | Gavage | 60mg/kg | Exists digestive system toxicity and hepatotoxicity | Can induce the expression of Cytochrome P450 1A1(CYP1A1) in rat colon/liver microsomes | Not mentioned | [S1] |
| chrysin |  |  |  |  |  |  |  |
| quercetin |  |  |  |  |  |  |  |
| flavone |  |  |  |  |  |  |  |
| naringenin |  |  |  |  |  |  |  |
| diosmin |  |  |  |  |  |  |  |
| rutin |  |  |  |  |  |  |  |
| Kaempferol | Isolated enterocytes from male Hartley guinea pigs | In vitro culture | 450 μM | Causes death of intestinal epithelial cells, exhibiting digestive system toxicity | Cell viability decreased by 12%–60%, with intestinal epithelial cells leaking lactate dehydrogenase (LDH). Treatment with quercetin and myricetin induced superoxide production, exhibiting greater toxicity than kaempferol | Not mentioned | [S2] |
| Quercetin |  |  |  |  |  |  |  |
| Myricetin |  |  |  |  |  |  |  |
| Rutin | Healthy male C57BL/6 mice | Gavage | 70mg/kg/day | Non-toxic | None | Rutin must be metabolised by gut microbiota into quercetin before synergistically inducing toxicity with IQ (via the gut-liver axis). Animal studies demonstrate toxicity only upon combined exposure to rutin and IQ, with no toxicity observed from either compound alone. However, rutin and IQ (derived from high-temperature processed meats) may coexist in the human diet. Given differences in gut microbiota composition between humans and mice, the metabolic efficiency and toxicity risk require further validation | [S3] |
| Heterocyclic amine (2-amino-3-methylimidazo[4,5-f]quinoline, IQ) |  |  | 7mg/kg/day | Non-toxic | None |  |  |
| Rutin＋IQ |  |  | Rutin: 70 mg/kg bw/day  IQ: 7 mg/kg bw/day | Hepatotoxicity, gastrointestinal toxicity and metabolic toxicity are present. | 1. Liver damage: Alanine Aminotransferase(ALT) and Aspartate Aminotransferase(AST) levels significantly elevated; GSH, CAT, and SOD activity decreased; MDA content increased; disorganised hepatocyte arrangement and inflammatory cell infiltration observed; liver index reduced; significant elevation in liver TNF-α, IL-6, and IL-1β levels.  2. Intestinal damage: Shortening of the colon, villous shedding, disruption of crypt architecture, and elevated inflammatory cytokines (TNF-α, IL-6, IL-1β) in colonic tissue. Gut microbiota dysbiosis: Decreased Firmicutes/Bacteroidetes ratio, reduced abundance of beneficial bacteria (Lachnospiraceae, Roseburia), and increased pathogenic bacteria (Eggerthellaceae).  3. Metabolic disorders: Significant abnormalities in hepatic lipid metabolism, tryptophan metabolism, and bile acid metabolic pathways; reduced short-chain fatty acid (acetate, propionate, total SCFAs) content |  |  |
| Luteolin | Swiss mice (free from underlying diseases, healthy strain) | Intraperitoneal injection | ＞100 mg/kg | Acute hepatotoxicity | Serum ALT and AST levels increased, with heightened hepatic oxidative stress; Hepatocyte damage was observed in the 200 mg/kg group | Differences in metabolic enzyme activity: Human CYP1A1/CYP1A2 exhibits lower activation efficiency for luteolin compared to mice; rodent livers metabolise flavonoids at a faster rate, leading to greater accumulation | [S4] |
|  | Human lymphoblastoid TK6 cells (normal cell line) | In vitro processing | Direct treatment following activation by CYP1A1/CYP1A2 | Genotoxicity exists | Enhance genotoxicity, inducing DNA damage |  |  |
| Quercetin | L5178Y/Tk⁺/⁻-3.7.2C mouse lymphoma cell line | In vitro processing | 30-100 μM | Genotoxicity exists | Significantly elevated Tk gene mutation frequency, with a global evaluation factor exceeding 126×10⁻⁶, inducing double-strand DNA breaks and activating DNA damage pathways (elevated expression of γ-H2AX, p-Chk1 and p-Chk2). LOH analysis revealed >90% of mutants exhibited loss of heterozygosity on chromosome 11, involving deletions of distinct segments | The same cell line exhibits varying sensitivities to different flavonoids, specifically: Quercetin (30 μM positive) > Kaempferol (50 μM positive) > Quercetin-3-β-D-glucoside (400 μM positive) | [S5] |
| Kaempferol |  |  | 50 μM-200 μM |  |  |  |  |
| Quercetin-3-β-D-glucoside |  |  | 400-600 μM |  |  |  |  |
| Coumoestrol | Healthy volunteers' peripheral blood lymphocytes | In vitro experiments | 50 μM、75 μM、100 μM | Genotoxicity exists | At 50 μM, the rate of distorted cells increased three to fourfold; at 75 μM, the distorted cell rate reached 40% (primarily exhibiting staining of monomeric clefts, breaks, and exchanges), accompanied by a slight decrease in the mitotic index | Hamster V79 cells constitute an immortalised cell line with weaker DNA repair capacity than human primary lymphocytes; human lymphocytes require phytohaemagglutinin (PHA) stimulation for proliferation and exhibit slightly higher tolerance to toxicity | [S6] |
|  | Healthy China Hamster Lung Cells (V79 Cells) |  |  |  | Induction of DNA strand breaks and micronucleus formation |  |  |
| Genistein | Healthy volunteers' peripheral blood lymphocytes |  | 25 μM |  | At 25 μM, the rate of distorted cells reached 35% (excluding cleft cells), with a 56% reduction in the mitotic index |  |  |
|  | Healthy China Hamster Lung Cells (V79 Cells) |  |  |  | Induction of DNA strand breaks and micronucleus formation |  |  |
| Quercetin | Swiss–Webster mice | Intraperitoneal injection | 2×625 mg/kg  2×1250mg/kg  2×2500mg/kg | Genotoxicity exists | All three doses significantly increased the DNA damage index and damage frequency in bone marrow cells | In the same mouse model, quercetin exhibited significantly higher genotoxicity (DNA damage, chromosomal damage) than rutin, which only caused mild DNA damage at moderate doses. This discrepancy may arise because rutin requires hydrolysis by intestinal microbiota glycosidases to convert into quercetin before exerting its toxic effects, and the efficiency of this hydrolysis in vivo is limited. resulting in lower free quercetin concentrations compared to direct administration | [S7] |
|  |  |  |  |  | Only the 2×1250 mg/kg dose significantly increased the micronucleus rate in polychromatic erythrocytes (PCE) in bone marrow |  |  |
| Rutin |  |  |  |  | Only the 2×1250 mg/kg dose significantly increased DNA damage. |  |  |
|  |  |  |  |  | None of the doses significantly increased the micronucleus rate, and no chromosomal damage was observed |  |  |
| Nitrosated quercetin | Salmonella Typhimurium strain TA100 | In vitro experiments | 0.167 mM | bacterial toxicity exists | Ames assay: Mutagenicity of 6.4 revertants per nanomole, meaning each nanomole of nitrosated quercetin induces 6.4 revertant colonies | Salmonella typhimurium TA100 exhibits greater sensitivity to mutation induction by quercetin nitrosation products than by malvidin-3-glucoside, whilst Escherichia coli PQ37 demonstrates significantly higher SOS pathway induction capacity by quercetin nitrosation products than by malvidin-3-glucoside | [S8] |
|  | Escherichia coli strain PQ37 |  |  |  | SOS-induced potential (SOSIP) was 0.56 nmol, indicating that each nanomole of nitrosated quercetin significantly activated the SOS repair pathway, with elevated β-galactosidase activity |  |  |
| Nitrosated malvidin-3-glucoside | Salmonella Typhimurium strain TA100 |  |  |  | Ames test: Mutagenicity of 2.1 revertants per nanomole |  |  |
|  | Escherichia coli strain PQ37 |  |  |  | The SOSIP is 0.07 nmol, indicating a relatively weak capacity to induce DNA damage |  |  |
| Genistein | Newborn mice | subcutaneous injection | Undefined dosage | Reproductive toxicity exists | Increased number of uterine glands, elevated uterine weight, heightened epithelial cell density, and increased rate of ovarian corpus luteum deficiency in adulthood. | Metabolic differences: Rodent gut microbiota exhibit higher metabolic activation efficiency for flavonoids than humans, with human flavonoids predominantly excreted in conjugated forms; Hormonal pathways: Rodent reproductive systems exhibit heightened sensitivity to phytoestrogens compared to humans, particularly during juvenile stages; 3. Exposure thresholds: Human dietary intakes (from soybeans, vegetables) remain substantially below experimental toxicological doses, whereas rodent experimental dosages frequently exceed actual human exposure levels | [S9] |
|  | Transgenic Prostate Cancer Mice (TRAMP mice) | Food additives | 250 mg/kg |  | Prostate cancer exhibits increased proliferation and metastatic potential |  |  |
|  | Healthy male C57BL/6 mice | Perinatal exposure | 100 μg/g/day | Neurotoxicity is present | Increased defensive behaviour and reduced aggression in adulthood |  |  |
|  | Health CD1 Mice | Food additives | 100 μg/g |  | Changes in anxiety and aggressive behaviour in adulthood |  |  |
| EGCG | Male CF-1 mice (healthy adult animals) | Intraperitoneal injection | 750 mg/kg (moderate to severe hepatic necrosis); 1500 mg/kg (85% survival rate) | Hepatotoxicity exists | Induces an inflammatory cascade reaction, elevated plasma ALT, and hepatocyte necrosis. |  |  |
|  | Human (Health) | Oral | ＞1200 mg / day |  | Some subjects developed liver damage and had to withdraw from the trial; individual variations are associated with genetic factors or lifestyle. |  |  |
|  | Chain urea zoenocin-induced diabetic mice | Intraperitoneal injection | 100 mg/kg | Nephrotoxicity exists | Increased oxidative stress in renal tissue, elevated serum creatinine levels, and pathological renal tissue damage (glomerular atrophy, tubular necrosis). | Mice exhibit extreme sensitivity to EGCG hepatotoxicity (necrolysis occurs at 750 mg/kg), whereas humans require daily doses exceeding 1200 mg before risks become apparent, reflecting differences in metabolic capacity. Diabetic mice demonstrate heightened susceptibility to nephrotoxicity, whilst normal mice show no significant renal damage. |  |
| Quercetin | Human liver cytoplasm (derived from healthy liver tissue) | In vitro experiments | IC₅₀<1 mM | Metabolic toxicity exists | Inhibition of P-type phenylsulphate transferase (PST)-mediated drug sulphonation reactions may affect drug metabolism. | 1. Metabolic differences: Rodent hepatic CYP enzymes exhibit higher metabolic activation efficiency for flavonoids than humans, with human flavonoids predominantly excreted in conjugated forms;  2. Developmental sensitivity: Amphibian embryos are highly susceptible to teratogenic effects of flavonoids during embryonic development, while the reproductive systems of young rodents are sensitive to phytoestrogens;  3. Exposure threshold: Human dietary intake (approximately 1 g/day) is substantially lower than experimental toxic doses, whereas rodent experimental doses often reach pharmacologic/toxicologic levels. | [S10] |
|  | Male F344 rats (healthy adult animals) | Intraperitoneal injection | 10 mg/kg | Hepatotoxicity exists | Oxidative damage to hepatocytes |  |  |
| Epigallocatechin gallate | CD-1 mice (healthy adult animals) | Intraperitoneal injection | 120 mg/kg | Hepatotoxicity | Elevated plasma ALT levels by fourfold indicate hepatocyte injury. |  |  |
|  | Male F344 rats (healthy adult animals) | In vivo experiments | Undefined dosage | Digestive system toxicity exists | When used alone, it enhances the development of colon cancer induced by dimethylhydrazine. |  |  |
| Naringenin | Human intestinal CYP3A4 enzyme (derived from healthy intestines) | In vitro culture | Undetermined concentration (in grapefruit juice) | Digestive system toxicity exists | Inhibits intestinal CYP3A4 activity, interfering with the metabolism of drugs such as calcium channel blockers and triggering drug interactions. |  |  |
|  | Amphibian embryo (Natterjack toad, developmental model) | In vitro culture | 10 mg/L | Embryotoxicity exists | 100% embryonic malformations (reduced body size, axial curvature, renal hypoplasia, etc.), 30% malformed embryos die |  |  |
| Silymarin | Male F344 rats (healthy adult animals) | Feed additives | 12,500–50,000 parts per million (approximately 1,050–4,500 milligrams per kilogram per day) | Reproductive toxicity exists | Sperm motility decreased by 5%, 11% and 9% respectively | 1. Metabolic differences: Humans exhibit higher metabolic efficiency in glucuronidation and methylation of silymarin compared to rodents, resulting in lower accumulation of toxic metabolites;  2. Dose threshold: Common clinical doses in humans (420–2100 mg/day) are substantially lower than the toxic dose observed in rodents (>1000 mg/kg/day);  3. Physiological differences: Rodent reproductive and immune systems exhibit greater sensitivity to high-dose flavonoids, whereas human sperm quality remains minimally affected | [S11] |
|  | Female B6C3F1 mice (healthy adult animals) | Feed additive/day | 25,000–50,000 parts per million (approximately 4,800–9,680 milligrams per kilogram) | Immune system toxicity exists | Thymus weight was significantly reduced; over the two-year feeding period, body weight in this dose group decreased compared to the control group |  |  |
|  | Pregnant ICR mice (healthy pregnant mice) | Oral administration, continued during pregnancy | 50–200 milligrams per kilogram per day | Genotoxicity exists | Reduced foetal weight, facial, spinal and cranial deformities, with a dose-dependent potential teratogenic risk |  |  |
|  | Healthy volunteers (without underlying medical conditions) | Oral | 140 mg three times daily (420 mg per day) | Mild gastrointestinal toxicity is present. | No significant toxicity; a small number of individuals experienced nausea and abdominal distension; well tolerated |  |  |
|  | Chronic hepatitis C patients (pathological condition) |  | 280–700 mg three times daily (840–2100 mg per day) | Gastrointestinal/neurological toxicity exists | The 280 mg dose group experienced nausea and headache; the 560 mg dose group experienced mild abdominal pain and upper respiratory tract infections, all of which were mild to moderate in severity and transient in nature |  |  |
|  | Prostate cancer patients (pathological status) |  | 2.5–20 mg silibin-phospholipid complex (Siliphos) three times daily | Mild gastrointestinal toxicity is present | Gastrointestinal discomfort and hyperbilirubinaemia may occur; in a minority of cases, slight elevations in creatinine and serum calcium levels, along with halitosis, have been observed. No severe toxicity has been reported |  |  |
|  | Diabetic patients (pathological condition) |  | 140 mg three times daily (420 mg per day) | Minor gastrointestinal/neurological toxicity is present | Headache, nausea, and vomiting occurred. One patient experienced myocardial infarction due to a pre-existing cardiac condition (not directly related to silymarin) |  |  |
| Quercetin | Healthy golden hamsters (6 weeks old, equal numbers of males and females, free from underlying conditions) | Rearing | 96-960 mg  dietary supplement | Non-toxic | None | Norwegian rats exhibit higher bioavailability or target affinity for quercetin in intestinal and bladder tissues. Quercetin may exert potential carcinogenic effects in Norway rats by inducing intestinal/bladder epithelial cell proliferation and DNA damage. This pathway is not activated in other species. Differences in metabolic efficiency and intestinal absorption characteristics between species result in safety thresholds varying by a factor of 100 (the safe dose for other species is over 100 times that for Norway rats) | [S12] |
|  | Norwegian rats (healthy, specific strain) |  | 20-2000mg dietary supplement | Organ toxicity exists | The incidence of tumours in the ileum and bladder increased significantly, representing a strain-specific toxicological response |  |  |
|  | Healthy ACI rats (without underlying conditions) |  | 20-2000mg dietary supplement | Non-toxic | None |  |  |
|  | Albino rats (without underlying diseases) |  | 20-2000mg dietary supplement |  |  |  |  |
| Quercetin | Hepatocyte nuclei derived from healthy male Sprague-Dawley rats | In vitro processing | 50–200 μM | Antioxidant system toxicity exists | Glutathione (GSH) depletion: exhibited concentration-dependent reduction, with the myricetin group decreasing to 0.212 μmol/mg protein at 200 μM; - Glutathione S-transferase (GST) activity inhibition: the myricetin group decreased to 0.317 μmol/min·mg protein at 200 μM. Subsequently, this induces oxidative stress, leading to nuclear membrane lipid peroxidation and DNA strand breaks, which may in turn induce mutagenicity and carcinogenicity | Within the same rat hepatocyte nuclear model, myricetin exhibited the strongest toxicity (highest number of hydroxyl groups), kaempferol the weakest (lowest number of hydroxyl groups), and quercetin intermediate toxicity. This aligns with the structure-activity relationship that ‘the greater the number of hydroxyl groups, the stronger the pro-oxidative activity’. - activity relationship. Furthermore, as hepatocyte nuclei are directly exposed to flavonoids without the metabolic buffering of the liver as a whole, toxicity manifests immediately. In tissue, flavonoids must undergo intestinal absorption and hepatic metabolic conversion; the effective concentration actually reaching hepatocyte nuclei may be reduced, potentially leading to toxicity profiles differing from those observed in in vitro experiments | [S13] |
| Myricetin |  |  |  |  |  |  |  |
| Kaempferol |  |  |  |  |  |  |  |
| Chrysin (5,7-dihydroxyflavone, 97% purity) | Sprague-Dawley rats, equal numbers of males and females | Gavage | Acute toxicity dose：5000 mg/kg | Hepatotoxicity, nephrotoxicity, haematopoietic and reproductive toxicity | 1. Liver damage: Significant elevation of ALT, AST, Gamma-Glutamyl Transferase(GGT), bilirubin, and albumin; decreased SOD and GSH activity in liver tissue; increased MDA and NO content; central lobular necrosis, chromatin condensation, vascular congestion, and large-vacuolar fatty degeneration in hepatocytes; inflammatory cell infiltration.  2. Renal injury: Biochemical indicators: marked elevation of creatinine;  Glomerular structural disruption, thickening of the basement membrane, accumulation of cellular debris within the tubular lumen, nuclear fragmentation and condensation, with inflammatory cell infiltration in the interstitium | In males: reduced MCH and MCHC, decreased TLC and neutrophil counts, elevated lymphocyte counts; mild interstitial oedema in testes, with significant weight loss in the 1000 mg/kg group.  In females: decreased RBC count during the recovery period, elevated TLC during the treatment period. Males exhibited greater sensitivity to weight loss and increased liver weight, while certain haematological abnormalities in females were observed solely during the recovery period | [S14] |
|  |  |  | Subchronic toxicity dose：1000 mg/kg/day |  |  |  |  |
| Catechin | Gram-positive bacteria: Bacillus subtilis  （Bacillus subtilis 168） | In vitro culture | Cultured at concentrations of 5, 10, and 15 parts per million | Cytotoxicity is present | At a concentration of 9 ppm, catechins exert a significant inhibitory effect on bacterial growth | 1. Differences in cell wall structure: The cell wall of Bacillus subtilis contains multiple layers of peptidoglycan, exhibiting strong affinity for catechols, which facilitates catechol binding and disruption of the cell membrane. In contrast, the outer layer of Escherichia coli's cell wall consists of negatively charged lipopolysaccharides, which repel the hydroxyl groups of catechols, resulting in low binding efficiency and poor permeability.  2. Differences in damage severity: Following catechin treatment, Bacillus subtilis exhibits pronounced cell membrane damage, DNA degradation (supercoiled DNA converting to open-ended DNA), and filamentous cell morphology; Escherichia coli shows only minor cell membrane damage and cell thinning, with no significant DNA damage.  3. Differences in ROS effects: Bacillus subtilis' ROS fluorescence signal quenched due to peptidoglycan dehydration/concentration, whereas Escherichia coli's ROS fluorescence signal increased over time, though its overall oxidative damage level remained lower than that of Bacillus subtilis | [S15] |
|  | Gram-negative bacteria: Escherichia coli  （Escherichia coli MTCC 40） |  |  |  |  |  |  |
| luteolin | Artemia salina | Contact administration | BST assay: Ethyl acetate (EA) extract of cumin exhibited an LC₅₀ of 52.40 μg/ml, whilst the hexane (HE) extract demonstrated an LC₅₀ of 60.77 μg/ml. | fatal | The extract and its active subcomponents caused brine shrimp mortality, exhibiting a direct lethal effect. | The extract exhibits low cytotoxicity towards normal mouse cells. Its toxicity to brine shrimp, as determined by whole-organism assays, differs from the mammalian cell toxicity dosage and mechanism. | [S16] |
| apigenin | Healthy male Swiss-Webster mice | Intraperitoneal injection | 2 mg/kg strychnine, 5 mg/kg apigenin-7-O-glucoside | Neurotoxicity, enhanced toxicity | Exacerbates strychnine toxicity, manifested by an earlier onset of tonic-extensional convulsions (TEC) and a shorter time to death; When 5 mg/kg flavonoids were combined with 2 mg/kg strychnine, the onset of TEC occurred within 1.9–2.3 minutes, with death occurring within 2.2–3.0 minutes. This represents a 2–3-fold reduction compared to strychnine alone (TEC onset at 5.6 minutes, death at 6.3 minutes) | The sensitivity of apigenin (IC₅₀ ranging from 26.9 to 44.7 μmol/L) differs from that of endogenous glycine receptors *in vivo*. The effective dose in mice (1–5 mg/kg intraperitoneal injection) is significantly lower than the concentration used in in vitro cell experiments when converted. This discrepancy may be related to receptor distribution, drug metabolism, and synergistic effects within the organism | [S17] |
| luteolin-7-O-glucoside |  |  |  |  |  |  |  |
| apigenin-7-O-glucoside |  |  |  |  |  |  |  |
| quercetin |  |  |  |  |  |  |  |
| Genistein |  |  |  |  |  |  |  |

### Table S3. Clinical application progress of flavonoids in digestive system diseases

| **Clinical Stage/**  **Marketed** | **Ingredients** | **Indications** | **Sample size** | **Trial quality** | **Endpoints** | **Risk of bias** | | **Clinical value** | **References** | **Identifier** |
| --- | --- | --- | --- | --- | --- | --- | --- | --- | --- | --- |
| Marketed | XiangShaLiuJunZi decoction (with ingredients including *Citrus reticulata* and *Glycyrrhiza*)  flavanones, flavonols | Gastric cancer or colorectal cancer | 128 cases | Single-center, open-label, randomized controlled trial | The primary endpoint is the change in superoxide dismutase levels. Secondary endpoints include oxidative stress markers, inflammatory cytokines, and quality of life assessed by the SF-36 questionnaire. Safety endpoints involve monitoring adverse events. | The overall risk of bias is low. Potential limitations include single-center recruitment , open-label design (potential for implementation bias), and a long follow-up period . | | Combining the tumor-suppressing effects of S-1 capsules with the benefits of Xiang Sha Liu Jun Zi Tang in reducing chemotherapy-related adverse reactions and improving quality of life, this approach offers a low-cost, convenient means to enhance survival outcomes for patients with advanced gastrointestinal cancer, aligning with China's healthcare needs. It also fills the gap in maintenance therapy combining chemotherapy drugs with traditional Chinese medicine, providing evidence for developing relevant integrated Chinese and Western medicine guidelines and promoting treatment protocols, thereby advancing clinical application. | [S18] | ChiCTR: ChiCTRINR16008575 |
|  |  | Functional dyspepsia (FD) | 216 cases | Multicenter, randomized, double-blind, placebo-controlled clinical trial | The primary endpoint was the change in postprandial discomfort severity scores after 4 weeks of treatment. Safety endpoints included adverse events and laboratory parameters such as blood and urine routine tests and biochemical indicators. Results demonstrated that the treatment group outperformed the placebo group across all efficacy endpoints without any adverse events. | The overall risk of bias is low. Potential limitations include a follow-up period of only 8 weeks and no explicit mention of controlling for multiple comparisons bias. | | It alleviates symptoms such as postprandial bloating in patients with functional dyspepsia, enhances gastric emptying rates, improves quality of life and emotional well-being without adverse reactions. This provides a safe new Traditional Chinese Medicine (TCM) option for those unresponsive to chemical drugs, offers high-quality evidence for TCM treatment of this condition, and supports its clinical adoption. | [S19] | ChiCTR: ChiCTRTRC13003200  ClinicalTrials.gov: NCT02762136 |
|  |  | Irritable bowel syndrome | 82 cases | Single-center, randomized, double-blind, placebo-controlled clinical trial | The primary endpoint was the change in relevant scores. For safety endpoints, no significant differences in adverse events were observed between groups, and indicators such as liver and kidney function remained stable. | Overall, the results are generally low. Potential limitations include single-center design, small sample size, short treatment duration, lack of literature references for sample size estimation, limited effectiveness of certain indicators, and insufficient data on generalizability and long-term efficacy. | | Significantly improves diarrhea in patients with irritable bowel syndrome, demonstrates good safety without affecting liver or kidney function, provides an effective traditional Chinese medicine treatment option for this condition, and offers high-quality evidence supporting the efficacy and safety of Chinese herbal medicine for treating this disorder, thereby facilitating its clinical adoption. | [S20] | ClinicalTrials.gov: NCT02179580 |
|  | Chaihu Shugan san (with ingredients including *Citrus reticulata* and *Aurantii Fructusand Glycyrrhiza*)  flavanones, isoflavones | Functional dyspepsia (FD) | 94 cases | Single-center, randomized, double-blind, placebo-controlled clinical trial | The primary endpoint was SDS scores at 4 weeks. Safety endpoints showed few and mild adverse events. | Overall results were low, with potential limitations including single-center design, only 4 weeks of follow-up, and a small sample size for gut microbiota analysis, limiting generalizability. | | Significantly improves symptoms in patients with functional dyspepsia, enhances gastric emptying rate, alleviates anxiety and depression, improves quality of life, and regulates gut microbiota. This provides a safe and effective traditional Chinese medicine treatment option for this condition, while also offering high-quality evidence for the efficacy of Chinese herbal medicine in treating functional dyspepsia through gut microbiota regulation, thereby facilitating its clinical adoption. | [S21] | ChiCTR: ChiCTR2100045793 |
|  | Moluodan (with ingredients including *Artemisiae Scopariae Herba* and *Radix Sanguisorbae*)  flavonoids, flavonols | Chronic atrophic gastritis | 502 cases | Multicenter, randomized, double-blind, folic acid parallel-controlled clinical trial | The primary endpoint is the 1-year rate of staged improvement and resolution of dysplasia. The safety endpoint is the absence of drug-related serious adverse events. | Overall low quality, with potential limitations including the absence of a placebo control, uneven group allocation (2:2:1:1), inclusion of only Chinese populations, and lack of integration with endoscopic scoring. This restricts the study's generalizability and the assessment of effect magnitude. | | It can safely improve the histological staging of gastric mucosa in patients with precancerous gastric lesions, demonstrating superior efficacy to folic acid in reversing low-grade dysplasia, with higher doses showing a trend toward greater therapeutic benefit. This provides a new traditional Chinese medicine option for the chemoprevention of this disease and offers high-quality evidence for its clinical implementation. | [S22] | ChiCTR: ChiCTR1800016087 |
|  |  | Chronic atrophic gastritis with mild to moderate dysplasia | 196 cases | Multicenter, randomized, double-blind, double-dummy, folic acid parallel-controlled clinical trial | The primary endpoint is the 1-year rate of staged improvement and resolution of dysplasia. The safety endpoint is the absence of drug-related serious adverse events. | Overall results were modest, with potential limitations including the absence of a placebo control, uneven group allocation, inclusion of only Chinese populations, and lack of integration with endoscopic scoring. This restricts the generalizability and assessment of the magnitude of effect. | | This approach can safely and effectively reverse gastric precancerous lesions and improve histological staging of gastric mucosa. The high-dose regimen shows a trend toward greater therapeutic efficacy, offering a safe and effective new TCM option for the chemoprevention of gastric precancerous lesions while providing high-quality evidence-based support for its clinical implementation. | [S23] | ChiCTR: ChiCTRTRC00000169 |
|  | Xianglian Pills (with ingredients including *Coptis chinensis Franch*)  flavonols | Diarrhea (1 week after cholecystectomy) | 90 cases | Single-center, open-label, randomized controlled clinical trial | The primary endpoints were histological scores, pathological response rates (with higher rates of atrophy or intestinal metaplasia response in the Morodan group), and the rate of resolution of dysplasia. The safety endpoint was the absence of drug-related serious adverse events. | Overall results were modest, with potential limitations including single-region enrollment, short follow-up duration (6 months), high dropout rate, absence of placebo control, and insufficient data on generalizability and long-term efficacy. | | It significantly reduces the dysplasia score in patients with chronic atrophic gastritis complicated by dysplasia, alleviates gastric mucosal symptoms and discomforts such as epigastric pain and belching, and demonstrates good safety. This provides an effective traditional Chinese medicine treatment option for the condition and offers high-quality evidence-based support for its clinical application. | 199 | ChiCTR: ChiCTR2200061854 |
|  | Yixinshu capsule (with ingredients including *Radix Paeoniae Alba*, *Glycyrrhizae Radix*)  flavonols, flavanones, isoflavones | Functional dyspepsia （PDS） | 197 cases | Multicenter, randomized, double-blind, placebo-controlled clinical trial | The core focus is on linking chemical quality assessment with biological mechanisms to ensure drug quality consistency and uncover the relationship between chemical composition and efficacy. | Overall results are relatively low, with potential limitations including limited extrapolation from single-manufacturer samples and a lack of external independent sample validation. | | Accurately evaluating the quality consistency of Yixinshu capsules and identifying its core components, targets, and pathways for treating heart failure through network pharmacology provides scientific evidence for standardizing the quality and ensuring the clinical safety of Yixinshu Capsules. It also offers a replicable innovative technical framework for quality control and mechanism research of other traditional Chinese medicine formulations. | [S24] | ClinicalTrials.gov: NCT02460601 |
|  | Gegen Qinlian formula (with ingredients including *Scutellaria baicalensis*,*Pueraria lobata*)  flavonoids, isoflavones | Advanced colorectal cancer | 120 cases | Multicenter, randomized, parallel-controlled interventional study | Primary endpoints: SF-36 quality of life score, TCM symptom score, progression-free survival (PFS), and overall survival (OS). Safety endpoints: Monitoring of adverse events such as nausea. | The overall risk of bias is relatively low. Potential limitations include the non-double-blind design (with implementation bias), inclusion of only patients with damp-heat syndrome, and possible loss to follow-up during the 2-year follow-up period, which restrict the extrapolation of results. | | It is expected to improve patients' quality of life, alleviate TCM symptoms, prolong survival, and regulate immunity and the tumor microenvironment. This provides a new integrated traditional Chinese and Western medicine option that enhances efficacy and reduces toxicity for the disease, as well as high-quality clinical evidence for the treatment of this type of tumor with traditional Chinese medicine. | [S25] | ChiCTR: ChiCTR2100050754 |
|  | Gastrosis No.1 compound (with ingredients including *Glycyrrhiza uralensis* and *Citrus reticulata*)  isoflavonoids, flavonoids | Functional dyspepsia (FD) | 162 cases | Multicenter, randomized, double-blind, placebo-controlled clinical verification study | Primary endpoints: total dyspepsia symptom (TDS) score and single-item SDS score at 4 weeks and 8 weeks. Safety endpoints: no serious adverse events and no abnormalities in laboratory indicators. | The overall risk of bias is relatively low. Potential limitations include a follow-up duration of only 8 weeks (without long-term data) and failure to clearly control for multiple comparison bias, which somewhat restrict the extrapolation of results. | | It is safe and effective in treating functional dyspepsia of spleen-stomach deficiency-cold type, as it can significantly improve core symptoms such as postprandial fullness, early satiety, and epigastric pain, as well as TCM syndrome scores. The therapeutic effect persists for 4 weeks after drug withdrawal, providing a reliable TCM treatment option for this syndrome type of the disease and offering high-quality evidence-based support for its clinical promotion. | [S26] | ChiCTR: ChiCTRTRC10001074 |
|  | Modified LiuJunZi decoction(with ingredients including(*Glycyrrhizae Radix*)  flavonoids, isoflavones | Functional dyspepsia (FD) | 160 cases | Multicenter, randomized, double-blind, placebo-controlled clinical verification study | Primary endpoints: TDS score, single-item dyspepsia symptom (SDS) score (double assessment by researchers and patients). | Random allocation concealment is well-implemented, and the double-blind design reduces subjective bias. The outcome indicators include both symptom scores and objective gastric emptying tests, resulting in a relatively low risk of bias. However, there are limitations such as a short follow-up period and unclear reporting of details regarding the maintenance of blinding. | | As a Phase II clinical study (based on its multi-center design, sample size scale, and the purpose of verifying efficacy and safety), it confirms the effectiveness and safety of Jiawei Liujunzi decoction in treating functional dyspepsia of spleen deficiency and qi stagnation type, providing evidence-based support for its clinical promotion. | [S27] | ChiCTR: ChiCTRTRC10001074 |
|  | Qushi Huayu (with ingredients including *kaempferol* and *resveratrol*）  flavonols | MAFLD associated with abnormal liver function | 246 cases | Multicenter, randomized, double-blind, double-dummy, parallel-controlled clinical trial | Primary endpoints: VCTE-CAP score (hepatic fat) and serum alanine aminotransferase (ALT) at 24 weeks. Safety endpoints: no serious adverse events (AEs), the incidence of AEs in the two groups was similar. | The overall risk of bias is relatively low. Potential limitations include the absence of a placebo group, MRI-PDFF being conducted only in a single center, and incomplete control of confounding factors related to diet and lifestyle, which restrict the extrapolation of results. | | It is safe and effective in the treatment of non-alcoholic fatty liver disease. Compared with salvianolic acid capsules, it more significantly reduces serum ALT, aspartate aminotransferase (AST), and liver fibrosis FIB-4 score. It can also regulate intestinal microbiota imbalance and aromatic amino acid metabolism, providing a new and effective traditional Chinese medicine treatment option for this disease and offering high-quality evidence-based support for its clinical promotion. | 193 | ChiCTR: ChiCTRIOR17013491 |
|  | Lingguizhugan decoction (with ingredients including (*Glycyrrhizae Radix*))  flavonols | MAFLD | 243 cases | Protocol for a multicenter, randomized, double-blind, three-arm dose-optimized, placebo-controlled clinical | Primary endpoint: The proportion of patients with a reduction of ≥ 1 unit in HOMA-IR at 12 weeks. Safety endpoints: monitoring of vital signs, blood tests, electrocardiograms, and adverse events. | The overall risk of bias is relatively low. Potential limitations include recruitment from a single region, a follow-up duration of only 16 weeks (without long-term data), and reliance on ultrasound for hepatic fat assessment (with lower accuracy than MRI), which restrict the extrapolation of results. | | It focuses on the efficacy and optimal dosage for treating non-alcoholic fatty liver disease of the spleen-yang deficiency type, with the core goal of improving insulin resistance. It evaluates the effects on liver and kidney functions, metabolic indicators, as well as intestinal and oral microbiota. It is expected to provide a safe and effective traditional Chinese medicine regimen for this syndrome type of the disease, and also lay a foundation for community promotion and mechanism research. | [S28] | ChiCTR: ChiCTR1800014364 |
|  | AnluoHuaxian pills(with ingredients including *Rehmannia glutinosa* and *Panax notoginseng*)  flavonoids, flavonols | chronic hepatitis B | 270 cases | Multicenter, randomized, double-blind, placebo-controlled clinical trial | Primary endpoints: Improvements in NCT-A, MMSE, and P300 waves after 15 days of treatment; the Jiedu Huayu Granules group showed better effects than the lactulose group, and the combined group exhibited a synergistic effect. Safety endpoint: No drug-related adverse events. | | The overall risk of bias is moderate. Potential limitations include the non-double-blind design (with implementation/measurement bias), small sample size, short follow-up duration of only 15 days (without long-term data), and single-center setting, which restrict the extrapolation of results. | It can safely improve cognitive and neurophysiological functions in patients with cirrhosis complicated by minimal hepatic encephalopathy, with better efficacy than lactulose and a synergistic effect when used in combination. This provides a new and effective traditional Chinese medicine option for the disease and offers clinical evidence for integrated traditional Chinese and Western medicine treatment. | [S29] | ChiCTR: ChiCTRIOR14005474 |
| Phase Ⅰ | Jieduhuayu granules | Liver cirrhosis with minimal hepatic encephalopathy (MHE) | 80 cases | Single-center, randomized, open-label, controlled clinical trial | Primary endpoints: improvements in NCT-A, MMSE, and P300 waves after 15 days of treatment; the Jiedu Huayu Granules group showed better effects than the lactulose group, and the combined group exhibited a synergistic effect. Safety endpoint: No drug-related adverse events. | The overall risk of bias is moderate. Potential limitations include the non-double-blind design (with implementation/measurement bias), small sample size, short follow-up duration of only 15 days (without long-term data), and single-center setting, which restrict the extrapolation of results. | | It can safely improve cognitive and neurophysiological functions in patients with cirrhosis complicated by minimal hepatic encephalopathy, with better efficacy than lactulose and a synergistic effect when used in combination. This provides a new and effective traditional Chinese medicine option for the disease and offers clinical evidence for integrated traditional Chinese and Western medicine treatment. | [S30] | ANZCTR:  ACTRN12614000193673 |
|  | Ma Zi Ren Wan | Functional constipation | 291 cases | Prospective, double-blind, double-dummy, randomized, controlled clinical trial | Primary endpoint: response rate of complete spontaneous bowel movements during the 8-week treatment period. Safety endpoints: monitoring of liver and kidney functions and adverse events. | The overall risk of bias is relatively low. Potential limitations include recruitment from a single region, short follow-up duration, and reliance on experts' experience for TCM syndrome differentiation, which restrict the extrapolation of results. | | By comparing Maziren pills with senna and placebo, this study evaluates the efficacy and safety of Maziren pills in treating functional constipation of TCM excess syndrome type, clarifies its improving effects on the rate of complete spontaneous bowel movements, colonic transit function, and constipation symptoms. It provides an effective TCM treatment option for this disease and offers high-quality research evidence for comparing the efficacy of Chinese and Western laxatives. | [S31] | ClinicalTrials.gov: NCT01695850 |
|  | Hezhong Granules (with ingredients including Citrus reticulata, Crataegus pinnatifida and Glycyrrhiza uralensis)  flavanones, flavonols, isoflavones | Nausea and vomiting induced by chemotherapy in advanced colorectal cancer | 120 cases | Multicenter, randomized, double-blind, placebo-controlled clinical trial | Primary endpoints: complete response rate and objective response rate for acute and chronic chemotherapy-induced nausea and vomiting. Safety endpoint: absence of serious adverse events. | The overall risk of bias is relatively low. Potential limitations include being conducted in a single region, a low proportion of female participants, and reliance on patient diaries for data recording, which restrict the extrapolation of results. | | When Hezhong Granules are combined with 5-hydroxytryptamine 3 receptor antagonists and dexamethasone for patients with advanced colorectal cancer, they can safely prevent chemotherapy-induced nausea and vomiting (CINV). In particular, they significantly improve the complete response rate and objective response rate for delayed nausea and vomiting, and also enhance quality of life. This provides a new integrated traditional Chinese and Western medicine option for the prevention and treatment of CINV, as well as high-quality evidence-based support for traditional Chinese medicine as an adjuvant to cancer chemotherapy. | [S32] | ChiCTR: ChiCTR2100041643 |
|  | Xiaoji Daozhi decoction (with ingredients including *Paeonia lactiflora Pall.*)  flavonols | Childhood constipation | 200 cases | Single-center, double-blind, randomized, placebo-controlled trial | Primary endpoints: remission rate at 8 weeks of treatment and 12 weeks of follow-up, and the number of complete spontaneous bowel movements (≥ 3 times/week). Secondary endpoints: satisfaction with defecation function and adverse reactions. | The risk of bias is low, blinding and randomization are well-implemented. However, the single-center design may limit the extrapolation of results. | | It provides a safe and effective TCM regimen for childhood constipation. It is confirmed that Xiaoji Daozhi decoction has a remission rate of 62%, a low recurrence rate, and no serious adverse reactions. This fills the gap in TCM clinical research on childhood constipation, provides an alternative to western medicine laxatives in pediatrics, and improves the long-term constipation management effect in children. | [S33] | ClinicalTrials.gov: NCT03186079 |
|  | Compound kushen injection(with ingredients including *Sophora flavescens*)  flavones | advanced colorectal cancer | 320 cases | Multicenter, open-label, randomized controlled clinical trial | Primary endpoint: PFS; Secondary endpoints: Overall Survival (OS), 1-year OS/PFS rates, Objective Response Rate, Disease Control Rate, and quality of life (Functional Assessment of Cancer Therapy-Colorectal. | The open-label design may lead to subjective bias in the assessment of efficacy and safety, which requires verification by subsequent real-world data. | | It provides high-level evidence support for the integrated traditional Chinese and Western medicine treatment of advanced colorectal cancer. A multi-center randomized controlled study evaluates the efficacy of compound kushen injection combined with first-line regimens, while exploring metabolomics and intestinal microbiota mechanisms. If proven effective, it can be included in clinical guidelines, promoting the standardized application of traditional Chinese medicine in the treatment of advanced colorectal cancer. | [S34] | ClinicalTrials.gov: NCT05894694 |
|  | S*ilybum marianum*,*pueraria lobata* and *salvia miltiorrhiza*  flavonoids | MAFLD | 121 cases | Multicenter, double-blind, randomized, placebo-controlled trial | Primary endpoint: A reduction of ≥ 2 points in the NAFLD Activity Score (NAS). Secondary endpoints: improvement in liver fibrosis, changes in liver enzymes, and homeostatic model assessment for insulin resistance (HOMA-IR). | At baseline, 63% of patients failed to meet the enrollment criteria based on central pathological review of biopsies, resulting in selection bias. Additionally, the small sample size affects the judgment of efficacy. | | It provides a reference for the research and development of botanical drugs for non-alcoholic steatohepatitis (NASH). Although silymarin was not proven to significantly reduce the NAS score, the high dose showed good safety, and some patients exhibited a trend of improved liver fibrosis. This lays the foundation for subsequent optimization of its dosage form, combination regimens, or screening of populations that may benefit most. | [S35] | ClinicalTrials.gov: NCT05076058 |
|  |  | The potential ameliorative effect on hepatic steatosis and fibrosis in patients with liver cirrhosis | 13 cases | Multicenter, randomized, parallel-controlled, double-blind design (non-triple-blind), with administration using the "3+3" dose escalation method | Primary endpoints: maximum tolerated dose (MTD) and reduction of hepatic *γ*-OHPdG (in most patients). Secondary endpoint: pharmacokinetics (PK) (consistent clearance at 400-1200mg). Exploratory endpoint: no new hepatocellular carcinoma (HCC) cases, and reduction of cirrhosis-associated polypeptides (CAP) in some patients. | The overall risk of bias is relatively low. Potential limitations include early trial termination (failure to reach the 2000mg dose), small sample size, invalid immunohistochemistry (IHC) results, and lack of a placebo control, which restrict the extrapolation of results. | | It confirms the tolerability of polyphenon E at a maximum dose of 1600mg per day in cirrhotic patients, and its ability to reduce hepatic *γ*-OHPdG levels. This provides key safety and efficacy evidence for recommending a starting dose of 1200mg per day in subsequent phase II HCC prevention trials. | [S36] | ClinicalTrials.gov:  NCT03278925 |
|  | *Silybin*  flavonoids | MAFLD and chronic hepatitis C (HCV) | 40 cases | Multicenter, randomized, double-blind, placebo-controlled design | Primary endpoints: single-dose/steady-state pharmacokinetic parameters of silybin A/B and their conjugates. Safety endpoint: absence of serious adverse events. Exploratory endpoint: discovery of silymarin enterohepatic circulation in MAFLD, which is absent in HCV. | The overall risk of bias is relatively low. Potential limitations include a small sample size, inclusion of only non-cirrhotic patients, and failure to verify differences in transporter/enzyme expression, which restrict the extrapolation of results. | | It clarifies the impact of different liver disease types on silymarin disposition, providing key pharmacokinetic basis for dose optimization in subsequent phase II efficacy trials of silymarin in NASH patients. | [S37] | ClinicalTrials.gov  NCT00389376 |
|  |  | Obesity | 12 cases | Double-blind, randomized, placebo-controlled, crossover pilot trial | Primary endpoints: daily fecal fat excretion during the 3-day treatment period. Secondary endpoints: changes in intestinal microbiota composition and diversity, clinical parameters, incidence of adverse reactions, and health-related quality of life scores. | Low-risk factors include randomized grouping, double-blind design, and standardized allocation concealment, which reduce selection bias and subjective assessment bias. Potential risks are small sample size, short intervention period, and the inclusion of participants with normal weight, which limit the extrapolation of results. There is no mention of a multi-center design, and there is a lack of verification of consistency in indicator detection. | | Natural compounds have fewer adverse reactions than synthetic drugs and are safe for intestinal microbiota, so they can be used as a supplementary treatment option for obese patients. They provide preliminary evidence for the pancreatic lipase inhibitory effect and safety of subsequent long-term multi-center studies in obese populations, promoting the application of natural flavonoids in the field of anti-obesity. | [S38] | ClinicalTrials.gov: NCT05069298 |
|  | AXP107-11, genistein sodium salt dihydrate | Chemotherapy-naive unresectable pancreatic cancer | 16 cases | Single-center, open-label, dose-escalation clinical trial | Primary endpoints: safety, maximum tolerated dose, and pharmacokinetics. Secondary endpoints: efficacy . | Limitations include a single-center design, small sample size, and lack of a placebo control, which may lead to selection bias and efficacy assessment bias. | | It provides a new direction for the combined treatment of unresectable pancreatic cancer. It is confirmed that AXP107-11 (a novel crystalline form of genistein) combined with gemcitabine has good safety and potential efficacy, which helps determine the dose and combination regimen for subsequent Phase II/III trials and promotes the application of plant-derived drugs in the treatment of pancreatic cancer. | [S39] | EudraCT: 201001921425 |
|  | Anthocyanins | Inhibitory effect on the migration of pancreatic cancer cells *in vitro* | 35 cases | Randomized, double-blind, placebo-controlled, crossover crossover clinical trial | Primary endpoint: changes in the migration ability of PANC-1 and AsPC-1 pancreatic cancer cells *in vitro.* Secondary endpoints: expression of cell adhesion molecules in cancer cells and endothelial cells, cytokine levels, activation levels of NF-*κ*B p65 and FAK, and ROS production. | Low bias, randomization and blinding are well-implemented. However, the dissolution of the powder group was not monitored, which may lead to intervention implementation bias. | | It confirms the inhibitory effect of anthocyanins and their metabolites on pancreatic cancer cell migration as well as the underlying molecular mechanisms, providing new targets and insights for nutritional intervention and combined therapy of metastatic pancreatic cancer. | [S40] | DRKS: DRKS00014767 |
|  | *Isoflavonoides sojae*  isoflavonoids | Irritable bowel syndrome in women（IBS） | 100 cases | Single-center, randomized, double-blind, 2×2 factorial design controlled clinical trial | Primary endpoint: change in irritable bowel syndrome IBS-SSS score. Secondary endpoints: IBS-QOL score, total IBS score. | Low bias, the factorial design can assess interaction effects, but the small sample size may lead to bias in subjective symptom scoring. | | It provides a direction for adjuvant treatment of IBS in women. It is confirmed that soy isoflavones or vitamin D used alone can improve the IBS-SSS score and quality of life, and their combined use has a synergistic effect on the total IBS score. This provides a basis for hormonal regulation and nutritional intervention in IBS, and is particularly suitable for female patients with estrogen-related IBS or vitamin D deficiency. | 200 | ClinicalTrials.gov: NCT02026518 |
|  | Modified banxia xiexin decoction (with ingredients including *Scutellaria baicalensis*, *Glycyrrhiza uralensis* and *Citrus reticulata*)  flavones, isoflavonoids, flavanones | Stage IV gastric cancer | 146 cases | Randomized, parallel-controlled design, with an open-label setting | Primary endpoint: overall survival from patient enrollment to 18 weeks. Secondary endpoints include progression-free survival within 18 weeks, quality of life score, efficacy of TCM syndromes, changes in tumor marker levels, and changes in T cell subsets. | The open-label design, where both subjects and researchers are aware of group assignments, is prone to bias in subjective indicators such as quality of life scores and TCM syndrome assessment; the single-center design limits the extrapolation of results to other regions/medical centers; the lack of mention of a multi-person blind evaluation consistency calibration process for tumor progression assessment leads to bias in objective indicator evaluation; and the absence of reference to the establishment of a Data Monitoring Committee (DMC) results in unclear information on trial quality monitoring. | | Focusing on the palliative chemotherapy stage of stage IV gastric cancer, this study explores the combined application mode of traditional Chinese medicine and chemotherapy. If proven effective, it can provide a new combined treatment option for patients at this stage, especially suitable for those with "spleen qi deficiency and intermingled cold and heat" syndromes. It can also supplement clinical evidence of traditional Chinese medicine in tumor palliative treatment and promote the standardized application of integrated traditional Chinese and Western medicine in tumor treatment. | [S41] | ClinicalTrials.gov: NCT05908838 |
|  | Jianpi Huoxue Formula (with ingredients including (*Astragalus membranaceus* ang *Astragalus membranaceus*) flavonoids, isoflavonoids | Gastric precancerous lesions | 480 cases | Multicenter, randomized, parallel-controlled, triple-blind design | Primary endpoints: the disappearance rate of gastric intestinal metaplasia and changes in pathological scores at 6 months. Secondary endpoints: changes in endoscopic performance scores, changes in main symptom scores, and changes in PRO scale scores at 6 months. | Although the trial adopted a multi-center design, it did not clarify the actual enrollment status and data consistency of all collaborating centers. There was a lack of a "multi-person blind evaluation consistency calibration" process for pathological histological scoring and endoscopic performance assessment, leading to subjective evaluation bias. Additionally, it did not mention the establishment of a DMC, resulting in incomplete information on quality monitoring during the trial. | | The large sample size of 480 participants combined with the multi-center design enhances the reliability of the results. Standardized operations and triple-blind design reduce bias, which can provide high-quality evidence-based medical evidence for traditional Chinese medicine intervention in precancerous lesions of gastric cancer. If proven effective, it can form a mature and promotable treatment plan, fill the gap in standardized treatment of precancerous lesions of gastric cancer, and promote the clinical application of traditional Chinese medicine in the secondary prevention of gastric cancer. | [S42] | ClinicalTrials.gov: NCT03823248 |
| Phase Ⅱ | EGCG  flavonoids | Radiotherapy-related acute radiation esophagitis（ARIE） | 51 cases | self-before-and-after comparison | Primary endpoint: ARIE. Secondary endpoints: tumor remission rate, 1/2/3-year OS. | There is no control group, and the sample size is small, which may lead to subjective bias in the assessment of efficacy and toxicity. | | It provides an effective remission regimen for radiotherapy-related ARIE in esophageal cancer. It is confirmed that EGCG can reduce the RTOG score of ARIE without affecting tumor efficacy, providing a basis for toxicity management in esophageal cancer patients receiving radiotherapy and helping to improve radiotherapy completion rate and survival benefits. | [S43] | ClinicalTrials.gov: NCT01481818 |
|  |  | Acute radiation esophagitis（ARIE） | 83 cases | Prospective three-arm randomized controlled clinical trial | Primary endpoint: remission rate of ARIE (reduction in RTOG score). Secondary endpoints: pain score, dysphagia score, and radiotherapy completion rate. | There is no control group, and the study uses a single-center design, which may lead to subjective bias in efficacy assessment. | | It addresses the challenge of ARIE in radiotherapy for stage III lung cancer. It is confirmed that EGCG can significantly reduce the RTOG score and pain score of ARIE without affecting the radiotherapy process, providing a safe and convenient new method for clinical prevention and treatment of ARIE, and improving the radiotherapy tolerance and quality of life of lung cancer patients. | [S44] | ClinicalTrials.gov: NCT02577393 |
|  |  | Effect of acute EGCG supplementation on gastric emptying in healthy women | 23 cases | Single-center, randomized, double-blind, placebo-controlled, crossover clinical trial | Primary endpoint: gastric emptying rate (relative gastric volume). Secondary endpoints: appetite score (Visual Analog Scale [VAS]), blood glucose, and hormones (insulin, adiponectin). | Low bias, the crossover design reduces individual differences. However, only healthy women were included, which limits the extrapolation of results, and there may be slight bias in subjective appetite scores. | | It confirms that acute EGCG supplementation can delay gastric emptying, providing clinical evidence for subsequent research on its application in weight management and appetite regulation. | [S45] | ReBEC: RBR-9svwrv |
|  | Silymarin | Metabolic dysfunction-associated steatohepatitis (MASH) | 116 cases | Multicenter, randomized, double-blind, placebo-controlled clinical trial | Primary endpoint: a reduction of ≥ 2 points in the MAFLD Activity Score (NAS). Secondary endpoints: improvement in liver fibrosis, changes in liver enzymes, and insulin resistance. | Moderate bias, there is bias in baseline biopsy screening, and the small sample size affects the judgment of efficacy. | | It provides support for the research and development of plant-derived drugs for MAFLD. It is confirmed that the preparation containing bergamot and wild artichoke extracts can reduce liver fat content, and the remission rate is higher in patients over 50 years old. This provides a direction for age-stratified treatment and drug development of MAFLD. | 201 | ClinicalTrials.gov: NCT00680407 |
|  |  | MASH | 148 cases | Randomized, double-blind, placebo-controlled clinical trial | Primary endpoint: A reduction of ≥30% in the Metabolic Associated Fatty Liver Disease Activity Score (NAS); Secondary endpoints: Improvement in liver fibrosis (assessed by histology/liver stiffness measurement), liver enzymes, and glycolipid metabolism indicators. | Low bias; randomization and blinding reduce bias. However, details of multi-center quality control are not specified, and the sample size of liver biopsies is small (with insufficient biopsy length in some patients), which may lead to sampling bias. | | It provides a new direction for nutritional intervention in MAFLD. It is confirmed that specific components (such as SPS) can reduce inflammatory factors and enhance acetaldehyde dehydrogenase activity. Although it does not significantly improve liver fat content, it provides a basis for the subsequent optimization of nutritional support regimens for MAFLD and is suitable for early intervention in patients with mild MAFLD. | [S46] | ClinicalTrials.gov: NCT02006498 |
|  | *Quercetinum*  flavonols | Gastroesophageal reflux disease（GERD） | 100 cases | Single-center, double-blind, randomized, placebo-controlled clinical trial | Primary endpoint: remission rate of ARIE (reduction in RTOG score). Secondary endpoints: pain score, dysphagia score, and radiotherapy completion rate. | There is no control group, and the study adopts a single-center design. Subjective outcomes (pain/dysphagia) rely on patient reporting, which may lead to assessment bias. | | It provides a reference for non-pharmacological intervention of GERD. Although benesco™ does not significantly improve treatment success rate, it offers a direction for subsequent exploration of local intervention methods for GERD. It is particularly suitable for GERD patients who are intolerant to acid suppressants or need to reduce drug dependence. | [S47] | DNTR: NL9324 |
|  | *Proanthocyanidins* | Gastric cancer, high Helicobacter pylori infection | 522 cases | Double-blind, randomized, placebo-controlled clinical trial | Primary endpoints: *H. pylori* negative rates at 2 weeks and 8 weeks. Secondary endpoint: eradication after 45 days of drug withdrawal. | Low bias, randomization and blinding are well-implemented. However, the dissolution of the powder group was not monitored, which may lead to intervention implementation bias. | | It provides a non-antibiotic adjuvant intervention strategy for Helicobacter pylori (*H. pylori*) infection. It is confirmed that high-altitude anthocyanin cranberry juice can achieve a 20% *H. pylori* negative rate with good safety, offering a new option to reduce antibiotic abuse and drug resistance. It is particularly suitable for the long-term management of populations at high risk of *H. pylori* infection. | [S48] | ChiCTR: ChiCTR1800017522 |
|  | *Anthocyanins* | ulcerative colitis (UC) | 100 cases | Multicenter, randomized, double-blind, placebo-controlled clinical trial | Primary endpoints: Liver enzymes and glucose-lipid metabolism indicators. Secondary endpoints: inflammatory factor (TNF-*α*), insulin resistance , and fibrosis marker. | Low bias, randomization and blinding reduce bias. However, the diagnosis of MAFLD relies on ultrasound (not the biopsy gold standard), which may lead to diagnostic bias. | | It provides a new idea for the adjuvant treatment of UC. It is confirmed that ACRE can reduce fecal calprotectin (an inflammatory marker). Although there is no statistically significant difference in clinical remission rate, it provides a basis for the subsequent optimization of natural component intervention regimens for UC and is suitable for inflammatory control in patients with mild to moderate UC. | [S49] | ClinicalTrials.gov: NCT04000139 |
|  | *Anthocyanin* | MAFLD | 116 cases | CONSORT-compliant, single-center, randomized, double-blind, placebo-controlled pilot pilot clinical trial | Primary endpoint: change in plasma alanine transaminase (ALT) concentration. Secondary endpoints: anthropometric measurements, fasting blood glucose, insulin, lipid profile, CK-18 M30, MPO levels, NAFLD fibrosis score, and 2-hour blood glucose value in oral glucose tolerance test (OGTT). | It has limitations including a single-center design, small sample size, potential subjective bias in efficacy assessment due to the open-label design, and failure to reach the Maximum Tolerated Dose (MTD) (withdrawal due to capsule burden). | | It confirms that purified anthocyanins can improve liver injury markers and insulin resistance in patients with non-alcoholic fatty liver disease (NAFLD), providing clinical evidence for their use as a non-pharmacological therapy for the early intervention of NAFLD. | [S50] | ClinicalTrials.gov: NCT01940263 |
|  | *Hesperidin*  flavanones | MAFLD | 70 cases | Exploratory, randomized, controlled clinical trial, single-center, double-blind design | Primary endpoint: changes in hepatic steatosis assessed by liver-controlled attenuation parameter.Secondary endpoints: degree of liver fibrosis, liver function indicators, anthropometric measurements, lipid profile, blood glucose, insulin resistance index, and inflammatory markers. | The randomized design reduces selection bias, and the blinding of outcome assessors lowers subjective bias. The core outcome indicators are objective test data, leading to a low risk of bias. However, there are limitations, such as a single-center design, small sample size, short intervention period (4 weeks), and the absence of liver biopsy to verify histological changes. | | It provides a simple and feasible dietary intervention plan for metabolic-associated steatotic liver disease (MASLD). It is confirmed that consuming 400 grams of oranges per day can reduce the prevalence of hepatic steatosis and increase vitamin intake, which provides a basis for daily dietary guidance for MASLD patients and is suitable as a first-line intervention for mild MASLD. | [S51] | ClinicalTrials.gov: NCT05558592 |
|  | *Citrus Bergamia*  Flavanones | MAFLD | 94 cases | Randomized, double-blind, placebo-controlled clinical trial | Primary endpoint: change in the IBS-SSS score. Secondary endpoints: IBS-QOL score and total score VAS. | Low bias, the factorial design can evaluate interaction effects. However, the sample size is small, and subjective symptom scores rely on patient reporting, which may introduce bias. | | It provides a new option for the management of MAFLD complicated with hyperuricemia. It is confirmed that the nutritional preparation containing bergamot polyphenols and cynara cardunculus extract can reduce serum uric acid and body weight, offering clinical evidence for the synergistic improvement of metabolic abnormalities in MAFLD patients, and is suitable for MAFLD patients related to metabolic syndrome. | [S52] | ISRCTN: ISRCTN12833814 |
|  | EGCG  flavonoids | MAFLD | 102 cases | Single-center, randomized, double-blind, parallel-controlled, placebo-controlled clinical trial | Primary endpoint: change in liver fat content detected by transient elastography. Secondary endpoints: body weight, BMI, lipid profile, liver function indicators, insulin resistance index, and inflammatory factors. | Low bias, the crossover design reduces individual differences, but only healthy women were included, limiting the extrapolation of results. | | As an exploratory randomized controlled trial (without clear traditional clinical staging), it confirms that the combined supplement of bergamot and wild artichoke extracts can improve hepatic steatosis in patients with NAFLD, providing a safe and effective non-pharmacological intervention option for NAFLD patients over 50 years old. | [S53] | ISRCTN: ISRCTN12833814, |
|  | Dihydromyricetin  flavanols | MAFLD | 60 cases | Randomized, double-blind, placebo-controlled clinical trial | The primary endpoints are blood glucose and lipid metabolism indicators, and liver enzyme levels. The secondary endpoints include inflammatory factors (TNF-α, FGF21) and insulin resistance (HOMA-IR). | There is no significant bias. Randomization and blinding have reduced selection and measurement biases. However, the diagnosis of MAFLD relies on ultrasound (not the biopsy gold standard), which may lead to diagnostic bias. | | It provides a safe and effective adjuvant treatment option for MAFLD. It is confirmed that dihydromyricetin (DHM) can improve liver enzymes, regulate glucose and lipid metabolism, and reduce inflammation with good tolerability. This provides a basis for MAFLD drug development and clinical medication, and is particularly suitable for patients in need of improving insulin resistance. | [S54] | ChiCTR: ChiCTR-TRC-12002377 |
|  | *Isoflavonoides sojae*  isoflavonoid | MAFLD | 50 cases | Double-blind, placebo-controlled, randomized parallel clinical trial | The primary endpoint is the improvement in hepatic steatosis grading at 12 weeks. The safety endpoint is the absence of adverse events. | Overall, the study has relatively low robustness. Potential limitations include its single-center design, small sample size, failure to detect serum soy isoflavone concentrations, and short follow-up period (12 weeks), which restrict the extrapolation of results. | | It improves hepatic steatosis and related liver function indicators in MAFLD patients, regulates metabolic markers, and has the advantages of safety and accessibility. It demonstrates the potential for clinical translation as part of the comprehensive management of MAFLD. However, further research is still needed on long-term application data, specific mechanisms of action, and applicability in special populations. | [S55] | ClinicalTrials.gov: NCT06101433 |
|  |  | Advanced pancreatic cancer (locally advanced or metastatic) | 20 cases | Single-arm, open-label clinical trial | The primary endpoint is the 6-month survival rate, and the secondary endpoints include median survival time, progression-free survival, objective response rate, and toxic reactions. | The single-arm design lacks a control group, and the limited sample size coupled with potential selection bias may affect the reliability of the results. | | The combination of soy isoflavones (genistein) with gemcitabine and erlotinib shows good safety in the treatment of advanced pancreatic cancer, though it does not improve patient survival. This provides a reference for the subsequent optimization of combination therapy regimens targeting the Akt/NF-*κ*B pathway and the screening of patient subgroups. | [S56] | ClinicalTrials.gov: NCT00461708 |
|  |  | Irritable Bowel Syndrome （IBS）in Women | 125 cases | Randomized, double-blind, placebo-controlled clinical trial | The primary endpoints are changes in plasma TNF-*α*, leukocyte NF-κB, plasma total antioxidant capacity (TAC), and fecal serine protease activity after 6 weeks of intervention. | The randomized double-blind design reduces selection bias and measurement bias. However, it does not analyze different IBS subtypes, and the lack of intestinal tissue biopsy data may result in certain limitations. | | It is confirmed that supplementation with soy isoflavones and cholecalciferol (vitamin D), either alone or in combination, can safely reduce inflammatory indicators and intestinal permeability in female patients with IBS, providing an effective non-pharmacological intervention regimen for clinical practice. | [S57] | ClinicalTrials.gov: NCT02026518 |
|  | Polyphenols from natural food sources  flavonoids | Increased intestinal permeability (intervention effect in older adults aged ≥ 60 years) | 51 cases | Single-center, randomized, controlled, crossover clinical trial | The primary endpoints are changes in intestinal permeability and inflammation-related biomarkers such as calprotectin (in serum and feces), ZO-1, and occludin; the secondary endpoint is the correlation clustering among biomarkers. | The crossover design reduces the impact of individual differences, and randomization lowers selection bias. However, there is a temporal effect (e.g., for VEC indicators), and it is not clearly stated whether researchers were fully blinded, which may introduce slight measurement bias. | | It is confirmed that a polyphenol-rich diet can reduce serum and fecal calprotectin levels in the elderly with increased intestinal permeability. This provides empirical support for improving intestinal permeability-related inflammation in the elderly and promoting healthy aging through dietary intervention. | [S58] | ISRCTN: ISRCTN10214981 |
|  | Chenpi enzyme (with ingredients including *Citrus reticulata* and *Crataegus pinnatifida*)  flavanones, flavonols | Dyslipidemia | 72 cases | Single-center, randomized, double-blind, parallel-controlled pilot clinical trial | The primary endpoints are changes in body weight and serum triglyceride (TG) levels. The secondary endpoints include physical parameters such as BMI, waist circumference, and hip circumference, as well as safety indicators like liver and kidney function. The exploratory endpoints are changes in serum metabolite profiles and intestinal microbiota structure. | The randomized double-blind design reduces selection bias and measurement bias. However, the small sample size and single-center nature of the study may lead to selection bias and random errors. Additionally, the lack of long-term follow-up data limits the extrapolation of the results. | | It is confirmed that Chenpi Jiaosu (CPJS, tangerine peel enzyme) is safe and effective for people with dyslipidemia. It can reduce triglyceride levels, body mass index (BMI), and hip circumference, and exerts its effects by regulating lipid/amino acid metabolism and intestinal microbiota. This provides a safe and feasible natural adjuvant intervention regimen for dyslipidemia. | [S59] | ChiCTR: ChiCTR2000033062 |
|  | *Hesperidinum*  flavanones | MAFLD | 100 cases | Multicenter, open-label, randomized controlled clinical trial | The primary endpoint is the reduction of hepatic steatosis. The secondary endpoints include anthropometric indicators, glycolipid metabolism parameters, inflammatory markers, and liver fibrosis-related indicators. | The open-label design may introduce measurement bias and subjective bias. It does not adopt intention-to-treat (ITT) analysis, and there are inter-group differences in AST and TNF-*α* levels at baseline, which may affect the objectivity of the results. | | It is confirmed that supplementation with flaxseed, hesperidin alone or in combination can safely improve glycolipid metabolism, reduce inflammation and hepatic steatosis in patients with MAFLD. Moreover, the combination of the two has a synergistic effect on improving blood glucose and insulin resistance, providing a feasible non-pharmacological adjuvant intervention regimen for MAFLD. | [S60] | ClinicalTrials.gov: NCT03734510 |
|  | Oligomeric proanthocyanidins  flavones | Diarrhea-predominant irritable bowel syndrome (D-IBS) | 245 cases | Multicenter, randomized, double-blind, placebo-controlled, exploratory clinical trial | The primary endpoint is the proportion of responders with improved stool consistency. The secondary endpoints include defecation frequency, abdominal pain/discomfort-free days, urgency control rate, and adequate symptom relief rate, among others. | The double-blind randomized design effectively reduces selection bias and measurement bias. However, the baseline stool consistency being close to the normal range may lead to a floor effect, and the lack of stratified analysis by gender for prespecified hypotheses may affect the interpretation of results. | | It is confirmed that although crofelemer did not improve defecation function-related indicators in patients with diarrhea-predominant irritable bowel syndrome (D-IBS), the dose of 500mg twice daily significantly increased the number of pain and discomfort relief days in female patients, with good tolerability. This provides a potential direction for pain management in female D-IBS patients and lays a foundation for further research on its use as a visceral analgesic. | [S61] | IRCT: IRCT20131125015536N12 |
|  | *Glucosyl hesperidin*  flavanones | Primary biliary cholangitis | 110 cases | Multicenter, open-label, randomized controlled exploratory clinical study | The primary endpoint is the rate of change in serum gamma-glutamyl transferase (GGT) level at 24 weeks; the secondary endpoints are the rates of change in hepatobiliary enzyme levels and Nrf2-related protein expression levels at 8, 16, and 24 weeks; the exploratory endpoints are changes in indicators such as inflammatory factors. | The open-label design may lead to measurement bias and subjective assessment bias. Although stratified randomization is used to balance baseline factors, the lack of a placebo control makes it impossible to rule out interference from the natural disease course or concurrent medication (ursodeoxycholic acid). | | This study aims to evaluate the adjuvant efficacy of glucosyl hesperidin combined with ursodeoxycholic acid (UDCA) in the treatment of primary biliary cholangitis (PBC). If proven effective, it can provide a safe complementary treatment regimen for PBC and also offer a reference for the treatment of inflammatory diseases related to oxidative stress. | [S62] | jRCTs: jRCTs051210210 |
|  | Jian-Wei-Qu-Tong Pills (with ingredients including *Glycyrrhiza* and *Taraxacum mongolicum*)  isoflavones, flavonols | Chronic non-atrophic gastritis（CNG) | 240 cases | Multicenter, randomized, double-blind, placebo-controlled clinical trial | The primary endpoint is the eradication rate of epigastric pain VAS score decreases to 0 and no longer rises). The secondary endpoints include changes in endoscopic and histopathological indicators, helicobacter pylori eradication rate, TCM symptom score, and the score of the patient-reported outcomes measurement information system (PROMIS) for gastrointestinal diseases. | The double-blind randomized design effectively reduces selection bias and measurement bias, and stratified randomization balances center-related factors. However, the placebo and the trial drug are not completely consistent in color and odor, which may pose a potential risk of unblinding; in addition, relying on subjective symptom scores may introduce measurement bias. | | It provides a standardized clinical research protocol for TCM (Jianwei Qutong Wan) with effects of invigorating the spleen, dispelling dampness, activating blood circulation and relieving pain, for patients with chronic non-atrophic gastritis of spleen-stomach qi deficiency combined with dampness-heat and blood stasis syndrome. It explores potential new therapies for such patients who have poor response to Western medicine treatment or recurrent symptoms, and at the same time provides high-quality evidence-based basis for the phase III clinical trial and clinical application of this TCM. | [S63] | ChiCTR: ChiCTRTRC14004088 |
|  | *Crofelemer*  anthocyanin | Diarrhea-predominant irritable bowel syndrome in women(D-IBS） | 240 cases | Multicenter, randomized, double-blind, placebo-controlled clinical trial | The primary endpoint is the overall change in the percentage of days with resolved abdominal pain/discomfort within 12 weeks; the secondary endpoints include stool consistency, defecation frequency, urgency, and the rate of adequate symptom relief; the post-hoc analysis endpoints are the monthly pain, stool consistency, and combined response rates as defined by the FDA. | The double-blind randomized design effectively reduces selection bias and measurement bias. However, the relatively strict definition of the primary endpoint (complete resolution of pain/discomfort) may affect the results, and the high placebo effect inherent in IBS itself may weaken the difference between the intervention group and the control group. | | It is confirmed that crofelemer at a dose of 125mg twice daily has good safety in patients with diarrhea-predominant IBS-D. Although it did not improve the primary endpoint, it showed a significantly higher monthly abdominal pain response rate (as defined by the FDA) in the post-hoc analysis. This provides a potential new non-opioid, non-constipating option for the treatment of abdominal pain in female patients with IBS-D. | [S64] | ClinicalTrials.gov: NCT00461526 |
|  | Elian Granules (with ingredients including *Rhizoma Coptidis, Radix Codonopsis* ang *Salvia miltiorrhiza Bunge)*  flavones,flavonols | Chronic atrophic gastritis | 240 cases | Randomized, double-blind, placebo-controlled, multicenter clinical trial | The primary endpoint is the histological change of gastric mucosa based on the OLGA/OLGIM staging system after 24 weeks. The secondary endpoints include the kimura-takemoto endoscopic classification, dyspepsia symptom score, and the score of the SF-12 quality of life scale. | The double-blind randomized design effectively reduces selection bias and measurement bias, and center stratification balances regional differences. However, the placebo and the trial drug are not completely consistent in color and odor, which may pose a potential risk of unblinding. Additionally, relying on subjective symptom scores may introduce slight measurement bias. | | It provides a standardized clinical research protocol for TCM with heat-clearing, blood-activating, and spleen-invigorating effects (Lian Keli) for patients with chronic atrophic gastritis (including intestinal metaplasia). It explores new potential therapies for reversing or stabilizing the condition in such patients who lack specific treatments and are in the pre-gastric cancer lesion stage. Meanwhile, it offers high-quality evidence-based basis for the phase III clinical trial and clinical application of this TCM. | [S65] | ChiCTR: ChiCTR2000003929 |
|  | *~~Eucommia ulmoides~~*  ~~flavones~~ | ~~Atrophic gastritis~~ | ~~20 cases~~ | ~~Single-center, adopting a randomized, double-blind, parallel, placebo-controlled design~~ | ~~The primary endpoint is the improvement rate of pathological grading of gastric mucosal atrophy at 18 weeks; the secondary endpoints include other pathological changes of gastric mucosa, changes in the grading of upper abdominal distension/pain, effectiveness of TCM syndrome, endoscopic evaluation results of gastric mucosal inflammation, and Helicobacter pylori eradication rate.~~ | ~~There are potential risks including insufficient statistical power due to an excessively small sample size, limited extrapolation of results caused by the single-center design, and potential bias in the assessment of gastric mucosal pathological grading which relies on subjective judgment.~~ | | ~~As a phase II exploratory study with a small sample size, its results mainly provide a preliminary direction for subsequent larger-sample, multi-center confirmatory studies. In particular, it can supplement the early evidence of TCM in the treatment of chronic atrophic gastritis of qi-yin deficiency type. If subsequent studies confirm its effectiveness, it is expected to add a new option to TCM treatment for this disease. However, the current results do not yet have sufficient reliability for direct clinical translation.~~ | ~~[S66]~~ | ~~ClinicalTrials.gov: NCT05209633~~ |
|  | *Curcumin*  flavones | Chronic atrophic gastritis or gastrointestinal intraepithelial neoplasia | 50 cases | Randomized, double-blind, parallel-controlled design | The primary endpoint is the absolute change in the level of IL-1*β* cytokine in gastric mucosa from baseline to 6 months; the secondary endpoints include the change in gastric mucosa histological score at 6 months, changes in the levels of other cytokines, gastric mucosa DNA damage, and the exploration of the association between baseline pro-inflammatory cytokine genotypes and the aforementioned outcomes. | The small sample size may lead to insufficient statistical power, making it difficult to accurately detect the intervention effect; the consistent process for multi-rater blind evaluation of subjective indicators such as gastric mucosa histological score is not clearly defined, resulting in potential subjective assessment bias; the study is conducted at only 2 geographically concentrated sites, which may limit the applicability of the results when extrapolated to populations in other regions/ethnicities. | | As a phase II trial with published results, it provides preliminary evidence of safety and biomarker (e.g., IL-1*β*) response for curcumin in gastric cancer chemoprevention. It can support the subsequent launch of larger-scale, multi-regional phase III clinical trials to further verify its effectiveness in preventing gastric cancer. If the results are positive, it is expected to promote curcumin as a clinical drug for gastric cancer prevention in high-risk populations such as those with chronic atrophic gastritis, filling the gap in chemopreventive methods in this field. | [S66] | ClinicalTrials.gov: NCT02782949 |
|  | Houtou Jianweiling Tablet (with ingredients including *Radix Paeoniae Alba* and *Rhizoma Cyperi*)  flavones, flavonols | Chronic atrophic gastritis | 240 cases | Randomized, double-blind, placebo-controlled, non-inferiority design | The primary endpoints are the improvement rate of major clinical symptoms and the physician global assessment (PGA) during the 4-week trial period. The secondary endpoints include laboratory tests such as liver and kidney function before enrollment and within 5 days after treatment, vital sign measurements within 4 weeks, helicobacter pylori stool antigen test within 5 days after treatment, and assessment of the QT interval on electrocardiogram (ECG) before enrollment and within 5 days after treatment. | A single-center design limits the extrapolation of results to populations in other regions/ethnicities. The consistent calibration process for multi-rater blind evaluation of subjective indicators such as stomach pain VAS score and stomach distension score is not clearly defined, leading to potential subjective assessment bias. There is no mention of whether a DMC was established, resulting in insufficient clear information on the quality monitoring of the trial process. | | A sample size of 240 has more statistical reference significance compared to small and medium-sized studies. The non-inferiority design allows for direct comparison of efficacy with omeprazole, a commonly used clinical drug. Additionally, the study has been completed and relevant papers have been published, which can provide relatively reliable evidence for the clinical application of Houtou Jianweiling tablets in the treatment of chronic non-atrophic gastritis. Especially in regions where traditional Chinese medicine treatments are preferred, it is expected to promote the tablets as an alternative therapeutic drug for this disease. | [S67] | ClinicalTrials.gov: NCT04672018 |
|  | *Crocus sativus*  flavones | Ulcerative colitis | 60 cases | Parallel, randomized, double-blind clinical design | Primary endpoints: changes in the "simple clinical colitis activity index" score, fecal calprotectin level, serum C-reactive protein (CRP) level, and serum erythrocyte sedimentation rate (ESR) level at 4 weeks and 8 weeks, changes in health-related quality of life score and Beck Anxiety Inventory score at 8 weeks, 24 weeks, and 48 weeks. | The low risks lie in the use of sequentially numbered, sealed, and opaque envelopes to achieve allocation concealment, and the adoption of a double-blind design, which effectively reduces selection bias and subjective assessment bias. The potential risks include the failure to mention a multicenter design (which limits the extrapolation of results), a small sample size (which may affect statistical power), and the lack of consistency verification for indicator detection (which raises doubts about data reliability). | | As a phase 2 study, it provides dose-exploration data and preliminary evidence of efficacy for saffron as a complementary therapy for ulcerative colitis (UC). If proven effective, it can offer a safer alternative/complementary treatment option for UC patients sensitive to the side effects of synthetic drugs, while promoting the application of plant-derived drugs in the treatment of inflammatory bowel disease (IBD). | [S68] | ClinicalTrials.gov: NCT05117749 |
| Phase Ⅱ/Ⅲ | EGCG  flavanols | MAFLD | 20 cases | Exploratory randomized controlled clinical trial | The primary endpoints are the changes in hepatic fat content and serum dipeptidyl peptidase-4 (DPP-4) level after 24 weeks. The secondary endpoints include anthropometric indicators, glycolipid metabolism parameters, liver and kidney function, and the expression levels of inflammation-related genes/proteins. | The clinical part adopted an open-label design without a placebo control, which may introduce measurement bias and subjective bias. A small sample size is prone to random errors, but the combination with basic experimental verification has compensated for the limitations of the clinical study to a certain extent. | | It is confirmed that EGCG can reduce hepatic lipid accumulation, improve lipid metabolism and alleviate inflammation by inhibiting the expression and activity of DPP4, thereby providing a safe and effective potential treatment option for MAFLD which lacks specific drugs. | [S69] | ChiCTR: ChiCTR2300076741 |
| Phase III | Aurantii fructus immaturus flavonoid  flavanones | Functional dyspepsia (FD) | 294 cases | Multicenter, double-blind, double-dummy, positive-controlled clinical trial | The primary endpoint is the disappearance rate of 4 core symptoms after 4 weeks of treatment; the secondary endpoints include the disappearance rate of individual symptoms, gastric emptying function, and the disappearance rate of symptoms 4 weeks after drug withdrawal. | The double-blind, double-dummy design effectively reduces selection bias and measurement bias, and stratified randomization balances center-related factors. However, relying on subjective assessment of symptoms may introduce slight measurement bias, and the failure to adopt the Rome IV diagnostic criteria may affect the extrapolation of results. | | The clinical translation value of this study lies in confirming that the 4-week efficacy of Zhishi Huangtong tablets (*Fructus Aurantii* flavonoid tablets) in treating functional dyspepsia is comparable to that of domperidone. Moreover, it has more lasting symptom relief effects after drug withdrawal and better safety (with a lower incidence of adverse reactions), providing a safer alternative treatment option with durable efficacy for patients with functional dyspepsia. | [S70] | ChiCTR: ChiCTR2400083076 |
|  | Total *anthocyanins*  anthocyanins | MAFLD | 80 cases | Double-blind, randomized, placebo-controlled clinical trial | The primary endpoints are the changes in liver function indices, degree of hepatic steatosis and fibrosis, as well as serum levels of TNF-*α*, MDA, and adiponectin after 12 weeks. The secondary endpoints are the changes in glycolipid metabolism parameters and insulin resistance index. | The double-blind randomized design effectively reduces selection bias and measurement bias, and balances baseline factors through stratification by age, gender, and other variables. However, its sample size is relatively limited and recruitment relies on a single center, which may affect the extrapolation of the results. | | It provides a potential adjuvant treatment regimen based on *Cornus* officinalis extract for patients with MAFLD who lack specific treatments. By targeting the improvement of liver function and reducing inflammation and oxidative stress, it offers new research directions and evidence-based basis for non-pharmacological interventions in MAFLD. | [S71] | IRCT: IRCT20180419039359N1 |
|  | *Proanthocyanidins*  anthocyanins | MAFLD | 50 cases | Double-blind, randomized, placebo-controlled clinical trial | The primary endpoint is the change in superoxide dismutase levels. Secondary endpoints include oxidative stress markers, inflammatory cytokines, and quality of life assessed by the SF-36 questionnaire. Safety endpoints involve monitoring adverse events. | The double-blind randomized design effectively minimized selection and measurement biases, while stratified randomization balanced baseline factors. However, the small sample size, single-center recruitment, and lack of assessment for core indicators of hepatic steatosis and fibrosis may limit the generalizability and comprehensiveness of the findings. | | It can safely improve oxidative stress levels, reduce inflammation, and enhance quality of life in MAFLD patients, offering a natural intervention option with both antioxidant and anti-inflammatory effects for the adjunctive treatment of MAFLD. | 202 | IRCT: IRCT20190731044392N2 |
|  | *Quercetin*  flavonols | MAFLD | 41 cases | Randomized, double-blind, placebo-controlled crossover clinical trial | The primary endpoint is the change in hepatic fat content assessed by MRI-PDFF. Secondary endpoints include anthropometric measurements, glucose and lipid metabolism parameters, hepatic and renal function, and insulin resistance indices. Safety endpoints are blood counts. | Cross-over design minimizes interference from individual variability, while double-blind design reduces measurement bias. However, the relatively limited sample size and exclusion of patients with severe liver fibrosis may affect the generalizability of results. Additionally, gender imbalance could introduce potential confounding factors. | | The clinical translational value of this study lies in confirming that a 12-week intervention with 500mg of quercetin daily safely reduces hepatic lipid accumulation in patients with MAFLD, potentially through modest weight loss. This provides a natural, readily accessible non-pharmaceutical adjunctive treatment option for MAFLD. | [S72] | ChiCTR: ChiCTR2100047904 |
|  | Qirui Weishu capsule (with ingredients including *Panax notoginseng*)  flavonols | Chronic non-atrophic gastritis | 480 cases | Multicenter, randomized, double-blind, positive drug-controlled clinical trial | The primary endpoint is the rate of resolution of epigastric pain after 4 weeks of treatment. Secondary endpoints include the rate of resolution of epigastric fullness, the overall effective rate of Traditional Chinese Medicine syndromes, improvement in mucosal erosion and histopathological inflammation observed during gastroscopy, and the Helicobacter pylori eradication rate. Safety endpoints include laboratory parameters such as complete blood count, liver and kidney function, and electrocardiogram. | The double-blind randomized design effectively minimizes selection bias and measurement bias. Central randomization balances regional differences, but reliance on subjective symptom ratings may introduce minor measurement bias. Furthermore, the absence of a placebo control precludes complete exclusion of potential interference from positive drugs. | | Treatment for chronic non-atrophic gastritis with damp-heat and blood stasis obstruction is safe and effective. It demonstrates superior efficacy to Sanjiu Weitai capsules in alleviating epigastric pain and improving traditional Chinese medicine syndromes, offering an optimal herbal treatment option for such patients. | [S73] | ChiCTR: CTR20180969 |
|  | Epigallocatechin gallate  flavanols | Female central obesity | 115 cases | Single-center, randomized, double-blind, placebo-controlled clinical trial | Primary endpoints include changes in body weight, BMI, and waist circumference. Secondary endpoints encompass lipid profiles, insulin resistance indices, and levels of obesity-related hormones such as leptin and adiponectin. Safety endpoints encompass liver and kidney function, electrocardiograms, and adverse events. | The double-blind randomized design effectively minimized selection and measurement biases. However, single-center recruitment, limited sample size, and inclusion of only female participants may compromise the generalizability of the findings. Potential differences in baseline HbA1c and adiponectin levels between the placebo and treatment groups could introduce minor confounding biases. | | High-dose EGCG supplementation over 12 weeks safely reduces body weight, waist circumference, total cholesterol, and low-density lipoprotein cholesterol in women with central obesity. This effect may be mediated by suppressing ghrelin secretion and increasing adiponectin levels, offering an effective natural adjunct for weight management in obesity. | [S74] | ClinicalTrials.gov: NCT02147041 |

Reference

1. Hodek, P., Hanustiak, P., Krízková, J., Mikelova, R., Krízková, S., Stiborová, M., et al. Toxicological aspects of flavonoid interaction with biomacromolecules. Neuro Endocrinol. Lett (2006) 27 Suppl 2, 14-17.
2. Canada, A. T., Watkins, W. D., Nguyen, T. D. The toxicity of flavonoids to guinea pig enterocytes. Toxicol. Appl. Pharmacol (1989) 99(2), 357-361. <https://doi.org/10.1016/0041-008x(89)90018-5>
3. Deng, H., Zhao, Y., He, Y., Teng, H., Chen, L. Unveiling the Dark Side of Flavonoid: Rutin Provokes Hepatotoxicity in Low-Dose 2-Amino-3-methylimidazo [4,5-f] Quinoline-Exposed Mice via Regulating Gut Microbiota and Liver Metabolism. [J. Agric. Food Chem](https://www.x-mol.com/paper/journal/153?r_detail=1883600125099073536) (2025) 73(7), 4253-69. <https://doi.org/10.1021/acs.jafc.4c07330>
4. Li, X., Li, Y., Chen, S., Guo, L., Mei, N. Potential anticancer effects and toxicity of flavones luteolin and apigenin in vivo. Journal of Environmental Science and Health, Part C (2025) 1-37. Advance online publication. <https://doi.org/10.1080/26896583.2025.2527437>
5. Lin, H., Guo, X., Zhang, S., Dial, S. L., Guo, L., Manjanatha, M. G., et al. Mechanistic evaluation of Ginkgo biloba leaf extract-induced genotoxicity in L5178Y cells. Toxicol. Sci (2014) 139(2), 338-349. <https://doi.org/10.1093/toxsci/kfu037>
6. Kulling, S. E., Rosenberg, B., Jacobs, E., Metzler, M. The phytoestrogens coumoestrol and genistein induce structural chromosomal aberrations in cultured human peripheral blood lymphocytes. Arch. Toxicol (1999) 73(1), 50-54. <https://doi.org/10.1007/s002040050585>
7. da Silva, J., Herrmann, S. M, Heuser, V., Peres, W., Possa Marroni, N., González-Gallego, J., et al. Evaluation of the genotoxic effect of rutin and quercetin by comet assay and micronucleus test. Food Chem. Toxicol (2002) 40(7), 941-7. <https://doi.org/10.1016/s0278-6915(02)00015-7>
8. Laires, A., Gaspar, J., Borba, H., Proença, M., Monteiro, M., Rueff, J. Genotoxicity of nitrosated red wine and of the nitrosatable phenolic compounds present in wine: tyramine, quercetin and malvidine-3-glucoside. Food Chem. Toxicol (1993) 31(12), 989-994. <https://doi.org/10.1016/0278-6915(93)90008-m>
9. TANG Z, ZHANG Q. The Potential Toxic Side Effects of Flavonoids. BIOCELL (2022) 46(2), 357-366. <https://doi.org/10.32604/biocell.2022.015958>
10. Galati, G., O'Brien P,. J. Potential toxicity of flavonoids and other dietary phenolics: significance for their chemopreventive and anticancer properties. Free Radical Bio. Med (2004) 37(3), 287-303. <https://doi.org/10.1016/j.freeradbiomed.2004.04.034>
11. Soleimani, V., Delghandi, P. S, Moallem, S. A, Karimi, G. Safety and toxicity of silymarin, the major constituent of milk thistle extract: An updated review. Phytother. Res (2019) 33(6), 1627-38. <https://doi.org/10.1002/ptr.6361>
12. Morino, K., Matsukara, N., Kawachi, T., Ohgaki, H., Sugimura, T., Hirono, I. Carcinogenicity test of quercetin and rutin in golden hamsters by oral administration. Carcinogenesis (1982) 3(1), 93-97. <https://doi.org/10.1093/carcin/3.1.93>
13. Sahu, S. C, Gray, G. C. Pro-oxidant activity of flavonoids: effects on glutathione and glutathione S-transferase in isolated rat liver nuclei. [Cancer Lett](https://www.x-mol.com/paper/journal/724?r_detail=1213037313256525837) (1996) 104(2), 193-6. <https://doi.org/10.1016/0304-3835(96)04251-6>
14. Yao, W., Cheng, J., Kandhare, A. D, Mukherjee-Kandhare, A. A, Bodhankar, S. L, Lu, G. Toxicological evaluation of a flavonoid, chrysin: morphological, behavioral, biochemical and histopathological assessments in rats. [Drug Chem. Toxicol](https://www.x-mol.com/paper/journal/26525?r_detail=1213063374799310848) (2021) 44(6), 601-612. <https://doi.org/10.1080/01480545.2019.1687510>
15. Fathima, A., Rao, J. R. Selective toxicity of Catechin-a natural flavonoid towards bacteria. [Appl. Microbiol. Biotechnol](https://www.x-mol.com/paper/journal/2974?r_detail=1212906175635202076) (2016) 100(14), 6395-6402. <https://doi.org/10.1007/s00253-016-7492-x>
16. Goodarzi, S., Tabatabaei, M. J, Mohammad, Jafari R., Shemirani, F., Tavakoli, S., Mofasseri, M., et al. Cuminum cyminum fruits as source of luteolin- 7-O-glucoside, potent cytotoxic flavonoid against breast cancer cell lines. [Nat. Prod. Res](https://www.x-mol.com/paper/journal/1763?r_detail=1213009916528365593) (2020) 34(11), 1602-06. <https://doi.org/10.1080/14786419.2018.1519824>
17. Raafat, K., Breitinger, U., Mahran, L., Ayoub, N., Breitinger, H. G. Synergistic inhibition of glycinergic transmission in vitro and in vivo by flavonoids and strychnine. Toxicol. Sci (2010) 118(1), 171-182. <https://doi.org/10.1093/toxsci/kfq245>
18. Hong, X. C., Liang, Q. L., Luo, X. B., Hu, K. H., Yang, H. X., Ou, W. T., et al. Clinical study of XiangShaLiuJunZi decoction combined with S-1 as maintenance therapy for stage III or IV gastric carcinoma and colorectal carcinoma. Medicine (Baltimore) (2020). 99(19):e20081. https://doi.org/10.1097/MD.0000000000020081
19. Lv, L., Wang, F. Y., Ma, X. X., Li, Z. H., Huang, S. P., Shi, Z. H., et al. Efficacy and safety of Xiangsha Liujunzi granules for functional dyspepsia: a multi-center randomized double-blind placebo-controlled clinical study. World J Gastroenterol (2017). 23(30):5589-601. https://doi.org/ 10.3748/wjg.v23.i30.5589
20. Shih, Y. S., Tsai, C. H., Li, T. C., Lai, H. C., Wang, K. T., Liao, W. L., et al. The effect of Xiang-Sha-Liu-Jun-Zi tang (XSLJZT) on irritable bowel syndrome: a randomized, double-blind, placebo-controlled trial. J. Ethnopharmacol (2019). 238:111889. <https://doi.org/10.1016/j.jep.2019.111889>
21. Wang, Y., Jia, Y., Liu, X., Yang, K., Lin, Y., Shao, Q., et al. Effect of Chaihu-Shugan-San on functional dyspepsia and gut microbiota: a randomized, double-blind, placebo-controlled trial. J. Ethnopharmacol (2024). 322:117659. <https://doi.org/10.1016/j.jep.2023.117659>
22. Zou, T. H., Gao, Q. Y., Liu, S., Li, Y. Q., Meng, X. J., Zhang, G. X., et al. Effectiveness and safety of Moluodan in the treatment of precancerous lesions of gastric cancer: a randomized clinical trial. [J. Dig. Dis.](https://www.x-mol.com/paper/journal/5276?r_detail=1762661145554685952) (2024). 25(1):27-35. <https://doi.org/10.1111/1751-2980.13251>
23. Tang, X. D., Zhou, L. Y., Zhang, S. T., Xu, Y. Q., Cui QC, Li L, et al. Randomized double-blind clinical trial of Moluodan for the treatment of chronic atrophic gastritis with dysplasia. Chin. J. Integr. Med (2016). 22(1):9-18. <https://doi.org/10.1007/s11655-015-2114-5>
24. Zhang, Z., Chen, L., Li, X., Cai, M., Yan, H., Sun, G. A. Quality control system of Chinese medicine preparation based on fingerprint identification technology, chemometrics and network pharmacology, using the Yixinshu capsule as an example. Talanta (2025). 292:128015. <https://doi.org/10.1016/j.talanta.2025.128015>
25. Wang, Z. J., Wang, X. H., Li, J., Zheng, S. H., Zhang, F. P., Hao, S. L., et al. The efficacy and safety of modified Gegenqinlian Fomular for advanced colorectal cancer (damp heat accumulation type): a multicenter randomized controlled trial. Medicine (Baltimore) (2021). 100(49):e27850. <https://doi.org/10.1097/MD.0000000000027850>
26. Zhang, S. S., Zhao, L. Q., Wang, H. B., Wu, B., Wang, C. J., Huang, S. P., et al. Efficacy of Gastrosis No.1 compound on functional dyspepsia of spleen and stomach deficiency-cold syndrome: a multi-center, double-blind, placebo-controlled clinical trial. Chin J Integr Med (2013). 19(7):498-504. <https://doi.org/10.1007/s11655-013-1503-x>
27. Zhang, S., Zhao, L., Wang, H., Wang, C., Huang, S., Shen, H., et al. Efficacy of modified LiuJunZi decoction on functional dyspepsia of spleen-deficiency and qi-stagnation syndrome: a randomized controlled trial. [BMC Complement. Altern. Med.](https://www.x-mol.com/paper/journal/1548?r_detail=1212934144709697539) (2013). 13:54. <https://doi.org/10.1186/1472-6882-13-54>
28. Xu, J., Wang, R., You, S., Zhang, L., Zheng, P., Ji, G., et al. Traditional Chinese medicine Lingguizhugan decoction treating non-alcoholic fatty liver disease with spleen-yang deficiency pattern: study protocol for a multicenter randomized controlled trial. Trials (2020). 21(1):512. <https://doi.org/10.1186/s13063-020-04362-7>
29. Xiao, H. M., Shi, M.J., Jiang, J. M., Cai, G. S., Xie, Y. B., Tian, G. J., et al. Efficacy and safety of AnluoHuaxian pills on chronic hepatitis B with normal or minimally elevated alanine transaminase and early liver fibrosis: a randomized controlled trial. J. Ethnopharmacol (2022). 293:115210. <https://doi.org/10.1016/j.jep.2022.115210>
30. Yao, C., Huang, G., Wang, M., Xia, M., Yao, F., Niu, D., et al. Chinese herbal medicine formula Jieduhuayu granules improves cognitive and neurophysiological functions in patients with cirrhosis who have minimal hepatic encephalopathy: a randomized controlled trial. Complement Ther. Med (2014). 22(6):977-85. <https://doi.org/10.1016/j.ctim.2014.10.005>
31. Zhong, L. L., Cheng, C. W., Chan, Y., Chan, K.H., Lam, T. W., Chen, X. R., et al. Chinese herbal medicine (Ma Zi Ren Wan) for functional constipation: study protocol for a prospective, double-blinded, double-dummy, randomized controlled trial. Trials (2013). 14:366. <https://doi.org/10.1186/1745-6215-14-366>
32. Wu, Z., Fu, X., Jing, H., Huang, W., Li, X., Xiao, C., et al. Herbal medicine for the prevention of chemotherapy-induced nausea and vomiting in patients with advanced colorectal cancer: a prospective randomized controlled trial. J. Ethnopharmacol (2024). 325:117853. <https://doi.org/10.1016/j.jep.2024.117853>
33. Qiao, L., Wang, L. J., Wang, Y., Chen, Y., Zhang, H. L., Zhang, S. C. A randomized, double-blind, and placebo-controlled trial of Chinese herbal medicine in the treatment of childhood constipation. [Clin. Transl. Gastroen.](https://www.x-mol.com/paper/journal/882?r_detail=1391012052678103040) (2021). 12(5):e00345. <https://doi.org/10.14309/ctg.0000000000000345>
34. Wu, J., Ge, Y., Zhu, G., Gao, R., Zhu, X., Zhang, Y., et al. Combination of compound kushen injection with first-line treatment versus first-line treatment alone for advanced colorectal cancer: a study protocol for a multicenter, openlabel, randomized controlled trial. BMC Complement Med Ther (2024). 24(1):429. <https://doi.org/10.1186/s12906-024-04725-6>
35. Li, B. Y., Xi, Y., Liu, Y. P., Wang, D., Wang, C., Chen, C. G., et al. Effects of silybum marianum, pueraria lobate, combined with salvia miltiorrhiza tablets on non-alcoholic fatty liver disease in adults: a triple-blind, randomized, placebo-controlled clinical trial. Clin. Nutr. ESPEN (2024). 63:2-12. <https://doi.org/10.1016/j.clnesp.2024.06.003>
36. He, A. R., Smith, C. I., Cruz-Correa, M., et al. A Phase I dose-escalation study of Polyphenon E in liver cirrhosis: evaluation of safety and effect on liver γ-OHPdG levels. Cancer Prev. Res. (Phila) 2025;18(10):635-46. <https://doi.org/10.1158/1940-6207.CAPR-24-0526>
37. Schrieber, S. J., Hawke, R. L., Wen, Z., Smith, P. C., Reddy, K. R., Wahed, A. S., et al. Differences in the disposition of silymarin between patients with nonalcoholic fatty liver disease and chronic hepatitis C. Drug Metab. Dispos (2011). 39(12):2182-90. <https://doi.org/10.1124/dmd.111.040212>
38. Ponce Martínez, C., Murcia García, E., Pérez Sánchez, H., Milagro, F. I., Riezu-Boj, J. I., Ramos Molina, B.,et al. Effect of silibinin on human pancreatic lipase inhibition and gut microbiota in healthy volunteers: a randomized controlled trial. Int. J. Mol. Sci (2024) 25(23):12853. <https://doi.org/10.3390/ijms252312853>
39. Löhr, J. M., Karimi, M., Omazic, B., Kartalis. N., Verbeke. C. S., Berkenstam, A., et al. A phase I dose escalation trial of AXP107-11, a novel multi-component crystalline form of genistein, in combination with gemcitabine in chemotherapy-naive patients with unresectable pancreatic cancer. Pancreatology (2016). 16(4):640-5. <https://doi.org/10.1016/j.pan.2016.05.002>
40. Mostafa, H., Behrendt, I., Meroño, T., González-Domínguez, R., Fasshauer, M., Rudloff, S., et al. Plasma anthocyanins and their metabolites reduce in vitro migration of pancreatic cancer cells, PANC-1, in a FAK- and NF-kB dependent manner: results from the ATTACH-study a randomized, controlled, crossover trial in healthy subjects. Biomed. Pharmacother. (2023). 158:114076. <https://doi.org/10.1016/j.biopha.2022.114076>
41. ShuGuang Hospital. (2023). Study on Relevant Intervention for Gastric Diseases. <https://www.clinicaltrials.gov/study/NCT05908838?cond=Gastric%20diseases&page=4&rank=33> [Accessed November 3, 2025].
42. Xiyuan Hospital of China Academy of Chinese Medical Sciences. (2021). Study on Relevant Intervention for Gastric Diseases. https://www.clinicaltrials.gov/study/NCT03823248?cond=Gastric%20diseases&page=4&rank=37 [Accessed November 3, 2025].
43. Li, X., Xing, L., Zhang, Y., Xie, P., Zhu, W., Meng, X., et al. Phase II trial of epigallocatechin-3-gallate in acute radiation-induced esophagitis for esophagus cancer. J. Med. Food. (2020). 23(1):43-49. <https://doi.org/10.1089/jmf.2019.4445>
44. Zhao, H., Xie, P., Li, X., Zhu, W., Sun, X., Sun, X., et al. A prospective phase II trial of EGCG in treatment of acute radiation-induced esophagitis for stage III lung cancer. Radiother. Oncol (2015). 114(3):351-6. <https://doi.org/10.1016/j.radonc.2015.02.014>
45. Fernandes, R. C., Araújo, V. A., Giglio, B. M., Marini, A. C. B, Mota, J. F., Teixeira, K. S., et al. Acute pigallocatechin 3 gallate (EGCG) supplementation delays gastric emptying in healthy women: a randomized, double-blind, placebo-controlled crossover study. Nutrients (2018). 10(8):1122. <https://doi.org/10.3390/nu10081122>
46. Wah Kheong, C., Nik Mustapha, N. R., Mahadeva, S. A randomized trial of silymarin for the treatment of nonalcoholic steatohepatitis. Clin Gastroenterol Hepatol (2017). 15(12):1940-49.e8. <https://doi.org/10.1016/j.cgh.2017.04.016>
47. Kuipers, T., Oude Nijhuis, R. A. B., Schuitenmaker, J. M., Bredenoord, A. J. The clinical effect of benesco™ on reflux symptoms: a double-blind randomized placebo-controlled trial. Neurogastroenterol. Motil. (2023) 35(10):e14648. <https://doi.org/10.1111/nmo.14648>
48. Li, Z. X., Ma, J. L., Guo, Y., Liu, W. D., Li, M., Zhang, L. F., et al. Suppression of helicobacter pylori infection by daily cranberry intake: a double-blind, randomized, placebo-controlled trial. J. Gastroenterol Hepatol (2021). 36(4):927-35. <https://doi.org/10.1111/jgh.15212>
49. Biedermann, L., Doulberis, M., Schreiner, P., Nielsen, O. H., The, F. O., Brand, S., et al. Efficacy and safety of anthocyanin-rich extract in patients with ulcerative colitis: a randomized controlled trial. Nutrients (2024). 16(23):4197. <https://doi.org/10.3390/nu16234197>
50. Zhang, P. W., Chen, F. X., Li, D., Ling, W.H., Guo, H. H. A CONSORT-compliant, randomized, double-blind, placebo-controlled pilot trial of purified anthocyanin in patients with nonalcoholic fatty liver disease. Medicine (Baltimore) (2015). 94(20):e758. <https://doi.org/10.1097/MD.0000000000000758>
51. Notarnicola, M., Tutino, V., De Nunzio, V., Cisternino, A. M., Cofano, M., Donghia, R., et al. Daily orange consumption reduces hepatic steatosis prevalence in patients with metabolic dysfunction-associated steatotic liver disease: exploratory outcomes of a randomized Clinical trial. Nutrients (2024). 16(18):3191. <https://doi.org/10.3390/nu16183191>
52. Ferro, Y., Maurotti, S., Mazza, E., Pujia, R., Sciacqua, A., Musolino, V., et al. Citrus bergamia and cynara cardunculus reduce serum uric acid in individuals with non-alcoholic fatty liver disease. Medicina (2022). 58(12):1728. <https://doi.org/10.3390/medicina58121728>
53. Ferro, Y., Montalcini, T., Mazza, E., Foti, D., Angotti, E., Gliozzi, M., et al. Randomized clinical trial: bergamot citrus and wild cardoon reduce liver steatosis and body weight in non-diabetic Individuals aged over 50 years. Front. Endocrinol. (Lausanne) (2020). 11:494. <https://doi.org/10.3389/fendo.2020.00494>
54. Chen, S., Zhao, X., Wan, J., Ran, L., Qin, Y., Wang, X., et al. Dihydromyricetin improves glucose and lipid metabolism and exerts anti-inflammatory effects in nonalcoholic fatty liver disease: a randomized controlled trial. [Pharmacol.Res.](https://www.x-mol.com/paper/journal/111609?r_detail=1308378584732372992) (2015). 99:74-81. <https://doi.org/10.1016/j.phrs.2015.05.009>
55. Neshatbini, Tehrani, A., Hatami, B., Helli, B., Yari, Z., Daftari, G., Salehpour, A., et al. The effect of soy isoflavones on non-alcoholic fatty liver disease and the level of fibroblast growth factor-21 and fetuin A. [Sci. Rep.](https://www.x-mol.com/paper/journal/55?r_detail=1763716779129671680) (2024). 14(1):5134. <https://doi.org/10.1038/s41598-024-55747-6>
56. El-Rayes, B. F., Philip, P. A., Sarkar, F. H., Shields, A. F., Ferris, A. M., Hess, K., et al. A phase II study of isoflavones, erlotinib, and gemcitabine in advanced pancreatic cancer. Invest. New Drugs (2011). 29(4):694-9. <https://doi.org/10.1007/s10637-010-9386-6>
57. Jalili, M., Vahedi, H., Poustchi, H., Hekmatdoost, A. Soy isoflavones and cholecalciferol reduce inflammation, and gut permeability, without any effect on antioxidant capacity in irritable bowel syndrome: a randomized clinical trial. Clin. Nutr. ESPEN (2019). 34:50-54. <https://doi.org/10.1016/j.clnesp.2019.09.003>
58. Marino M, Del Bo' C, Martini D, Perna S, Porrini M, Cherubini A, et al. A (poly)phenol-rich diet reduces serum and faecal calprotectin in older adults with increased intestinal permeability: the MaPLE randomised controlled trial. BMC Geriatr (2024). 24(1):707. <https://doi.org/10.1186/s12877-024-05272-y>
59. Tan, F., Zheng, Y., Wang, C., Huang, J., Liu, X., Su, W., et al. Effects of chenpi jiaosu on serum metabolites and intestinal microflora in a dyslipidemia population: a randomized controlled pilot trial. [Front. Endocrinol.](https://www.x-mol.com/paper/journal/2184?r_detail=1905686479325343744) (2025). 16:1552117. <https://doi.org/10.3389/fendo.2025.1552117>
60. Yari, Z., Cheraghpour, M., Alavian, S. M., Hedayati, M., Eini-Zinab, H., Hekmatdoost, A. The efficacy of flaxseed and hesperidin on non-alcoholic fatty liver disease: an open-labeled randomized controlled trial. Eur. J. Clin. Nutr. (2021). 75(1):99-111. <https://doi.org/10.1038/s41430-020-0679-3>
61. Mangel, A. W., Chaturvedi, P. Evaluation of crofelemer in the treatment of diarrhea-predominant irritable bowel syndrome patients. Digestion (2008). 78(4):180-6. <https://doi.org/10.1159/000185719>
62. Moriya, K., Asada, K., Suzuki, S., Enomoto, M., Fujinaga, Y., Tsuji, Y., et al. Benefit of glucosyl hesperidin in patients with primary biliary cholangitis: a multicenter, open-label, randomized control study. Medicine (Baltimore) (2022). 101(48):e32127. <https://doi.org/10.1097/MD.0000000000032127>
63. Zhang, X. X., Chen, W. W., She, B., Luo, R. J., Shi, N., Xue, P., et al. The efficacy and safety of Jian-Wei-Qu-Tong Pills for the treatment of chronic non-atrophic gastritis (spleen and stomach qi deficiency with damp-heat stasis syndrome): study protocol for a phase II, randomized controlled trial. Trials (2014). 15:272. <https://doi.org/10.1186/1745-6215-15-272>
64. Nee, J., Salley, K., Ludwig, A. G., Sommers, T., Ballou, S., Takazawa, E., et al. Randomized clinical trial: crofelemer treatment in women with diarrhea-predominant irritable bowel syndrome. [Clin. Transl. Gastroen.](https://www.x-mol.com/paper/journal/882?r_detail=1390724294592057344) (2019). 10(12):e00110. <https://doi.org/10.14309/ctg.0000000000000110>
65. Chinese University of Hong Kong. (2022). Efficacy and Safety of Using Granules Dendrobii for the Treatment of Chronic Atrophic Gastritis. <https://www.clinicaltrials.gov/study/NCT05209633?cond=Chronic%20Atrophic%20Gastritis&rank=6> [Accessed November 3, 2025]
66. National Cancer Institute, NCI. (2025). Randomized, double-blind, placebo-controlled trial of Meriva® (Curcuminoids) as a candidate chemoprevention agent for gastric carcinogenesis. <https://www.clinicaltrials.gov/study/NCT02782949?cond=Chronic%20Atrophic%20Gastritis&page=2&rank=14> [Accessed November 3, 2025].
67. University of Karachi. (2023). Efficacy and safety of houtou jianweiling tablet in the treatment of chronic non-atrophic Gastritis. <https://www.clinicaltrials.gov/study/NCT04672018?cond=Chronic%20Atrophic%20Gastritis&page=2&rank=16> [Accessed November 3, 2025].
68. Shiraz University of Medical Sciences. (2022). Study on relevant intervention for ulcerative colitis. <https://www.clinicaltrials.gov/study/NCT05117749?term=%20Ulcerative%20Colitis&aggFilters=phase:1%202&rank=2> [Accessed November 3, 2025].
69. Yang, M., Yan, R., Sha, R., Wang, X., Zhou, S., Li, B., et al. Epigallocatechin gallate alleviates non-alcoholic fatty liver disease through the inhibition of the expression and activity of dipeptide kinase 4. Clin. Nutr (2024). 43(8):1769-80. <https://doi.org/10.1016/j.clnu.2024.06.018>
70. Wei, M., Chai, Y., Shen, H., Du, M., Zhou, X., Liu, T., et al. Efficacy and safety of aurantii fructus immaturus flavonoid tablets vs. domperidone for functional dyspepsia: a multicenter, double-blind, double-dummy, randomized controlled phase III trial. AGEB (2024). 87(4):484-93. <https://doi.org/10.51821/87.4.13488>
71. Sangsefidi, Z. S., Hosseinzadeh, M., Ranjbar, A. M., Akhondi-Meybodi, M., Fallahzadeh, H., Mozaffari-Khosravi, H. The effect of total anthocyanin-base standardized (Cornus mas L.) fruit extract on liver function, tumor necrosis factor α, malondealdehyde, and adiponectin in patients with non-alcoholic fatty liver: a study protocol for a double-blind randomized clinical trial. Nutr. J (2019). 18(1):39. <https://doi.org/10.1186/s12937-019-0465-z>
72. Ghanbari, P., Alboebadi, R., Bazyar, H., Raiesi, D., ZareJavid, A., Azadbakht, M. K., et al. Grape seed extract supplementation in non-alcoholic fatty liver disease. Int J Vitam Nutr Res (2024). 94(5-6):365-76. <https://doi.org/10.1024/0300-9831/a000805>
73. Chen, I. J., Liu, C. Y., Chiu, J. P., Hsu, C. H. Therapeutic effect of high-dose green tea extract on weight reduction: a randomized, double-blind, placebo-controlled clinical trial. Clin. Nutr (2016). 35(3):592-9. <https://doi.org/10.1016/j.clnu.2015.05.003>
74. He, A. R., Smith, C. I., Cruz-Correa, M., Chakraborty, R., Zhang, S., Sang, S., et al. A Phase I dose-escalation study of Polyphenon E in liver cirrhosis: evaluation of safety and effect on liver *γ*-OHPdG levels. Cancer Prev. Res. (Phila) (2025). 18(10):635-46. <https://doi.org/10.1158/1940-6207.CAPR-24-0526>
